# RANK drives structured intestinal epithelial expansion during pregnancy

**DOI:** 10.1038/s41586-024-08284-1

**Published:** 2024-12-04

**Authors:** Masahiro Onji, Verena Sigl, Thomas Lendl, Maria Novatchkova, Asier Ullate-Agote, Amanda Andersson-Rolf, Ivona Kozieradzki, Rubina Koglgruber, Tsung-Pin Pai, Dominic Lichtscheidl, Komal Nayak, Matthias Zilbauer, Natalia A. Carranza García, Laura Katharina Sievers, Maren Falk-Paulsen, Shane J. F. Cronin, Astrid Hagelkruys, Shinichiro Sawa, Lisa C. Osborne, Philip Rosenstiel, Manolis Pasparakis, Jürgen Ruland, Hiroshi Takayanagi, Hans Clevers, Bon-Kyoung Koo, Josef M. Penninger

**Affiliations:** 1 Institute of Molecular Biotechnology of the Austrian Academy of Sciences (IMBA), Vienna BioCenter (VBC), Vienna, Austria; 2 Department of Laboratory Medicine, Medical University of Vienna, Vienna, Austria; 3 Institute of Molecular Pathology (IMP), Vienna BioCenter (VBC), Vienna, Austria; 4 Biomedical Engineering Program, Center for Applied Medical Research (CIMA), Universidad de Navarra, Instituto de Investigación Sanitaria de Navarra (IdiSNA), Pamplona, Spain; 5 Oncode Institute, Hubrecht Institute, Royal Netherlands Academy of Arts and Sciences (KNAW) and University Medical Center, Utrecht, The Netherlands; 6 Department of Medical Genetics, Life Sciences Institute, University of British Columbia, Vancouver, British Columbia Canada; 7 Wellcome–MRC Cambridge Stem Cell Institute, University of Cambridge, Cambridge, UK; 8 Department of Paediatrics, University of Cambridge, Cambridge, UK; 9 Department of Paediatric Gastroenterology, Hepatology and Nutrition, Cambridge University Hospitals (CUH), Addenbrooke’s, Cambridge, UK; 10 Department of Microbiology and Immunology, Life Sciences Institute, University of British Columbia, Vancouver, British Columbia Canada; 11 Institute of Clinical Molecular Biology, Kiel University and University Hospital Schleswig-Holstein, Kiel, Germany; 12 Division of Mucosal Immunology, Research Center for Systems Immunology, Medical Institute of Bioregulation, Kyushu University, Fukuoka, Japan; 13 Institute for Genetics and Cologne Excellence Cluster on Cellular Stress Responses in Aging-Associated Diseases (CECAD), University of Cologne, Cologne, Germany; 14 Institute of Clinical Chemistry and Pathobiochemistry, School of Medicine and Health, TUM University Hospital, Munich, Germany; 15 Center for Translational Cancer Research (TranslaTUM), Munich, Germany; 16 Department of Immunology, Graduate School of Medicine and Faculty of Medicine, University of Tokyo, Tokyo, Japan; 17 The Princess Maxima Center for Pediatric Oncology, Utrecht, The Netherlands; 18 Center for Genome Engineering, Institute for Basic Science, Daejeon, Republic of Korea; 19 Helmholtz Centre for Infection Research, Braunschweig, Germany; 20 Present Address: Institute of Human Biology (IHB), Roche Pharma Research and Early Development, Roche innovation Centre, Basel, Switzerland

**Keywords:** Reproductive biology, Small intestine, Genetics research, Intestinal stem cells, Stem-cell research

## Abstract

During reproduction, multiple species such as insects and all mammals undergo extensive physiological and morphological adaptions to ensure health and survival of the mother and optimal development of the offspring. Here we report that the intestinal epithelium undergoes expansion during pregnancy and lactation in mammals. This enlargement of the intestinal surface area results in a novel geometry of expanded villi. Receptor activator of nuclear factor-κΒ (RANK, encoded by *TNFRSF11A*) and its ligand RANKL were identified as a molecular pathway involved in this villous expansion of the small intestine in vivo in mice and in intestinal mouse and human organoids. Mechanistically, RANK–RANKL protects gut epithelial cells from cell death and controls the intestinal stem cell niche through BMP receptor signalling, resulting in the elongation of villi and a prominent increase in the intestinal surface. As a transgenerational consequence, babies born to female mice that lack *Rank* in the intestinal epithelium show reduced weight and develop glucose intolerance after metabolic stress. Whereas gut epithelial remodelling in pregnancy/lactation is reversible, constitutive expression of an active form of RANK is sufficient to drive intestinal expansion followed by loss of villi and stem cells, and prevents the formation of *Apc*^*min*^-driven small intestinal stem cell tumours. These data identify RANK–RANKL as a pathway that drives intestinal epithelial expansion in pregnancy/lactation, one of the most elusive and fundamental tissue remodelling events in mammalian life history and evolution.

## Abstract (web summary)

The RANK–RANKL pathway drives intestinal epithelial expansion in pregnancy and lactation.

## Main

Pregnancy is an important transient physiological state that coincides with substantial metabolic and cellular shifts to support maternal health and optimal growth and survival of offspring^1–3^. This adaptation is a conserved driving force on an evolutionary scale in probably all reproductive species, including insects^4,5^ and mammals^1–3^. Although such adaptation events are critical for the health of mother and offspring, many remodelling aspects are still poorly understood, particularly in mammals. As pregnancy and nourishment of the babies necessitates increased nutritional efficacy in the mothers, a fundamental, unanswered question relates to the cellular and molecular changes of the gut epithelial lining and its orchestration during pregnancy and lactation.

RANKL (encoded by *TNFSF11*, also known as ODF, TRANCE and OPGL) and its receptor RANK (also known as TRANCE-R and ODF-R) are essential for the development and activation of osteoclasts and therefore have a critical role in bone remodelling^6,7^. The RANK–RANKL system also controls lymph node organogenesis^8^, development of thymic medullary epithelial cells^9,10^, central thermoregulation^11^ and, in pregnancy, the formation of a lactating mammary gland^12^ as well as rewiring of the thymic epithelium^13^. Expression of RANKL is strongly induced by inflammatory cytokines and hormones involved in reproduction, such as prolactin and progesterone^14–16^. An imbalance of sex hormones, either due to reduced ovarian function or synthetic progesterone derivatives in hormone replacement therapies and contraceptives, disrupts the RANK–RANKL system and can thereby promote osteoporosis or breast cancer^16^.

Hormones involved in pregnancy and lactation have similarly been implicated in intestinal functions^17^. Notably, osteoprotegerin, a decoy receptor for RANKL, was first cloned from a fetal rat intestinal cDNA library, suggesting that the RANK–RANKL system might also function in the intestine. Indeed, RANK–RANKL is required for the differentiation of microfold cells (M cells) in Peyer’s patches, contributing to microbial sampling and promoting IgA production in the intestine^18–20^. Besides M cells, little is known about the role of intestinal RANK–RANKL. Here we identify RANK and RANKL as regulators of intestinal stem cells and intestinal epithelial expansion in pregnancy and lactation.

## RANK–RANKL control expansion of gut organoids

**Fig. 1:**
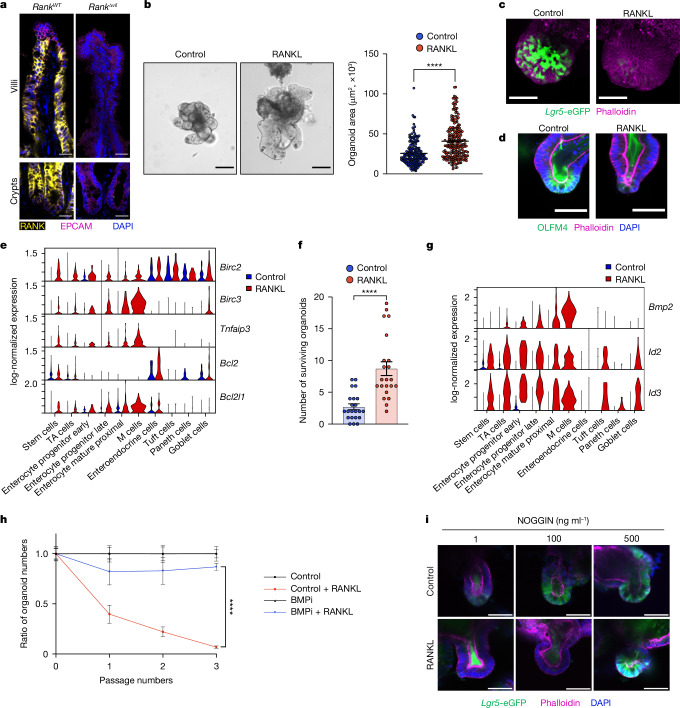
RANK–RANKL drives growth and stem cell exhaustion in mouse intestinal organoids. **a**, RANK and EPCAM intestinal staining in *Rank*^*WT*^ and *Rank*^Δ*vil*^ mice. Scale bars, 25 μm. **b**, Left, representative images of jejunal organoids cultured without (control) and with recombinant mouse RANKL (rmRANKL; 50 ng ml^−1^) for 3 days. Scale bars, 100 μm. Right, quantification of organoid areas after culture with or without rmRANKL for 3 days. *n* = 185 (control) and *n* = 222 (rmRANKL) from three independent experiments. **c**, Representative 3D images of *Lgr5-eGFP;Ires-cre*^*ER/+*^ organoids. Scale bars, 50 μm. **d**, Representative images of OLFM4 staining of control and rmRANKL-treated organoids. Scale bars, 50 μm. **e**, Single-cell log-normalized expression of the indicated anti-apoptotic genes (*y* axis) in each cell type (*x* axis) (control jejunal organoids versus organoids cultured with 50 ng ml^−1^ rmRANKL for 12 h). **f**, Organoids treated with or without rmRANKL (50 ng ml^−1^) were irradiated and cultured in WENR medium with a ROCK inhibitor (Y-27632; 10 μM) (see Methods) for 7 days. The numbers of surviving organoids from three independent experiments are shown. *n* = 22 (control and rmRANKL). **g**, Single-cell log-normalized expression of *Bmp2* and the BMP targeted genes *Id2* and *Id3* (*y* axis) in each cell type (*x* axis). **h**, The ratio of organoid numbers cultured in the presence of rmRANKL (50 ng ml^−1^), DMSO (control) or with the BMP inhibitor (BMPi) LDN193189 (0.5 μM). The ratio of organoid numbers in the control + RANKL group was normalized to the control group, whereas the ratio of organoid numbers in the iBMP + RANKL group was normalized to the iBMP group. Data are combined from two experiments. *n* = 10 for each group shown. **i**, Representative images of rmRANKL-treated *Lgr5-eGFP;Ires-cre*^*ER/+*^ organoids that were cultured with recombinant mouse NOGGIN. Scale bars, 50 μm. In the indicated images, phalloidin stains actin filaments and DAPI stains nuclei. Data are mean ± s.e.m. Statistical analysis was performed using two-tailed Student’s *t*-tests (**b** and **f**), two-sided Wilcoxon rank-sum tests between samples, adjusted using Benjamini–Hochberg correction (**e** and **g**) and one-way analysis of variance (ANOVA) with Tukey’s post hoc test (**h**); ****P *<* 0.0001. Further details on statistics and reproducibility are provided in the Methods. Source Data

**Extended Data Fig. 1:**
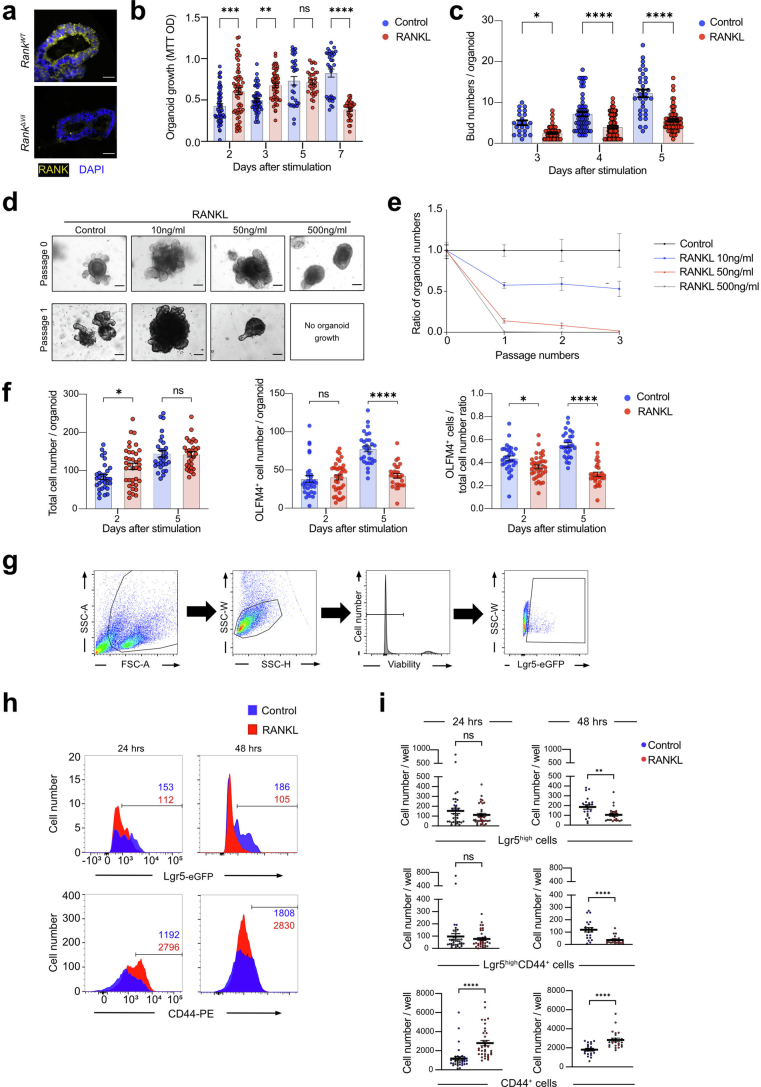
Characterization of stem cells in RANK–RANKL-stimulated mouse intestinal organoids. **a**, Representative images of anti-RANK immunostaining in mouse jejunal intestinal organoids from *Rank*^*WT*^ and *Rank*^*ΔVil*^ mice. DAPI is shown to visualize nuclei. Scale bars, 50 μm. **b**, Proliferation assay in organoids without (control) or in the presence of recombinant mouse RANKL (rmRANKL; 50 ng/ml) as determined by an MTT assay at the indicated time points. Each plot represents MTT OD, pooled from at least two independent experiments. Group numbers at 2 days: *n* = 56 (control), *n* = 56 (rmRANKL); 3 days: *n* = 50 (control), *n* = 50 (rmRANKL); 5 days: *n* = 31 (control), *n* = 31 (rmRANKL) and 7 days: *n* = 32 (control), *n* = 31 (rmRANKL). **c**, Numbers of buds per intestinal organoid cultured in ENR medium with/without rmRANKL. Data were combined from three independent experiments. Group numbers at 3 days: *n* = 22 (control), *n* = 59 (rmRANKL); 4 days: *n* = 57 (control), *n* = 102 (rmRANKL) and 5 days: *n* = 32 (control), *n* = 66 (rmRANKL) per group. **d**, Representative images of jejunal organoids at passage 0 and passage 1 cultured in the absence (control) or presence of the indicated concentrations of rmRANKL. Scale bars, 100 μm. **e**, Ratios of organoid numbers after prolonged passaging in the absence (control) and presence of rmRANKL. Numbers of organoids were counted at each passage. Data from two independent experiments are shown. *n* = 8 (control), *n* = 8 (10 ng/ml RANKL), *n* = 8 (50 ng/ml RANKL), *n* = 8 (500 ng/ml RANKL). **f**, Total cell numbers, OLFM4 positive cells per organoid and ratios of OLFM4 positive cell in relation to the total cell number per each jejunal organoid cultured in ENR medium with/without rmRANKL. Data were combined from two independent experiments. 2 days: *n* = 31 (control), *n* = 37 (rmRANKL); 5 days: *n* = 29 (control), *n* = 29 (rmRANKL) per group. **g**, Gating strategy for detecting Lgr5-eGFP^+^ cells using fluorescence-activated cell sorting (FACS). Viability was determined using the viability-dye described in the Methods. FSC, forward scatter; SSC, side scatter. **h**, Representative FACS histograms of Lgr5^high^ cells and CD44^+^ cells isolated from *Lgr5-eGFPiresCreER/+* jejunal organoids cultured without (control) and with rmRANKL (50 ng/ml) for the indicated times. Numbers of Lgr5^high^ cells and CD44^+^ cells among total viable organoid cells are indicated for each group. Data are representative of at least two independent experiments. **i**, Quantification of Lgr5^high^, Lgr5^high^CD44^+^, and CD44^+^ cells per well in *Lgr5-eGFPiresCreER/+* jejunal organoids cultured without (control) or with rmRANKL (50 ng/ml) for the indicated times. *n* = 36 (Control, 24hrs), *n* = 36 (RANKL, 24hrs), *n* = 21 (Control, 48hrs), *n* = 24 (RANKL, 48hrs). Data are mean ± s.e.m. *P < 0.05; **P < 0.01; ***P < 0.001; ****P < 0.0001; ns, not significant. One-way analysis of variance (ANOVA) with Tukey’s post hoc test (**b,c,f**); Two-tailed Student’s t-test (**i**). More details on statistics and reproducibility can be found in the Methods. Source Data

We assessed RANK expression in the intestine. RANK was found on the cell surface of almost all mouse intestinal epithelial cells in the intestine including differentiated enterocytes and stem cells, except for epithelial cells located at the top of villi (Fig. 1a and Extended Data Fig. 1a). To directly test whether RANK has a functional role in the intestinal epithelium, we exposed mouse intestinal organoids to RANKL. Stimulating jejunal organoids with RANKL enhanced their growth; however, at around day 3, the numbers of organoid buds decreased, and we observed aberrant budding elongation (Fig. 1b and Extended Data Fig. 1b,c). Organoids can be passaged nearly indefinitely as they contain functional stem cells that correlate with bud numbers^21^. RANKL stimulation resulted in markedly decreased passaging capacities, and a complete inability to maintain organoids beyond passage 3, depending on the dose of RANKL (Extended Data Fig. 1d,e). We demonstrated that RANKL treatment resulted in an initial expansion of CD44^+^ cells, followed by a marked decrease in *Lgr5*-eGFP^+^CD44^+^ and OLFM4^+^ stem cells (Fig. 1c,d and Extended Data Fig. 1f–h), providing a cellular correlate for the impaired passaging capacity. RANKL-driven changes can be observed in gut organoids from male and female mice. RANK activation of mouse intestinal organoids results in early growth stimulation followed by stem cell exhaustion and/or enhanced terminal differentiation.

**Extended Data Fig. 2:**
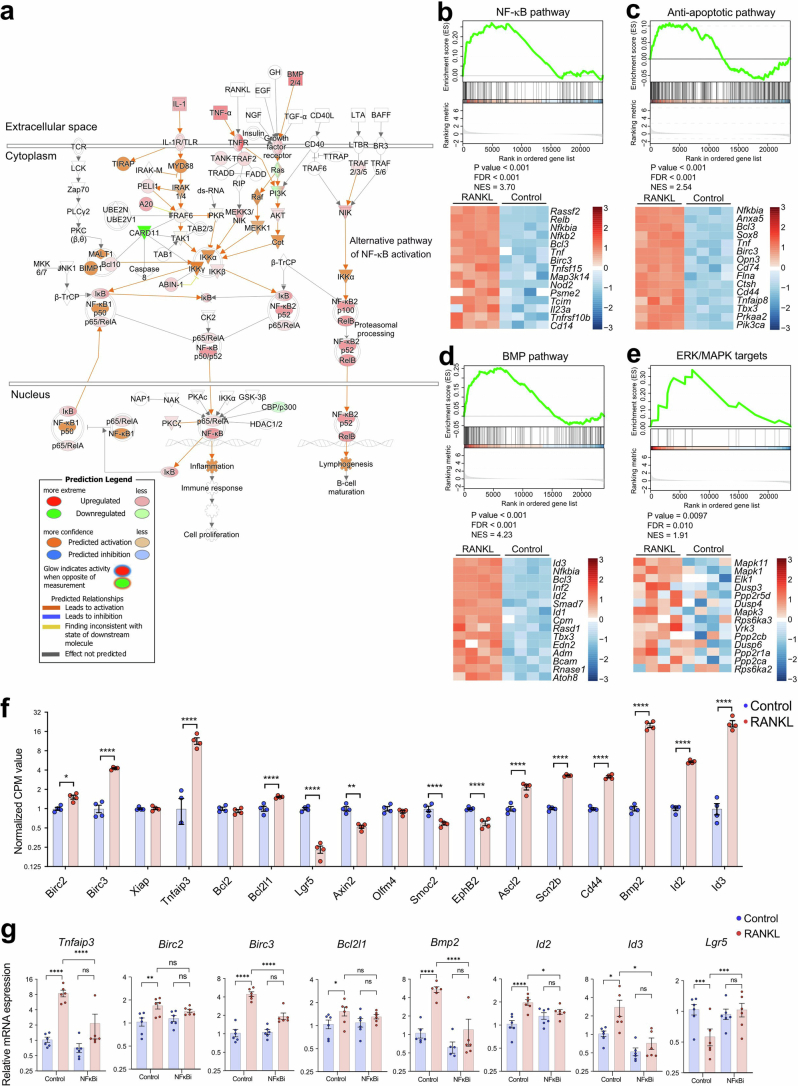
Bulk RNA-seq profiling of RANK–RANKL-stimulated mouse intestinal organoids. **a-e**, RANK–RANKL-induced gene expression changes in jejunal organoids without (control, n = 4) and with rmRANKL (n = 4) stimulation. Total RNA was isolated 12 hrs after addition of rmRANKL (50 ng/ml) and processed for bulk RNA-seq. **a**, RANK–RANKL-triggered expression changes in NF-κB signalling as assessed by Ingenuity Pathway Analysis. The node colours indicate changes in expression levels of the indicated genes, determined by DESeq2 (p < 0,05). Red, up-regulated gene expression in RANKL treated samples; green, down-regulated gene expression; white, no expression changes; orange, Ingenuity predicted activation. Orange arrows indicate activation of the specified downstream signalling pathways; grey arrows indicate previously reported connections, albeit these pathways were not identified in our data set using Ingenuity. The Ingenuity enrichment statistics for this pathway were determined using Fischer’s Exact Test p-value with Benjamin–Hochberg correction, 4.93E-08; Z score, 2,722. **b-e**, RANK–RANKL-induced changes of the NF-κB, anti-apoptotic, BMP, and ERK/MAPK pathways as assessed by Gene set enrichment analysis (GSEA) enrichment plots (top panels). Bottom panels show heatmaps of the top 15 genes upregulated in response to RANKL stimulation. Expression profiles of RANKL-stimulated jejunal organoids were compared to non-stimulated (control) jejunal organoids cultured for the same time periods. **f**, Differential gene expression analysis of RNAseq data from mouse jejunal organoids without (control) and with rmRANKL stimulation. RNA was isolated 12 h after addition of rmRANKL (50 ng/ml). Normalized CPM values of selected anti-apoptotic genes, stem cell signature genes and BMP signalling genes are shown. Individual data points are shown (n = 4/4). **g**, Quantitative RT–PCR analyses to compare expression levels of anti-apoptotic genes, stem cell signature genes and BMP signalling genes in mouse jejunal organoids. Data represent the relative expression of the indicated genes in jejunal organoids cultured in the presence of rmRANKL (50 ng/ml) without (control, DMSO solvent) or with the NFκB inhibitor (NFκBi) sc-514 (100 mM). Gene expression was compared to control (no RANKL treatment) organoids (set at 1). *n* = 6 independent jejunal organoids were analysed for each group. rmRANKL stimulation was done for 12 h. Data are mean ± s.e.m. *P < 0.05; **P < 0.01; ***P < 0.001; ****P < 0.0001; ns, not significant. Enrichment-adjusted p-values (P value), False Discovery Rates (FDR), and Normalized Enrichment Scores (NES) were calculated using two-sided fast pre-ranked gene set enrichment analysis (fGSEA) (**b-e**). Two-sided DESeq2 Wald tests, adjusted with the Benjamini–Hochberg procedure (**f**); Repeated measure One-way analysis of variance (ANOVA) with Tukey’s post hoc test (**g**). Source Data

**Extended Data Fig. 3:**
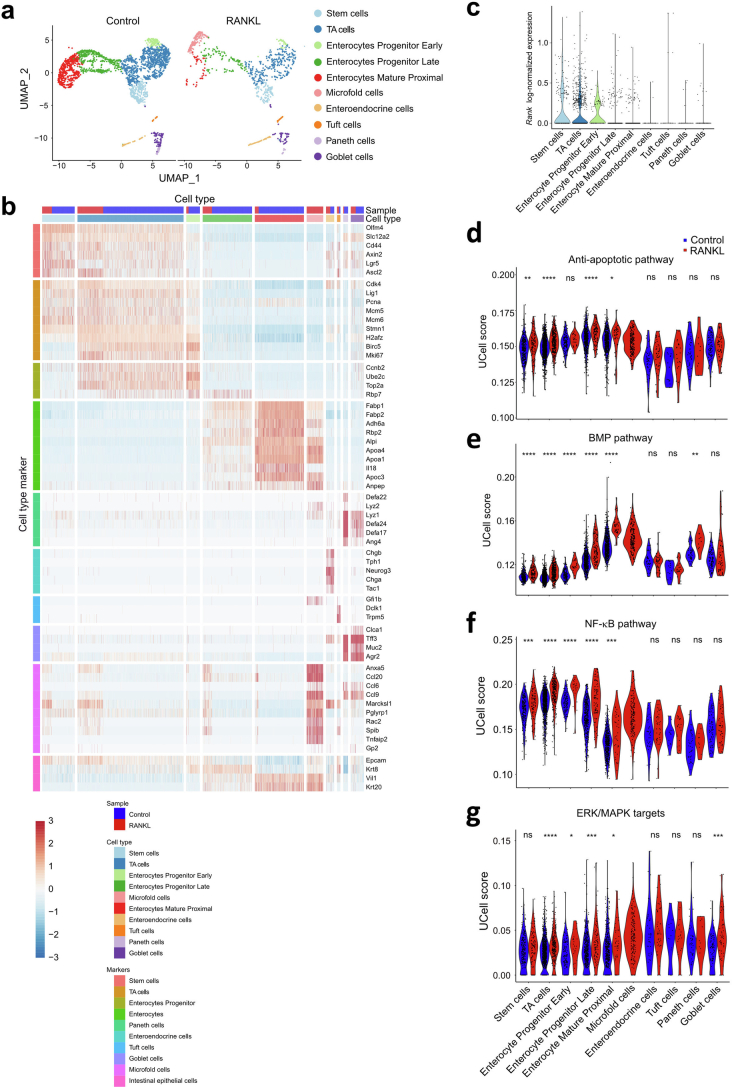
Single-cell RNA-seq profiling of RANK–RANKL-stimulated mouse jejunal organoids. **a**, Uniform manifold approximation and projection (UMAP) of 3,143 cells from both control intestinal organoids (2,299 cells) and organoids cultured in the presence of rmRANKL (50 ng/ml) for 12 hrs (844 cells). Cells are colour coded according to their cell-type annotation using unsupervised clustering. **b**, Heatmap representing scaled single cell log-normalized gene expression of markers for epithelial cell-types taken from published cell-type signatures (Yum et al.^26^, Haber et al.^22^ and Biton et al.^59^). Columns are ordered by the indicated cell-type annotations and sample of origin (control mouse jejunal organoids versus jejunal organoids cultured with 50 ng/ml rmRANKL for 12 hrs). The marker genes (rows) are grouped by cell-type. **c**, *Rank* mRNA expression in jejunal organoid subpopulations. Violin plots show single cell log-normalized expression of *Rank* mRNA in each of the indicated cell-types for control (not stimulated with rmRANKL) organoids. Each dot represents an individual cell. **d**, RANK/RANKL stimulation induces anti-apoptotic genes. Violin plots show single cell RNAseq UCell scores for apoptotic gene sets (GO:0043066) in the indicated organoid cell-types. **e**, Violin plots showing single cell RNAseq UCell scores for the BMP signalling pathway gene set^26^ in the indicted organoid cell subsets. **f**, Induction of the NF-κB pathway in response to rmRANKL (50 ng/ml, 12 hrs) as compared to non-treated jejunal organoids (control). Violin plots show UCell scores for the NF-κB pathway gene set (GO:0038061) in each cell-type. **g**, Induction of ERK/MAPK target genes in control and rmRANKL-stimulated (50 ng/ml, 12 hrs) jejunal organoids. Violin plots show UCell scores for the ERK/MAPK target gene set obtained from Reactome (Reactome_M13408) in each organoid cell-type. *P < 0.05; ***P < 0.001; ****P < 0.0001; ns, not significant. Two-sided Wilcoxon Rank Sum test between samples, adjusted with the Benjamini–Hochberg procedure (**d-g**).

We next performed gene expression profiling using bulk RNA-sequencing (RNA-seq). RANK stimulation of small intestinal organoids resulted in activation of the NF-κB pathway and induction of multiple anti-apoptotic genes (Extended Data Fig. 2a–c). It also resulted in upregulation of the BMP and MAPK–ERK pathways (Extended Data Fig. 2d,e). RANK-dependent induction of the BMP pathway, in particular *Bmp2* and the BMP2 downstream genes *Id2* and *Id3*, and anti-apoptotic genes such as *Birc2*, *Birc3*, *Tnfaip3* and *Bcl2l1* was confirmed using quantitative PCR (qPCR; Extended Data Fig. 2f,g). By contrast, RANKL treatment of organoids suppressed expression of the stem cell signature genes *Lgr5*, *Smoc2*, *Ephb2* or *Axin2*. Notably, expression of some progenitor cell markers such as *Scn2b*, *Ascl2* or *Cd44* was induced by RANK stimulation (Extended Data Fig. 2f,g). Single-cell RNA-seq (scRNA-seq) profiling of control and RANK-stimulated mouse organoids stratified nine distinct epithelial populations^22^. After RANK stimulation, we observed an additional population constituting M cells (Extended Data Fig. 3a,b) as reported previously^22,23^. In control organoids, *Rank* mRNA expression was found primarily in progenitor cell populations (Extended Data Fig. 3c), indicating, together with our protein expression data, that RANK is transcribed in intestinal progenitors and RANK protein is subsequently maintained in the differentiated intestinal epithelium. Induction of anti-apoptotic genes was detected in stem cells, transit-amplifying (TA) cells and absorptive enterocyte lineage cells (Fig. 1e and Extended Data Fig. 3d). RANKL-treated organoids exhibited enhanced survival in response to radiation injury (Fig. 1f). The marked upregulation of BMP2 was primarily observed in enterocyte (immature as well as mature) progenitors, but was also found at low levels in cycling stem cells; the BMP2-regulated target genes *Id2* and *Id3* were prominently induced in stem cells, TA cells and absorptive enterocytes (Fig. 1g and Extended Data Fig. 3e). Moreover, similar to bulk RNA-seq we observed enhanced NF-κB as well as increased MAPK–ERK pathway activation in these intestinal cell populations, with the exception of enteroendocrine, Paneth and tuft cells (Extended Data Fig. 3f,g).

**Extended Data Fig. 4:**
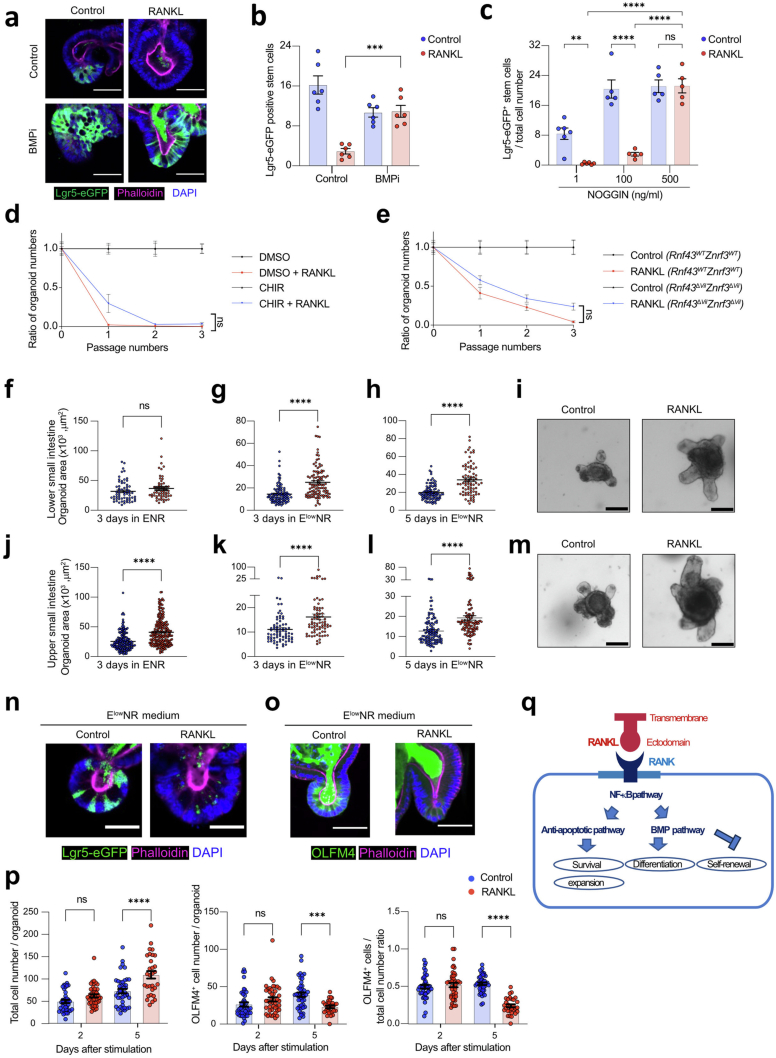
The key pathways for RANK–RANKL-driven stem cell exhaustion in mouse intestinal organoids. **a**, Left panels, representative images of rmRANKL-treated (50 ng/ml) *Lgr5-eGFPiresCre*^*ER/+*^ organoids cultured without (control, DMSO solvent) or with the BMPi LDN193189 (0,5μM). Phalloidin indicates actin filaments, and DAPI shows nuclei. Scale bars, 50 μm. **b**, Quantification of the ratio of Lgr5^+^ cells per well in *Lgr5-eGFPiresCreER/+* jejunal organoids cultured in the presence of rmRANKL (50 ng/ml) without (control, DMSO solvent) or with the BMP inhibitor (BMPi) LDN193189 (0,5μM) using FACS. Data points represent individual wells from at least 2 independent experiments. *n* = 6 (Control), *n* = 6 (Control+RANKL), *n* = 6 (BMPi), *n* = 6 (BMPi+RANKL). **c**, Quantification of the ratio of Lgr5^+^ cells per well in *Lgr5-eGFPiresCreER/+* jejunal organoids cultured in the presence of rmRANKL (50 ng/ml) together with 1, 100, or 500 ng/ml of rmNOGGIN, determined using FACS. Data points represent individual wells pooled from at least 2 independent experiments. *n* = 6 (1 ng/ml NOGGIN), *n* = 6 (1 ng/ml NOGGIN + RANKL), *n* = 5 (100 ng/ml NOGGIN), *n* = 5 (100 ng/ml NOGGIN + RANKL), *n* = 5 (500 ng/ml NOGGIN), *n* = 5 (500 ng/ml NOGGIN + RANKL). **d**, Ratios of mouse jejunal organoid numbers cultured in the presence of rmRANKL (50 ng/ml) without (control, DMSO solvent) or with the GSK3 inhibitor CHIR 99021 (20μM). The numbers of organoids were counted at each passage. The ratio of organoid numbers in the Control + RANKL group was normalized to control organoids, whereas the ratio of organoid numbers in the CHIR + RANKL treated group was normalized to CHIR only treated organoids. Data are combined from 2 independent experiments. *n* = 11 for each group shown. **e**, Ratios of numbers of jejunal organoids from *Rnf43*^*WT*^*Znrf3*^*WT*^ and *Rnf43*^*ΔVil*^*Znrf3*^*ΔVil*^ mice after prolonged culture in the presence of rmRANKL (50 ng/ml). The numbers of organoids were counted at each passage. The ratio of organoid numbers in the RANKL treated *Rnf43*^*WT*^*Znrf3*^*WT*^ organoids was normalized to untreated control *Rnf43*^*WT*^*Znrf3*^*WT*^ organoids, whereas the ratio of organoid numbers in the RANKL treated *Rnf43*^*ΔVil*^*Znrf3*^*ΔVil*^ group was normalized to control *Rnf43*^*ΔVil*^*Znrf3*^*ΔVil*^ organoids. Data are from 2 independent experiments. *n* = 11 for each group shown. **f**, Sizes of mouse intestinal organoids generated from the lower small intestine (ileum) cultured in ENR medium (50 ng/ml EGF) with/without rmRANKL (50 ng/ml) for 3 days; *n* = 63 (control), *n* = 58 (rmRANKL) per group. Data are from 3 independent experiments. **g,h**, Sizes of intestinal organoids generated from the ileum cultured in EGF-reduced E^low^NR medium (50 pg/ml EGF) with/without rmRANKL (50 ng/ml) for 3 days (*n* = 99 (control), *n* = 104 (rmRANKL)) and 5 days (*n* = 89 (control), *n* = 78 (rmRANKL)). Data are from 3 independent experiments. **i**, Representative images of intestinal organoids generated from the ileum cultured in E^low^NR medium with/without rmRANKL for 5 days. Scale bars, 100 μm. **j**, Sizes of intestinal organoids generated from upper small intestine (jejunum) cultured in ENR medium (50 ng/ml EGF) with/without rmRANKL (50 ng/ml) for 3 days; *n* = 185 (control), *n* = 222 (rmRANKL). Data are combined from 3 independent experiments. Of note, these are the same data as shown in Fig. 1b, for better comparison. **k,l**, Sizes of intestinal organoids generated from the jejunum cultured in E^low^NR medium (50 pg/ml EGF) with/without rmRANKL (50 ng/ml) for 3 days (*n* = 74 (control), *n* = 76 (rmRANKL) per group) and 5 days (*n* = 113 (control), *n* = 105 (rmRANKL) per group). Data are combined from 3 independent experiments. **m**, Representative images of jejunal organoids cultured in E^low^NR medium with/without rmRANK for 5 days. Scale bars, 100 μm. **n**, Representative images of *Lgr5eGFPiresCreER/+* jejunal organoids cultured in E^low^NR medium with/without rmRANK for 5 days. Lgr5 positive stem cells are shown in green. Phalloidin (magenta) and DAPI (blue) are shown to visualize F-actin and nuclei, respectively. Scale bars, 25 μm. **o**, Representative images of OLFM4 immunostaining of jejunal organoids cultured in E^low^NR medium with/without rmRANK for 5 days. Phalloidin and DAPI are shown to visualize F-actin and nuclei, respectively. Note secreted OLFM4 in the lumen of the organoids as reported previously^60^. Scale bars, 50 μm. **p**, Total cell numbers, numbers of OLFM4+ cells and ratios of OLFM4+ cells to total cell numbers per jejunal organoid cultured in E^low^NR medium with/without rmRANKL for 2 days (*n* = 43 (control), *n* = 44 (rmRANKL)) and 5 days (*n* = 44 (control), *n* = 30 (rmRANKL)). Data were combined from at least 2 independent experiments. **q**, Proposed mechanism for RANKL-induced cell survival of intestinal epithelial cells via induction of anti-apoptotic genes and NF-κB signalling and intestinal stem cell exhaustion via induction of BMP2. Data are mean ± s.e.m. **P < 0.01; ***P < 0.001; ****P < 0.0001; ns, not significant. One-way analysis of variance (ANOVA) with Tukey’s post hoc test (**b,c, d,e,p**); Two-tailed Student’s t-test (**f-h, j-l**). More details on statistics and reproducibility can be found in the Methods. Source Data

The BMP pathway is known to inhibit intestinal stem cell maintenance^24,25^. To assess whether *Bmp2* upregulation might be responsible for the observed phenotype, we treated RANK-stimulated organoids with the BMP receptor-blocker LDN-193189 (BMPi)^26^ as well as with NOGGIN, which antagonizes BMP signalling^21^. BMPi treatment rescued the stem cell exhaustion phenotype of RANKL-treated organoids and the intestinal organoids could be maintained in culture (Fig. 1h and Extended Data Fig. 4a,b). NOGGIN also alleviated RANK-mediated stem cell exhaustion in a dose-dependent manner (Fig. 1i and Extended Data Fig. 4c). We next investigated whether RANK-induced stem cell exhaustion is caused by the attenuation of WNT β-catenin signalling, which is critical for stem cell renewal. Inhibition of GSK3β, which negatively regulates WNT–β-catenin signalling^27^, using the drug CHIR99021 had only a minor impact on the detrimental effects of RANK–RANKL on intestinal stem cell maintenance (Extended Data Fig. 4d). We also treated organoids from *Rnf43*/*Znrf3* double-mutant mice; these double-mutant mice show enhanced WNT signalling in the intestine^28^. We again observed no rescue of the RANK-induced stem cell exhaustion in *Rnf43*/*Znrf3* double-mutant organoids (Extended Data Fig. 4e). Besides WNT, EGFR signalling has been shown to drive proliferation of intestinal stem cells^21^. At low EGF levels, we observed an even stronger RANKL effect on proliferation and growth, particularly in organoids generated from the lower ileum (Extended Data Fig. 4f–m). However, RANKL treatment still induced stem cell exhaustion irrespective of low or high EGF levels (Extended Data Fig. 4n–p). These data show that RANK–RANKL stimulation alters the stem cell niche through BMP2 signalling and enhances anti-apoptotic pathways as well as cell proliferation, resulting first in larger organoids of differentiated intestinal epithelial cells but, after prolonged stimulation, resulting in reduced stem cell numbers and disrupted organoid maintenance (Extended Data Fig. 4q).

## Constitutive activation of RANK in vivo

**Fig. 2:**
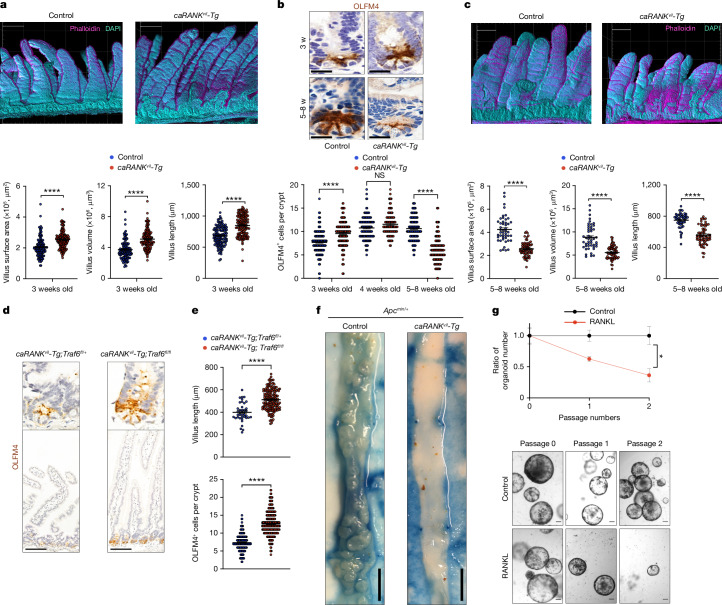
Constitutive activation of RANK in vivo modulates the intestinal stem cell niche. **a**, Representative 3D reconstruction of small intestine from 3-week-old control and *caRANK*^*vil*^*-Tg* mice (top). Grid spacing is 200 μm. Bottom, villus length, volume and surface areas of control (*n* = 6 mice, *n* = 126 villi) and *caRANK*^*vil*^*-Tg* mice (*n* = 7 mice, *n* = 145 villi analysed). **b**, Representative OLFM4 immunostaining in the small intestines of 3- and 5–8-week-old control and *caRANK*^*vil*^*-Tg* mice (top). Scale bars, 20 μm. Bottom, the numbers of OLFM4^+^ cells per crypt in control and *caRANK*^*vil*^*-Tg* mice were quantified at 3 weeks (*n* = 3 mice, *n* = 234 crypts (control); *n* = 3 mice, *n* = 205 crypts (*caRANK*^*vil*^*-Tg*)), 4 weeks (*n* = 6 mice, *n* = 162 crypts (control); *n* = 4 mice and *n* = 110 crypts (*caRANK*^*vil*^*-Tg*)) and at 5–8 weeks (*n* = 4 mice, *n* = 203 crypts (control); *n* = 4 mice, *n* = 178 crypts (*caRANK*^*vil*^*-Tg*)) of age. **c**, Representative 3D reconstruction of the small intestines of 5–8-week-old control and *caRANK*^*vil*^*-Tg* mice (top). Grid spacing is 200 μm. Bottom, the villus length, volume and surface areas of control (*n* = 4 mice, *n* = 47 villi) and *caRANK*^*vil*^*-Tg* mice (*n* = 5 mice, *n* = 65 villi). **d**, Representative OLFM4 immunostaining in the small intestines of 8-week-old *caRANK*^*vil*^*-Tg;Traf6*^*fl/+*^ (*n* = 3) and *caRANK*^*vil*^*-Tg;Traf6*^*fl/fl*^ (*n* = 5) mice. Scale bars, 100 μm. **e**, The numbers of OLFM4^+^ cells per crypt and the villus length in *caRANK*^*vil*^*-Tg;Traf6*^*fl/+*^ (*n* = 3 mice, 172 crypts, 40 villi) and *caRANK*^*vil*^*-Tg; Traf6*^*fl/fl*^ (*n* = 5 mice, 299 crypts, 167 villi) mice. **f**, Representative macroscopic images of the small intestines of *Apc*^*min/+*^ and *caRANK*^*vil*^*-Tg;Apc*^*min/+*^ mice. Scale bars, 7.5 mm. **g**, The ratio of tumoroid numbers cultured without (control) or with rmRANKL (50 ng ml^−1^) (top). The ratio of organoid numbers in the RANKL-treated group was normalized to the control group. Data were combined from two independent experiments. *n* = 4 (control) and *n* = 4 (rmRANKL). Bottom, representative images of tumoroids established from *Apc*^*min/+*^ mice at passage 0, 1 and 2, cultured in the absence (control) or presence of rmRANKL (50 ng ml^−1^). Scale bars, 100 μm. Each point represents the measurement of length, volume and surface area (**a** (bottom), **c** (bottom) and **e** (top)), and the number of OLFM4^+^ cells per crypt (**b** (bottom) and **e** (bottom)). Data are mean ± s.e.m. *P < 0.05; NS, not significant. Statistical analysis was performed using two-tailed Student’s *t*-tests (**a**, **c**, **e** and **g**) and one-way ANOVA with Tukey’s post hoc test (**b**). Further details on statistics and reproducibility are provided in the Methods. Source Data

**Extended Data Fig. 5:**
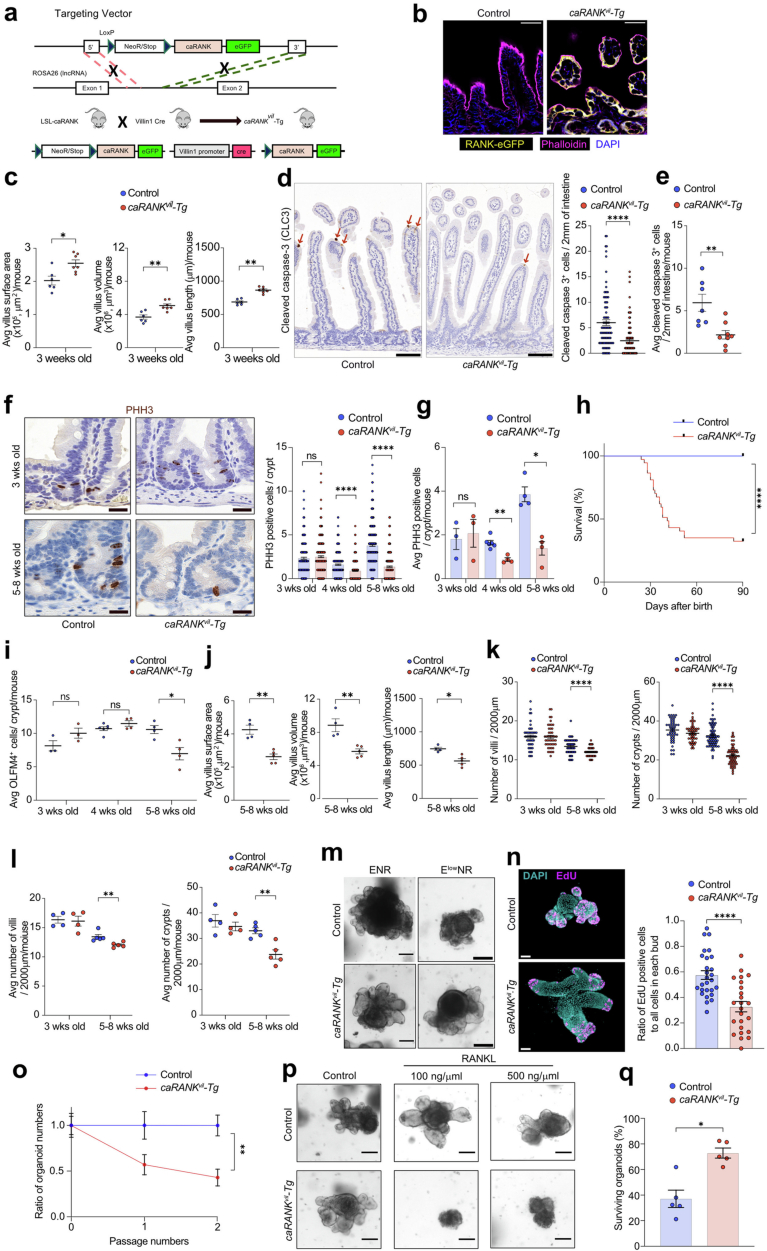
The in vivo effects of constitutive activation of RANK in intestinal epithelium. **a**, Schematic outline of transgenic mice that conditionally express a constitutively active *RANK* mutant in the *ROSA26* locus using gene targeting, termed *LSL-caRANK* mice. Crossing this line with *Villin1Cre* mice removes the stop cassette and thereby drives specific expression of the RANK transgene in the intestinal epithelium; these mice are termed *caRANK*^*vil-Tg*^. LSL, LoxP-STOP-LoxP; NeoR, neomycin resistance cassette; eGFP, enhanced green fluorescence protein. **b**, Representative images of eGFP expression in intestinal epithelial cells of *caRANK*^*vil-Tg*^ mice and control mice, using cryo-sections. Phalloidin and DAPI are shown to visualize F-actin and nuclei, respectively. Scale bars, 50 μm. **c**, Small intestinal villi length, volume and surface areas of 3-week-old control (n = 6 mice, n = 126 villi analysed) and *caRANK*^*vil*^*-Tg* mice (n = 7/145). Each data point represents the average length, volume, and surface area of villi per mouse. The original data is the same as Fig. 2a. **d,e**, Reduced cell death of small intestinal epithelial cells in *caRANK*^*vil-Tg*^ mice. Representative images (left in **d**) and quantification (right in **d**, **e**) of cleaved caspase-3 (CLC3) immunostaining (arrows) in the small intestine are shown for three wks old control mice (n = 7 mice, n = 93 regions analysed) and *caRANK*^*vil*^*-Tg* mice (n = 8/126). Each data point represents the number of CLC3 positive cells in villi per 2 mm of the intestine (**d**) and the average number per mouse (**e**). Scale bars, 100 μm. **f,g**, Representative images (left in **f**) and quantification of phosphor-Histone H3 (pHH3) immunostaining in the crypts of the upper small intestines from control and *caRANK*^*vil*^*-Tg* mice (right in **f**, **g**). 3 wks old control mice (n = 3 mice, n = 254 crypts analysed) and *caRANK*^*vil*^*-Tg* mice (n = 4/395); 4 wks old control (n = 6/379) and *caRANK*^*vil*^*-Tg* mice (n = 4/351); and 5–8 wks old control (n = 4/556) and *caRANK*^*vil*^*-Tg* littermates (n = 4/487). Each data point represents the number of pHH3 positive cells per individual crypts (**f**) and average number per mouse (**g**). Scale bars, 20 μm. **h**, Kaplan–Meier survival curve of control (*n* = 31) and *caRANK*^*vil*^*-Tg* (*n* = 37) littermates. **i**, Numbers of OLFM4^+^ cells/ crypt in the indicated mice. Each data point represents the average length, volume, and surface area of villi per mouse in the indicated mice described in Fig. 2b. **j**, Small intestinal villi length, volume and surface areas the indicated mice. Each data point represents the averaged length, volume, and surface area of villi per mouse. The original data is the same as Fig. 2c. **k,l**, Numbers of villi (left) and crypts (right) per 2 mm were quantified in histological H&E sections of the upper small intestine from control (n = 4 mice, n = 48 regions) and *caRANK*^*vil*^*-Tg* (n = 4/46) mice at 3 and control (n = 5 mice, n = 63 regions) and *caRANK*^*vil*^*-Tg* (n = 5/56) mice at 3 and 5–8 wks of age. Each data point represents the number of Numbers of villi (left) and crypts (right) per 2 mm of the intestine (**k**) and the average number per mouse (**l**). **m**, Representative images of mouse jejunal organoids from 2 weeks old control *caRANK*^*vil*^*-Tg* mice, cultured in E^low^NR (50 pg/ml EGF) or ENR (50 ng/ml EGF) media for 3 days after the first passage. Scale bars, 100 μm. **n**, Representative images (left) of EdU^+^ proliferating cells in control small intestinal organoids (n = 7) and *caRANK*^*vil*^*-Tg* mice-derived organoids (n = 6). Scale bars, 50 μm. Right; ratios of EdU labelled proliferating cells to all cells presents in each bud. The analysed bud number is n = 27 (control) and n = 24 (*caRANK*^*vil*^*-Tg*). **o**, Ratios of organoid numbers after prolonged culture of control- and *caRANK*^*vil*^*-Tg* mice-derived jejunal organoids. Numbers of organoids were counted at each passage. The ratio of organoid numbers in the *caRANK*^*vil*^*-Tg* mice-derived jejunal organoids was normalized to untreated control jejunal organoids. Data were combined from two independent experiments. n = 6 (control), n = 6 (*caRANK*^*vil*^*-Tg*). **p**, Representative images of control and *caRANK*^*vil*^*-Tg* mice-derived small intestinal organoids cultured with/without recombinant human RANKL (rhRANKL; 100, 500 ng/ml) for 4 days. Scale bars, 100 μm. **q**, Percentages of surviving control and *caRANK*^*vil*^*-Tg* jejunal organoids after irradiation, as compared to non-irradiated organoids of the same genotypes. Organoids were cultured in ENR medium and irradiated with 4 Gy, followed by subsequent culture for 7 days. Data are from two independent experiments. n = 5 (control), n = 5 (*caRANK*^*vil*^*-Tg*). AVG, average. Data are mean ± s.e.m. *P < 0.05; **P < 0.01; ****P < 0.0001; ns, not significant. Two-tailed Mann–Whitney *U*-test (**c,e,g,i,j,l,o,q);** Kaplan–Meier survival curve with a log-rank test (**h**); Two-tailed Student’s t-test (**d,f,k,n**). Source Data

To investigate whether prolonged RANK activation leads to intestinal stem cell exhaustion in vivo, we generated a transgenic mouse line that conditionally expresses a constitutively active *RANK* mutant (*Rosa26-LSL-caRANK*) in the intestinal epithelium using *Villin1*-promoter-driven Cre (*Villin1-cre*) (hereafter, *caRANK*^*vil-Tg*^ mice) (Extended Data Fig. 5a,b). This activating mutation in the cytosolic domain (amino acid 240) of human RANK was recently identified in a patient with malignant lymphoma^29^. The *caRANK*^*vil-Tg*^ mice appeared to be normal at birth and initially exhibited normal growth until weaning age. At 3 weeks of age, *caRANK*^*vil-Tg*^ mice exhibited a marked expansion of small intestinal villi; 3D reconstruction revealed increased villous length and enhanced villous volumes and surface areas (Fig. 2a and Extended Data Fig. 5c). Immunostaining showed reduced apoptosis in villi of 3-week-old *caRANK*^*vil-Tg*^ mice compared with the controls (Extended Data Fig. 5d,e). Proliferation of in vivo phosphorylated histone H3 labelled crypt cells appeared normal at 3 weeks of age (Extended Data Fig. 5f,g). From weaning age, *caRANK*^*vil-Tg*^ mice progressively lost weight and needed to be euthanized due to excessive weight loss (Extended Data Fig. 5h). In the older *caRANK*^*vil-Tg*^ mice, we observed reduced numbers of OLFM4^+^ intestinal stem cells (Fig. 2b and Extended Data Fig. 5i). Consequently, older *caRANK*^*vil-Tg*^ mice exhibited markedly compromised proliferation of crypt cells (Extended Data Fig. 5f,g) and, as expected from diminished stem cell numbers, the villi were reduced in length and these mice exhibited decreased numbers of crypts and villi (Fig. 2c and Extended Data Fig. 5j–l). Phenocopying our observations on sustained RANK–RANKL activation in vitro, expression of a constitutive active form of RANK in the intestinal epithelium, results first in villous expansion followed by stem cell loss, altered villous structures and premature death of the mice.

**Extended Data Fig. 6:**
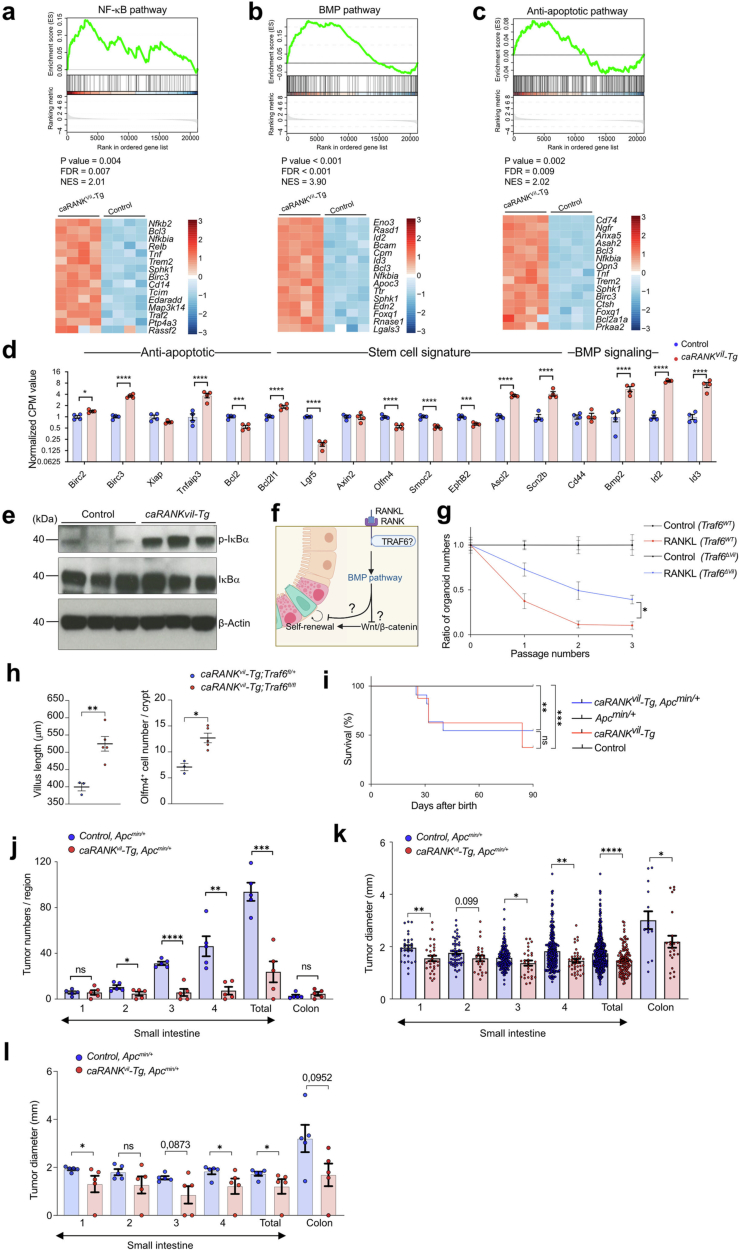
The key pathways for RANK-induced stem cell exhaustion in vivo. **a-c**, Alterations in the NF-κB, anti-apoptotic, and BMP pathways in jejunal organoids from *caRANK*^*vil*^*-Tg* mice (n = 4) compared to organoids from control mice (n = 4). Upper panels show GSEA enrichment plots. Bottom panels show heatmaps of the top 15 genes upregulated in *caRANK*^*vil*^*-Tg* organoids. Expression profiles of RANKL stimulated jejunal organoids were compared to non-stimulated (control) jejunal organoids cultured for the same time period. Total RNA was isolated from small intestinal organoids generated from two weeks old control and *caRANK*^*vil*^*-Tg* littermate (n = 4 for each group) and processed for RNA-seq. **d**, Differential gene expression analysis of RNA-seq data from mouse jejunal organoids derived from *caRANK*^*vil*^*-Tg* (n = 4) and control (n = 4) mice. Normalized CPM values of selected transcripts of anti-apoptotic genes, stem cell signature genes and BMP signalling genes are shown. **e**, Western blotting of phosphorylated IκB-α and total IκB-α in isolated intestinal epithelial cells from 3 wks old control (n = 3) and *caRANK*^*vil*^*-Tg* (n = 3) mice. β-Actin is shown as a loading control. For gel resource data, see Supplementary Fig. 1. **f**, Schematic outline of the proposed role of RANK/RANKL-induced stem cell proliferation and exhaustion. The figure was created with BioRender.com. **g**, Ratios of the numbers of organoids derived from *Traf6*^*WT*^ and *Traf6*^*ΔVil*^ mice after prolonged culture in the presence of rmRANKL (50 ng/ml). Numbers of organoids were counted at each passage. The ratio of organoid numbers in the RANKL *Traf6*^*WT*^ group was normalized to control *Traf6*^*WT*^ organoids, whereas the ratio of organoid numbers in the RANKL treated *Traf6*^*ΔVil*^ group was normalized to untreated control *Traf6*^*ΔVil*^ organoids. Data were combined from two independent experiments. n = 11 (*Traf6*^*WT*^), n = 11 (*Traf6*^*WT*^ + RANKL), n = 11 (*Traf6*^*ΔVil*^), n = 11 (*Traf6*^*ΔVil*^ + RANKL). **h**, Average length of villi and average number of OLFM4^+^ cells in each crypt per mouse from the indicated mice in the indicated mice described in Fig. 2d,e. **i**, Kaplan–Meier survival curve of control (*n* = 14), *caRANK*^*vil*^*-Tg* (*n* = 8), *Apc*^*min/+*^ (*n* = 13), and *caRANK*^*vil*^*-Tg*, *Apc*^*min/+*^ (*n* = 11) mice. **j**, Numbers of macroscopic adenomas and **k,l**, tumour diameters in the small intestine and colon of 4 months old control *Apc*^*min/+*^ (*n* = 5 mice) and *caRANK*^*vil*^*-Tg*, *Apc*^*min/+*^ (*n* = 5) mice. Small intestines were divided equally into 4 parts from the proximal (duodenum, labelled 1) to the distal (ileum, labelled 4) and adenomas assessed for each region. Total tumour numbers in *Apc*^*min/+*^ mice; *n* = 29 in region 1, *n* = 53 in region 2, *n* = 156 in region 3, *n* = 231 in region 4, *n* = 469 in total, *n* = 14 in colon, and in *caRANK*^*vil*^*-Tg*, *Apc*^*min/+*^ mice (*n* = 29/24/29/37/119/23). Each data point in **k** represents the measurements of the tumour diameter in individual tumours and in **l** represents the average measurement of the tumour diameters per mouse. Data are mean ± s.e.m. *P < 0.05; **P < 0.01; ***P < 0.001; ****P < 0.0001; ns, not significant. Enrichment-adjusted p-values (p-value), False Discovery Rates (FDR) and Normalized Enrichment Scores (NES) were calculated using two sided-fGSEA (**a-c**). Two-sided DESeq2 Wald tests, adjusted with the Benjamini–Hochberg procedure (**d**). One-way analysis of variance (ANOVA) with Tukey’s post hoc test (**g**); Two-tailed Mann–Whitney *U*-test (**h**,**j,l**); Kaplan–Meier survival curve with a log-rank test (**i**); Two-tailed Student’s t-test (**k**). Source Data

To further explore the underlying mechanism, we generated intestinal organoids from *caRANK*^*vil-Tg*^ mice. Constitutive activation of RANK resulted in enhanced organoid growth, yet again reduced numbers of proliferating cells in the buds and impaired maintenance of stem cells as determined by their passaging capacity (Extended Data Fig. 5m–o). Notably, as our constitutively active RANK mutant is still inducible by RANKL, addition of RANKL resulted in rapid growth arrest and even disintegration of all organoids (Extended Data Fig. 5p). Moreover, organoids from *caRANK*^*vil-Tg*^ mice were more resistant to radiation injury (Extended Data Fig. 5q). Bulk RNA-seq profiling showed that constitutive RANK activation resulted in upregulation of an anti-apoptotic program, NF-κB activation, and induction of *Bmp2* and its target genes *Id2* and *Id3*, whereas expression of defined stem cell markers such as *Lgr5*, *Olfm4*, *Smoc2* and *Ephb2* was downregulated (Extended Data Fig. 6a–d). Thus, expression of constitutively active RANK recapitulates results in RANKL-stimulated wild-type intestinal organoids.

We next assessed the in vivo functional relevance of NF-κB signalling, which was markedly deregulated after RANK activation. We observed substantial induction of phosphorylated IκB in intestinal epithelial cells isolated from *caRANK*^*vil-Tg*^ mice, indicative of activated NF-κB signalling (Extended Data Fig. 6e). Addition of the NF-κB inhibitor sc-514 rescued the RANK-driven BMP and anti-apoptotic gene expression phenotypes (Extended Data Fig. 2g), indicating that NF-κB acts a key signalling pathway downstream of RANK. One key molecular adapter that couples RANK to the canonical NF-κB activation is TRAF6 (ref. ^30^) (Extended Data Fig. 6f). We deleted *Traf6* in intestinal cells by crossing *Traf6*^*floxed*^ mice^31^ to *Villin1-cre* mice (hereafter, *Traf6*^*Δvil*^ mice). Stimulation of *Traf6*^*Δvil*^ intestinal organoids partially rescued the effects of RANK–RANKL activation on stem cell exhaustion compared with organoids from control littermates (Extended Data Fig. 6g). We crossed the *Traf6*^*Δvil*^ mice to *caRANK*^*vil-Tg*^ mice. Deletion of *Traf6* in intestinal epithelial cells enhanced the numbers of OLFM4^+^ stem cells and restored the decreased villus length observed in older *caRANK*^*vil-Tg*^ mice (Fig. 2d,e and Extended Data Fig. 6h). Thus, the effects of constitutively active RANK are partially driven by TRAF6 and NF-κB.

To determine whether constitutively active RANK regulates WNT-dependent aberrant stem cell expansion and adenoma initiation induced by loss of APC function, we crossed *caRANK*^*vil-Tg*^ mice to *Apc*^*min/+*^ mice. APC acts downstream of WNT and the *Apc*^*min*^ mutation drives aberrant WNT activation, resulting in uncontrolled proliferation and intestinal adenomatous polyposis in mice^32^. *Apc*^*min/+*^ mice exhibited premature death when RANK signalling was constitutively active in the *caRANK*^*vil-Tg*^*Apc*^*min/+*^ mice (Extended Data Fig. 6i). Importantly, whereas *Apc*^*min/+*^ mice developed hundreds of adenomas in the small intestine, the numbers and sizes of adenomas were reduced in the surviving *caRANK*^*vil-Tg*^*Apc*^*min/+*^ mice (Fig. 2f and Extended Data Fig. 6j–l). Notably, tumour organoids from *Apc*^*min/+*^ mice were still sensitive to the RANK–RANKL-driven exhaustion phenotype and were challenging to maintain for more than two passages in the presence of RANKL (Fig. 2g). Thus, in vivo constitutively active RANK suppresses the growth of *Apc*^*min*^ mutant intestinal stem cell tumours.

## RANK controls gut expansion in reproduction

**Fig. 3:**
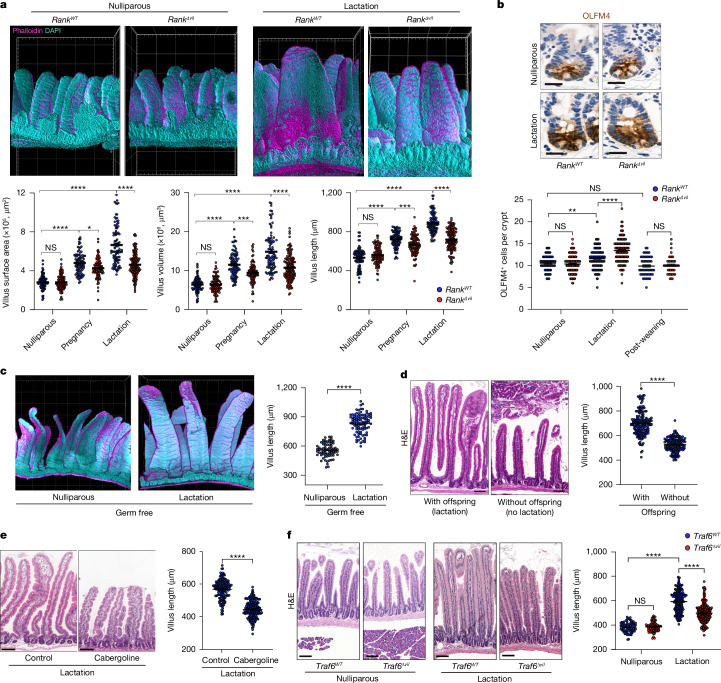
RANK–RANKL controls intestinal villus expansion in pregnancy and lactation. **a**, Representative 3D reconstruction of the upper small intestine from nulliparous and lactating (5 days after delivery, L5) *Rank*^*WT*^ and *Rank*^*Δvil*^ female mice (top). Grid spacing is 200 μm. Bottom, the small intestinal villus length, volume and surface areas in nulliparous *Rank*^*WT*^ (*n* = 5 mice, *n* = 88 villi), nulliparous *Rank*^*Δvil*^ (*n* = 5 mice, *n* = 97 villi), P18.5 *Rank*^*WT*^ (*n* = 6 mice, *n* = 89 villi), P18.5 *Rank*^*Δvil*^ (*n* = 6 mice, *n* = 93 villi), L5 *Rank*^*WT*^ (*n* = 5 mice, *n* = 91 villi) and L5 *Rank*^*Δvil*^ (*n* = 6 mice, *n* = 141 villi) mice. **b**, Representative OLFM4 immunostaining in the small intestines of the indicated mice (top). Scale bars, 20 μm. Bottom, quantification of OLFM4^+^ cells per crypt in nulliparous *Rank*^*WT*^ (*n* = 3 mice, *n* = 87 crypts), nulliparous *Rank*^*Δvil*^ (*n* = 4 mice, *n* = 130 crypts), L5 *Rank*^*WT*^ (*n* = 6 mice, *n* = 160 crypts) and L5 *Rank*^*Δvil*^ (*n* = 7 mice, *n* = 181 crypts) mice, and *Rank*^*WT*^ (*n* = 4 mice, *n* = 59 crypts) and *Rank*^*Δvil*^ (*n* = 5 mice, *n* = 44 crypts) mice 6 weeks after weaning of the offspring. **c**, Representative 3D images (left) and villi lengths of small intestines from age-matched nulliparous (*n* = 3 mice, *n* = 70 villi analysed) and lactating (*n* = 5 mice, *n* = 94 villi) germ-free C57BL6 mice (right). Grid spacing is 200 μm. **d**, Left, representative small intestinal sections (haematoxylin and eosin (H&E) staining) of L5 wild-type mice and wild-type mice whose offspring were removed on 1 day after delivery (no lactation). Scale bars, 100 μm. Right, quantification of villus length in L5 dams (with offspring) (*n* = 7 mice, *n* = 170 villi) and female mice with their offspring removed (without offspring) (*n* = 5 mice, *n* = 158 villi). **e**, Representative small intestinal sections (H&E staining) (left) and quantification of villi lengths (right) of L5 wild-type mice treated with DMSO or cabergoline (5 mg per kg) for 5 consecutive days starting on the day of delivery. Villi were assessed on day 5 after delivery. Scale bars, 100 μm. Right, quantification of villus length in mice treated with DMSO (*n* = 4 mice, *n* = 148 villi) and mice treated with cabergoline (*n* = 4 mice, *n* = 179 villi). **f**, Representative H&E-stained intestinal sections from nulliparous *Traf6*^*WT*^ and *Traf6*^*Δvil*^ mice, and L5 lactating *Traf6*^*WT*^ and *Traf6*^*Δvil*^ mice (left). Scale bars, 100 μm. Right, quantification of villous length in nulliparous *Traf6*^*WT*^ (*n* = 4 mice, *n* = 76 villi) and *Traf6*^*Δvil*^ (*n* = 5 mice, *n* = 73 villi) mice, and L5 *Traf6*^*WT*^ (*n* = 6 mice, *n* = 198 villi) and *Traf6*^*Δvil*^ (*n* = 5 mice, *n* = 226 villi) mice. Data are mean ± s.e.m. Each point represents the measurement of length, volume and surface area (**a** (bottom) and **c**–**f** (right)), and the number of OLFM4^+^ cells per crypt (**b**, bottom). Statistical analysis was performed using one-way ANOVA with Tukey’s post hoc test (**a**, **b** and **f**) and two-tailed Student’s *t*-tests (**c**–**e**); **P < 0.01, ***P < 0.001. Source Data

**Extended Data Fig. 7:**
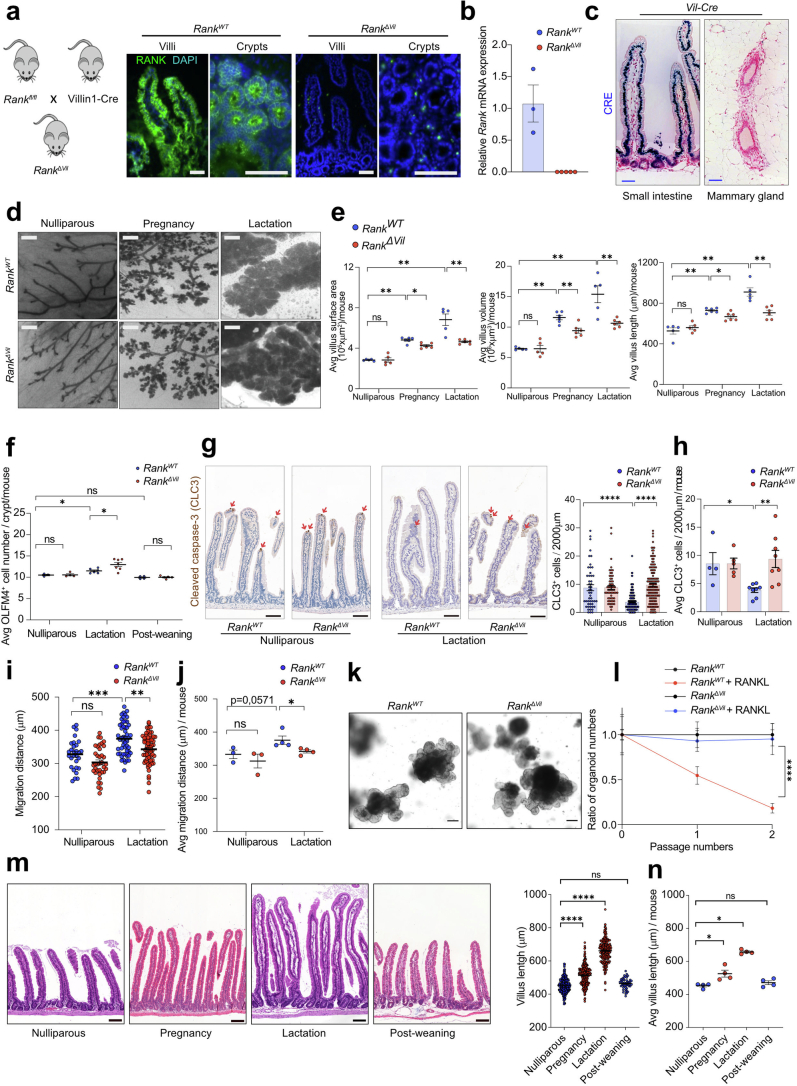
Cellular characterization of intestinal villi expansion in pregnancy and lactation. **a**, Mating strategy to generate intestinal epithelial cell specific *Rank* deleted mice (left) and anti-RANK immunostaining on intestinal cryo-sections from nulliparous control *Rank*^*WT*^ and *Rank*^*ΔVil*^ mice (right). Scale bars, 50 μm. **b**, Quantitative RT–PCR analysis of *Rank* mRNA expression in isolated small intestinal epithelial cells from nulliparous control *Rank*^*WT*^ (n = 3) and *Rank*^*ΔVil*^ (n = 5) littermates. **c**, Representative immunostaining to detect CRE expression (blue) in the small intestine (left panel) and mammary gland (right panel) of *Villin1-Cre* mice. Paraffin sections were counterstained with hematoxylin. Scale bars, 50 μm. **d**, Representative whole mount images (hematoxylin staining) of mammary glands from nulliparous *Rank*^*WT*^, nulliparous *Rank*^*ΔVil*^, pregnant *Rank*^*WT*^ (day 18.5 of pregnancy, P18.5), pregnant *Rank*^*ΔVil*^ females, lactating *Rank*^*WT*^ (5 days after delivery, L5) and lactating L5 *Rank*^*ΔVil*^ females. Scale bars, 500 μm. **e**, Small intestinal villi length, volume and surface areas in indicated mice. Each data point represents average villi length, volume and surface area per mouse in the indicated mice described in Fig. 3a. **f**, Quantification of the average number of OLFM4^+^ cells in each crypt per mouse in the indicated mice described in Fig.  3b. **g,h**, Cell death of villous epithelial cells from nulliparous *Rank*^*WT*^ and *Rank*^*ΔVil*^ females and age-matched lactating (L5) *Rank*^*WT*^ and *Rank*^*ΔVil*^ dams, as determined by immunostaining of cleaved caspase 3 (CLC3). Representative immunostaining. Scale bars, 100 μm (left in **g**). Quantification of CLC3 positive cells in villi per 2 mm of the intestine in nulliparous *Rank*^*WT*^ (n = 4 mice, n = 49 regions analysed) and *Rank*^*ΔVil*^ females (n = 5/59) and age-matched lactating (L5) *Rank*^*WT*^ (n = 8/162) and *Rank*^*ΔVil*^ dams (n = 8/135) (right in **g** and **h**). Each data point represents the numbers of CLC3 positive cells in villi per each 2 mm of the intestine (**g**) and average number per mouse (**h**). **i,j**, Quantification of the EdU-labelled intestinal epithelial cell migration along the crypt-villus axis in nulliparous *Rank*^*WT*^ (n = 3 mice, n = 29 crypt-villus axis analysed) and *Rank*^*ΔVil*^ females (n = 3/37) and age-matched lactating (L5) *Rank*^*WT*^ (n = 4/52) and *Rank*^*ΔVil*^ dams (n = 4/62). Tissues were harvested 24 h after EdU administration. Each data point represents the measurements of the migration distance in individual crypt-villus axis (**i**) and the average distance per mouse (**j**). **k**, Representative images of jejunal organoids generated from *Rank*^*WT*^ and *Rank*^*ΔVil*^ mice. Scale bars, 100 μm. **l**, Ratios of the number of jejunal organoids derived from *Rank*^*WT*^ and *Rank*^*ΔVil*^ mice after prolonged culture in the presence of rmRANKL (50 ng/ml). The number of organoids was counted at each passage. The ratio of organoid numbers in the *Rank*^*WT*^ + RANKL group was normalized to control *Rank*^*WT*^ organoids, whereas the ratio of organoid numbers in the *Rank*^*ΔVil*^ + RANKL group was normalized to untreated *Rank*^*ΔVil*^ organoids. Data are from two independent experiments. n = 6 (*Rank*^*WT*^), n = 6 (*Rank*^*WT*^ + RANKL), n = 6 (*Rank*^*ΔVil*^), n = 6 (*Rank*^*ΔVil*^ + RANKL). **m,n**, Representative hematoxylin and eosin (H&E) stained images of the upper small intestine (left in **m**) and quantification of upper small intestinal villus lengths (right in **m**, **n**) in age matched wild type nulliparous females (n = 4 mice, n = 310 villi analysed), pregnant females (P18.5) (n = 4/272), lactating dams (L5, 5 days after delivery) (n = 4/212) and females 6 weeks after weaning of the offspring (n = 4/49). Scale bars, 100 μm. Each data point represents the length of individual villi (**m**) and average length per mouse (**n**). Data are mean ± s.e.m. *P < 0.05; **P < 0.01; ***P < 0.001; ****P < 0.0001; ns, not significant. Two-tailed Mann–Whitney *U*-test (**e,f,h,j,n);** One-way analysis of variance (ANOVA) with Tukey’s post hoc test (**g,i,l,m**). Source Data

To assess the physiological role of RANK-driven villous expansion in intestinal homeostasis, we generated intestinal-epithelium-specific *Rank*-knockout mice (*Rank*^*Δvil*^ mice)^33^. Intestinal RANK expression was abolished in *Rank*^*Δvil*^ mice (Fig. 1a and Extended Data Figs. 1a and 7a,b). Notably, we did not observe Cre expression in the mammary tissue; moreover *Rank*^*Δvil*^ mice exhibited normal expansion of the mammary tissue in pregnancy and lactation (Extended Data Fig. 7c,d). Villous morphology was apparently not affected by *Rank* deletion in nulliparous *Rank*^*Δvil*^ mice (Fig. 3a and Extended Data Fig. 7e). The numbers of villous epithelial cells undergoing apoptosis and of OLFM4^+^ intestinal stem cells and stem cell divisions were also unchanged (Fig. 3b and Extended Data Fig. 7f–j). In vitro intestinal organoid formation was comparable between *Rank*^*Δvil*^ and *Rank* wild-type mice (*Rank*^*WT*^ mice), and organoids generated from *Rank*^*Δvil*^ mice could be passaged in the presence of RANKL (Extended Data Fig. 7k,l). Thus, the inactivation of RANK in intestinal epithelial cells does not disrupt intestinal stem cell homeostasis.

**Extended Data Fig. 8:**
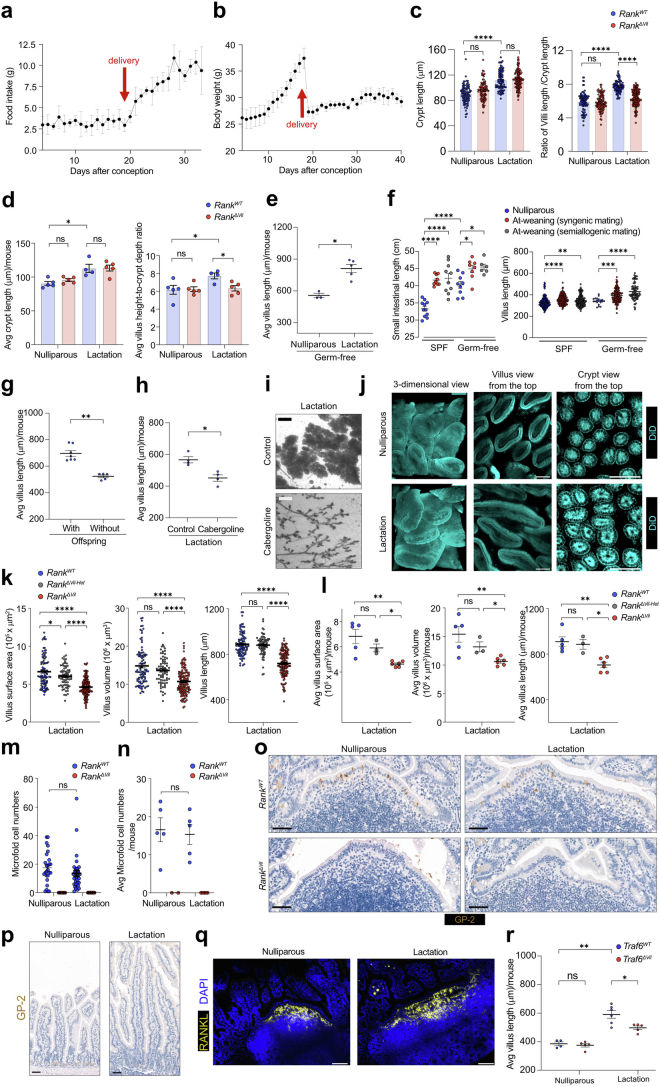
The detailed characterization of the intestine in reproduction. **a**, Daily food intake in wild type mice during pregnancy and lactation (*n* = 4). **b**, Body weights of wild type mice during pregnancy and lactation (*n* = 4). **c,d**, Crypt lengths (left) and villus height-to-crypt depth ratios (right) in nulliparous *Rank*^*WT*^ (*n* = 5 mice, *n* = 87 crypts, n = 88 villi analysed), nulliparous *Rank*^*ΔVil*^ (*n* = 5/87/97), L5 *Rank*^*WT*^ (n = 4/88/79) and L5 *Rank*^*ΔVil*^ (n = 5/116/134) females. Each data point represents the measurement of the crypt length and villus height/crypt depth ratio from individual villi (**c**) and the average value per mouse (**d**). These data were determined on images of the upper small intestine obtained by confocal microscopy. **e**, Average villus length per mouse from nulliparous and lactating germ-free C57BL6 mice in the indicated mice described in Fig. 3c. **f**, Quantification of small intestinal lengths (left) and small intestinal villus lengths (right) in age-matched wild-type nulliparous females in specific-pathogen free condition (SPF) (*n* = 11), females at weaning date mated with C57BL6/J (syngenic mating) in SPF (*n* = 10), females at weaning date mated with Balb/c (semiallogenic mating) in SPF (*n* = 9), nulliparous females in Germ-free condition (GF) (*n* = 9), females at weaning date mated with C57BL6/J (syngenic mating) in GF (*n* = 8), and females at weaning date mated with Balb/c (semiallogenic mating) in GF (*n* = 6). Each data point represents the measurement of the small intestinal length and villus length, and data are pooled from the indicated number of mice in each group. The number of measured villi; *n* = 240 (nulliparous mice, SPF), *n* = 200 (synergic mating, at weaning, SPF), *n* = 160 (semiallogenic mating, at weaning, SPF), *n* = 20 (nulliparous mice, Germ-free), *n* = 160 (synergic mating, at weaning, Germ-free), *n* = 80 (semiallogenic mating, at weaning, Germ-free). **g**, Average villus length per mouse in the indicated mice described in Fig. 3d. **h**, Average villus length per mouse from the indicated mice described in Fig. 3e. **i**, Representative whole mount images (hematoxylin staining) of mammary glands from lactating L5 females treated with DMSO or Cabergoline (5 mg/kg). Scale bars, 500 μm. **j**, Representative 3D images of upper small intestine in age matched nulliparous and lactating (L5) wild type females. DiD was used to reveal the morphology of the villus and crypt by labelling the cell membrane. Scale bars, 100 μm. **k,l**, Lengths, volumes and surface areas of villi measured in L5 *Rank*^*WT*^ (n = 5 mice, n = 91 villi analysed), L5 *Rank*^*ΔVil-Het*^ (n = 3/97) and L5 *Rankl*^*ΔVil*^ (n = 6/141) littermates. Each data point represents the length, volume, and surface area of individual villi (**k**) and average length per mouse. (**l**). **m,n**, Numbers of GP-2^+^ microfold (M) cells per Peyer’s patch from nulliparous *Rank*^*WT*^ (*n* = 5 mice, *n* = 26 Peyer’s patches analysed), nulliparous *Rank*^*ΔVil*^ (*n* = 2/9), L5 *Rank*^*WT*^ (*n* = 5/33) and L5 *Rank*^*ΔVil*^ (*n* = 5/18) females. GP-2^+^ M cells were determined on small intestinal sections using immunohistochemistry. Each data point represents the number of M cells per individual peyer patches (**m**) and average numer per mouse (**n**). **o**, Representative immunostaining of GP-2 in Peyer’s patch in the small intestine from nulliparous *Rank*^*WT*^ and *Rank*^*ΔVil*^ females and age-matched L5 *Rank*^*WT*^ and *Rank*^*ΔVil*^. Scale bars, 50 μm. **p**, Representative immunostaining of GP-2 in the small intestinal villi from nulliparous females and lactating (L5) females. Paraffin sections were counterstained with hematoxylin. Scale bars, 50 μm. **q**, Representative immunostaining to detect RANKL-expressing cells in the subepithelial dome in Peyer’s patch in nulliparous females and lactating (L5) females. DAPI (blue) was used to image nuclei. Scale bars, 100 μm. **r**, Average villus length per mouse in the indicated mice described in Fig. 3f. Data are shown as mean ± s.e.m. *P < 0.05; **P < 0.01; ***P < 0.001; ****P < 0.0001; ns, not significant. One-way analysis of variance (ANOVA) with Tukey’s post hoc test (**c,f,k,m**); Two-tailed Mann–Whitney *U*-test (**d,e,g,h,l,n,r)**. Source Data

We next examined the physiological function of the RANK–RANKL system in the intestine. In pregnancy and lactation, it has been reported that the intestine adapts to the increased nutrient demands in multiple species^34,35^. Although this is one of the most fundamental adaptions in all mammals and many non-mammalian species^4,5^, the molecular signals driving this expansion have never been identified. Moreover, in mammals, very few studies have followed up on initial reports describing larger intestines in pregnancy, with sometimes opposing findings^36–38^. We therefore repeated decade-old experiments in wild-type and *Rank*^*Δvil*^ mice. We observed markedly larger intestines in late-stage pregnancy and in particular in lactating wild-type mice (Fig. 3a and Extended Data Fig. 7e,m,n). Food intake was increased in pregnancy with a sharp surge at the onset of lactation (Extended Data Fig. 8a,b). We also detected crypt expansion in lactation and increased ratios of villus height to crypt depth (Extended Data Fig. 8c,d). We performed pregnancy experiments in germ-free mice at two different experimental sites and found that intestinal epithelial expansion still occurred in the absence of an intestinal microbiome (Fig. 3c and Extended Data Fig. 8e,f). Furthermore, it is independent of the antigenic properties of the fetus, as we observed the same changes in syngenic and semiallogenic breeding (Extended Data Fig. 8f). Moreover, removal of offspring at day 1 after birth to cease lactation resulted in smaller villi in the mothers (Fig. 3d and Extended Data Fig. 8g), suggesting that a prolactin feedback loop controls this expansion and involution. Treatment of lactating mice with cabergoline, a clinically used drug to reduce prolactin levels^39^, resulted in a reduction in the intestinal villi and, as a control, the mammary gland (Fig. 3e and Extended Data Fig. 8h,i). The morphological changes of the intestine in pregnancy/lactation is an intrinsic process that is primarily driven by pregnancy/lactation hormones that engage the RANK–RANKL pathway.

Using three-dimensional (3D) reconstructions, this expansion resulted in substantially longer villi and enhanced surface areas; the 3D villus volumes were increased in late-stage pregnancy (day 18.5 of pregnancy, P18, 5) and in lactating mice (Fig. 3a and Extended Data Fig. 7e). This enlargement also resulted in a more flattened geometry of the expanded villi, where the flat villi surface predominantly positions perpendicular to the flow of the intestinal content (Fig. 3a, Extended Data Figs. 7e and 8j and Supplementary Videos 1 and 2). In *Rank*^*Δvil*^ female mice, this intestinal villous expansion was impaired at late stages of pregnancy (P18.5) and lactation (lactation day 5 (L5)) compared with littermate and Cre controls (Fig. 3a, Extended Data Figs. 7e and 8k,l and Supplementary Videos 3 and 4). Notably, whereas loss of RANK expression had no apparent effect on the increased crypt depth in pregnancy, increased villus height-to-crypt depth ratios were RANK–RANKL dependent (Extended Data Fig. 8c,d). Previously it has been shown that RANK–RANKL is critical for the development of M cells in Peyer’s patches^18–20^, which were absent in our *Rank*^*Δvil*^ female mice; however, we did not observe increased numbers of M cells in lactation (L5) nor ectopic M cells in villi (Extended Data Fig. 8m–p). This lack of M cell expansion in pregnancy may be due to the fact that RANKL is highly expressed under the M cell domes of Peyer’s patches in nulliparous as well as lactating mice (Extended Data Fig. 8q). Deletion of *Traf6* in intestinal epithelial cells phenocopied the impaired epithelial expansion of lactating *Rank*^*Δvil*^ dams (Fig. 3f and Extended Data Fig. 8r).

**Extended Data Fig. 9:**
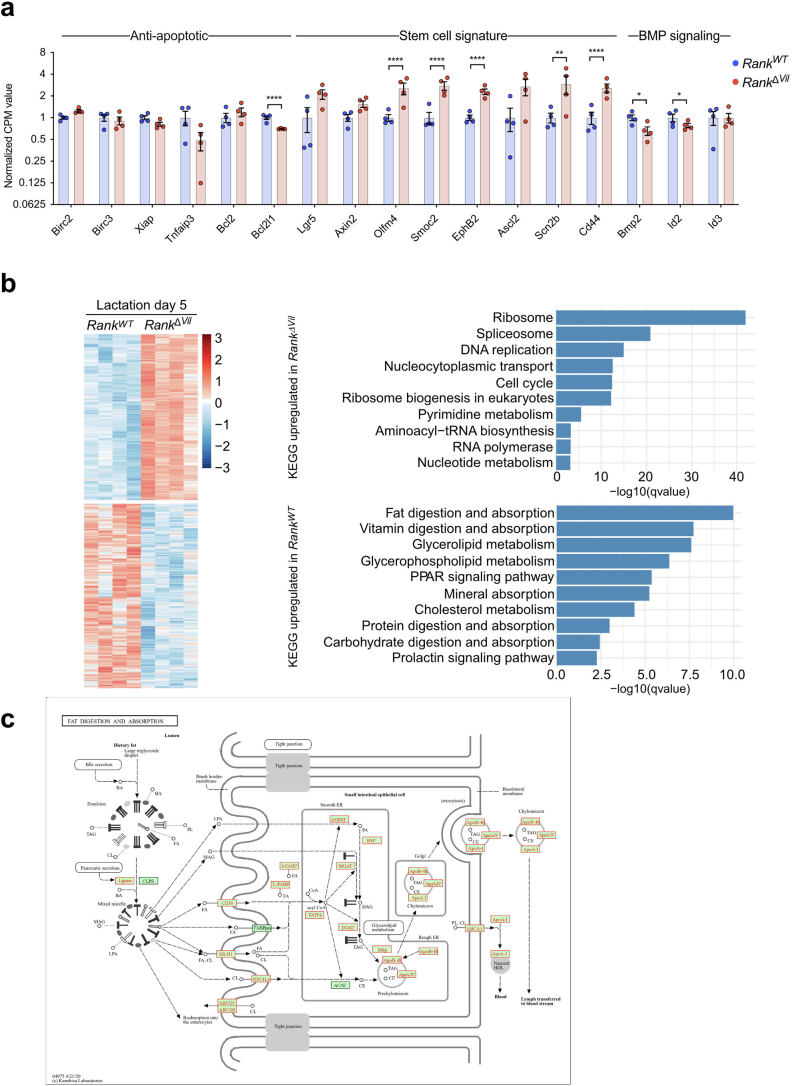
Bulk RNA-seq profiling of the intestine from lactating mice. **a**, Differential gene expression analysis of bulk RNA-seq data from intestinal epithelial cells isolated from L5 *Rank*^*WT*^ and L5 *Rank*^*ΔVil*^ mice (n = 4 for each group). Normalized CPM values of selected anti-apoptotic, stem cell signature and BMP signalling genes are shown. **b**, Heat maps of differentially expressed genes (left) and KEGG pathway analysis of differentially expressed genes (right) comparing intestinal epithelial cells from L5 *Rank*^*WT*^ and L5 *Rank*^*ΔVil*^ dams. The colour scale shows relative expression profiles. KEGG pathways overrepresented among up- and down-regulated differentially expressed genes (padj 0.05) are shown. **c**, KEGG pathway analysis for fat digestion and lipid absorption (mmu04975). The significantly upregulated genes in L5 lactating *Rank*^*WT*^ versus L5 *Rank*^*ΔVil*^ dams are highlighted in red. Genes that were not differentialy expressed between the groups are highlighted in green. Permission to use this figure was obtained from KEGG. Data are mean ± s.e.m. *P < 0.05; **P < 0.01; ****P < 0.0001; ns, not significant. Two-sided DESeq2 Wald tests, adjusted with the Benjamini–Hochberg procedure (**a**). Source Data

Gene expression profiling of intestinal epithelium at L5 showed upregulation of BMP signalling as well as induction of anti-apoptotic genes, dependent on RANK expression (Extended Data Fig. 9a), paralleling our findings in organoids and *caRANK*^*vil-Tg*^ mice. We therefore observed reduced cell death in the intestinal cells of lactating dams (Extended Data Fig. 7g,h). Gene expression profiling at L5 further showed RANK-dependent expression of the molecular machineries involved in the uptake of lipids, amino acids, sugar or vitamins as well as induction of the prolactin activation pathway (Extended Data Fig. 9b). Among the upregulated genes in lactation that were dependent on RANK were lipid receptors (such as *Cd36*, *Scarb1* (encoding SR-BI) or *Npc1l1*) and lipid transporters (including *Apob-48*, *Apoa1* and *Apoa4*) (Extended Data Fig. 9c). Thus, RANK promotes differentiation of functional absorptive enterocytes during lactation. Notably, during lactation, stem cell proliferation was slightly upregulated as determined by migration of EdU-labelled cells, dependent on RANK–RANKL (Extended Data Fig. 7i,j). Finally, we assessed expression of the intestinal stem cell marker OLFM4; we observed a small, albeit substantial, reduction in OLFM4^+^ stem cells in the upper small intestine of lactating (L5) controls compared with *Rank*^*Δvil*^ dams (Fig. 3b and Extended Data Fig. 7f), suggesting that lactating *Rank*^*Δvil*^ mice have more intestinal stem cells but they require RANK for proliferation and differentiation. These changes in OLFM^+^ stem cells were reversible and returned to non-pregnant baseline numbers after weaning (Fig. 3b and Extended Data Fig. 7f). Thus, RANK–RANKL, in part through TRAF6 signalling, constitutes a molecular pathway for intestinal epithelial adaptation during pregnancy and lactation.

## The source of RANKL for intestinal expansion

**Extended Data Fig. 10:**
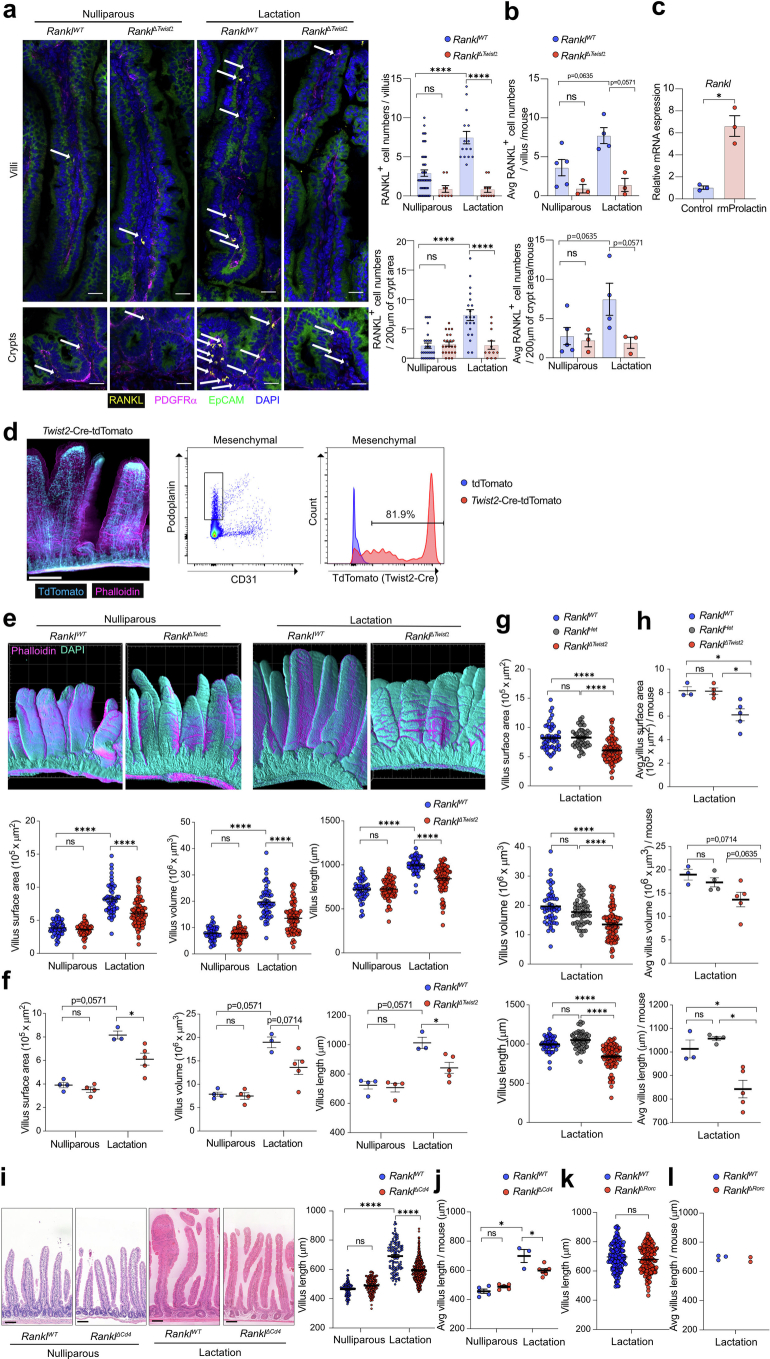
Parenchymal RANKL drives intestinal villous expansion. **a,b**, Representative immunostaining to detect RANKL expressing cells (arrows) in small intestinal cross-sections (Left in **a**). Sections were also stained for EpCAM to detect intestinal epithelial cells and PDGFRα to visualize subepithelial cells in villi. Scale bars, 25 μm. Quantification of RANKL positive cells per villus and 200 μm of crypt area from nulliparous *Rankl*^*WT*^ (n = 5 mice, n = 47 villi, n = 26 regions analysed) and *Rankl*^*ΔTwist2*^ (n = 3/9/23) mice and lactating (L5) *Rankl*^*WT*^ (n = 4/15/20) and *Rankl*^*ΔTwist2*^ (n = 3/11/12) mice (right in **a**, **b**). Each data point represents the measurements of numbers of RANKL^+^ cells per villi or 2 mm of crypt area (**a**) and the average number per mouse (**b**). **c**, Quantitative RT–PCR analyses to compare expression levels of *Rankl* in mesenchymal cultures derived from lamina propria. Data represent the relative expression of *Rankl* in recombinant mouse Prolactin (rmProlactin)-stimulated mesenchymal cells to control (no rmProlactin) mesenchymal cells (set at 1). rmProlactin stimulation was for 48 h. n = 3 (control), n = 3 (rmProlactin). **d**, Left panels, 3D reconstruction of small intestinal tissue from *Twist2*-Cre-tdTomato mice using confocal microscopy. Scale bars, 300 μm. Right panels, gating strategy to determine tdTomato-expression in mesenchymal cells from *Twist2*-Cre-tdTomato mice using FACS. **e,f**, Representative 3D reconstructions of small intestine from age-matched nulliparous *Rankl*^*WT*^ and *Rankl*^*ΔTwist2*^ and lactating (L5) *Rankl*^*WT*^ and *Rankl*^*ΔTwist2*^ dams. Grid spacing is 200 μm (upper in **e**). Villous length, volume and surface areas in nulliparous *Rankl*^*WT*^ (n = 4 mice, n = 49 villi analysed); nulliparous *Rankl*^*ΔTwist2*^ (n = 4/63); L5 *Rankl*^*WT*^ (n = 3/53); *Rankl*^*ΔTwist2*^ (n = 5/82) mice(lower in **e**, **f**). Each data point represents the measurements of length, volume and surface area of individual villi (**e**) and the average value per mouse. **g,h**, Lengths, volumes and surface areas of villi were measured in age matched L5 *Rankl*^*WT*^ (n = 3 mice, n = 53 villi analysed), L5 *Rankl*^*ΔTwist2*-*Het*^ (n = 4/50) and L5 *Rankl*^*ΔTwist2*^ (n = 5/82) littermates, using confocal microscopy and 3D tissue reconstructions. Each data point represents the measurements of length, volume and surface area of individual villi (**g**) and the average value per mouse(**h**). **i,j**, Small intestinal images from age-matched nulliparous and lactating (L5) *Rankl*^*WT*^ and *Rankl*^*ΔCd4*^ littermates. Left: Representative H&E stained intestinal sections. Scale bars, 100 μm (left in **i**). Quantification of villus length in nulliparous *Rankl*^*WT*^ (n = 5 mice, n = 89 villi analysed) and *Rankl*^*ΔCd4*^ (n = 5/135) mice and L5 *Rankl*^*WT*^ (n = 3/106) and *Rankl*^*ΔCd4*^ (n = 7/403) mice (right in **i**, **j**). Each data point represents the measurements of length of individual villi (**i**) and the average value per mouse(**j**). **k,l**, Quantification of villus length in age-matched L5 *Rankl*^*WT*^ (n = 3 mice, n = 149 villi analysed) and *Rankl*^*ΔRorc*^ (n = 2/148) mice, quantified from H&E cross sections. Each data point represents the measurements of length, volume and surface area of individual villi (**k**) and the average value per mouse(**l**). Data are mean ± s.e.m. *P < 0.05; ns, not significant. One-way analysis of variance (ANOVA) with Tukey’s post hoc test (**a,e,g,i**); Two-tailed Paired t-test (**c**); Two-tailed Mann–Whitney *U*-test (**b,f,h,j**); Two-tailed Student’s t-test (**k**). Source Data

**Extended Data Fig. 11:**
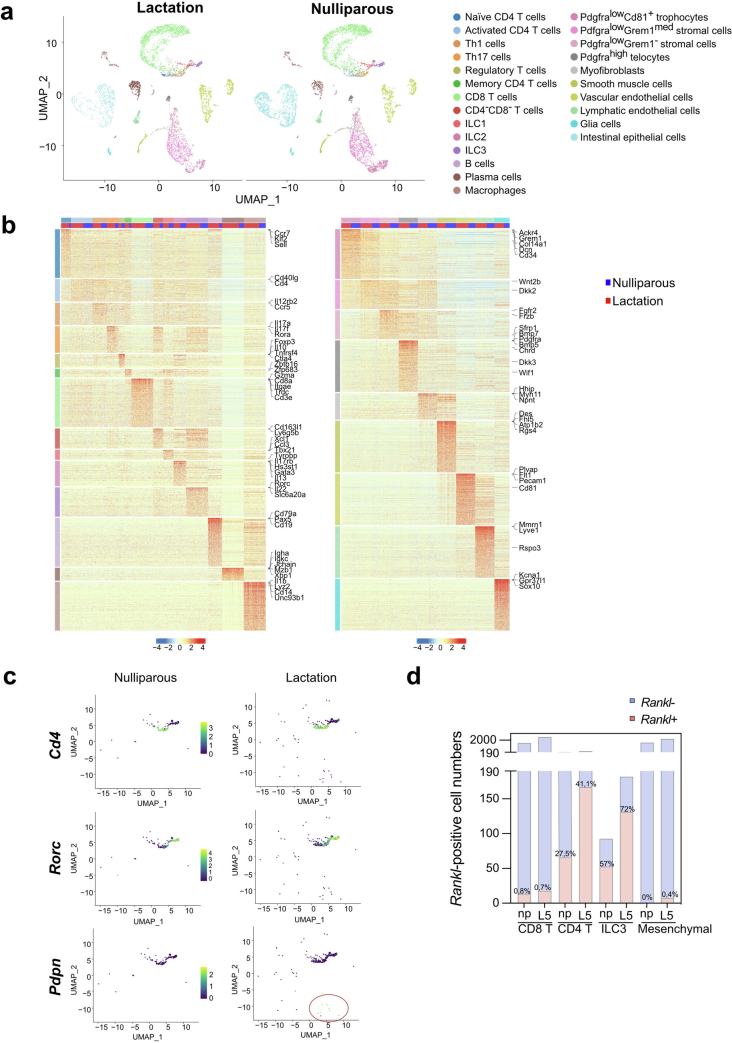
Single-cell RNA-seq profiling of lamina propria cells in nulliparous and lactating mice. **a**, Uniform manifold approximation and projection (UMAP) of 16,218 cells from nulliparous mice (6,822 cells) and lactating wild type mice (9,396 cells). Cells are colour-coded according to their cell-type annotation using unsupervised clustering. **b**, Heatmap representing scaled single-cell log-normalized expression of the top 300 differentially expressed genes for immune (left panel) and mesenchymal cell-types (right panel). Column annotation corresponds to cell-types (shown in the colour code as in panel a) and study conditions (nulliparous vs lactation L5). 200 cells per cell-type are represented after random downsampling. Genes of interest for each cell-type are indicated **c**, UMAP of *Rankl*-positive cells from age-matched nulliparous and L5 lactating littermate. Cell types are colour-coded as in a. **d**, Numbers of *Rankl*-positive and ratios of *Rankl*-positive CD4 T cells, CD8 T cells, ILC3 cells, and mesenchymal cells per total cell numbers from age-matched nulliparous and L5 lactating mice. Source Data

In intestinal epithelial cells, we did not detect any apparent changes in *Rank* expression between nulliparous and lactating females, indicating that the system is controlled by RANKL levels. When we assessed RANKL in intestinal epithelial cells in lactating dams, there was no measurable RANKL protein. However, in lactating dams, we observed a trend towards increased RANKL-expressing cells in the lamina propria of the small intestine (Extended Data Fig. 10a,b). When we treated ex vivo cultured lamina propria cells with prolactin, we observed induction of RANKL expression (Extended Data Fig. 10c). To identify the RANKL expressing cell type(s), we performed scRNA-seq analysis of mouse lamina propria cells; in lactating mice, *Rankl* expression was observed in T cells, innate ILC2 and ILC3 cells, and a small fraction of mesenchymal cells (Extended Data Fig. 11a–d). Recently, it has been shown that RANKL is expressed in parenchymal *Twist2*-expressing cells^19^. When we deleted *Rankl* using the *Twist2-cre* mouse line, RANKL^+^ cells in the lamina propria of lactating females were only sparsely present in lactating *Rankl*^*ΔTwist2*^ dams (Extended Data Fig. 10a,b,d). Importantly, in *Rankl*^*ΔTwist2*^ dams, villous expansion was impaired compared with in the control mice (Extended Data Fig. 10e–h). Similarly, deletion of *Rankl* in T cells using *Cd4-cre*^40^ (*Rankl*^*ΔCd4*^ mice) resulted in impaired villous expansion in lactating *Rankl*^*ΔCd4*^ dams (Extended Data Fig. 10i,j). By contrast, when we crossed *Rankl*^*floxed*^ mice to *Rorgt-cre* mice to delete RANKL in innate ILC3 cells *(Rankl*^*ΔRorc*^), we did not observe an effect on intestinal epithelial expansion in lactation (Extended Data Fig. 10k,l). Not excluding other cell types or the contribution of soluble RANKL, these data show that RANKL expression is induced in gut lamina propria cells during pregnancy/lactation and indicate that *Twist2-cre* expressing mesenchymal cells and T cells are local sources for RANKL, which then activates RANK signalling on intestinal epithelial cells to drive adaptive villous expansion.

## Transgenerational effects

**Fig. 4:**
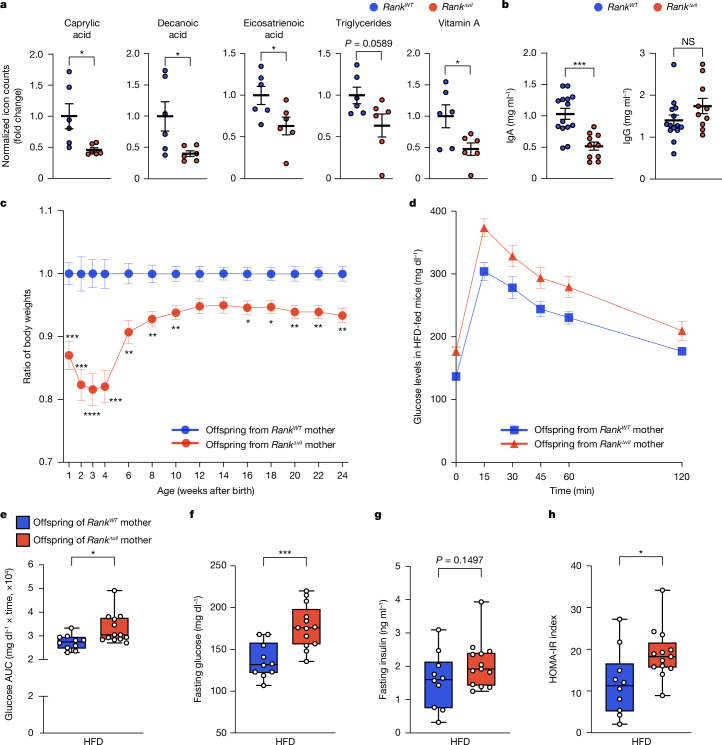
Transgenerational effects in the offspring of *Rank*^*Δvil*^ dams. **a**, The levels of free fatty acids, triglycerides and vitamin A in the milk of lactating (L8) *Rank*^*WT*^ and *Rank*^*Δvil*^ dams (*n* = 6 for each group) were measured using mass spectrometry. **b**, IgA and IgG concentrations were measured using an enzyme-linked immunosorbent assay (ELISA) analysis of the milk collected at L8 from *Rank*^*WT*^ and *Rank*^*Δvil*^ dams (*n* = 14 and 10 mice, respectively). **c**, The body weights of each offspring (*n* = 20) from *Rank*^*Δvil*^ mothers were normalized to the average body weight of the offspring (*n* = 23) born to control *Rank*^*WT*^ mothers (set at 1) at all of the indicated ages. **d**–**h**, Glucose tolerance analysis and insulin levels in male (*n* = 10 and 13 mice, respectively) offspring of *Rank*^*WT*^ and *Rank*^*Δvil*^ dams that were fed a HFD for 21 weeks. The oral glucose-tolerance test (**d**), the corresponding area under the curve (AUC) (**e**), the fasting glucose plasma levels (**f**), the fasting insulin plasma levels (**g**) and the homeostasis model assessment of insulin resistance (HOMA-IR) index (**h**) are shown. Data in **e**–**h** are shown as box and whisker plots; the plots show the median (middle line), interquartile range (box) and minimum to maximum values (whiskers) throughout. For **a**–**d**, data are mean ± s.e.m. Statistical analysis was performed using two-tailed Student’s *t*-tests (**a**, **b** and **e**–**h**) and two-way ANOVA with Šidák’s multiple-comparison correction (**c**). Source Data

**Extended Data Fig. 12:**
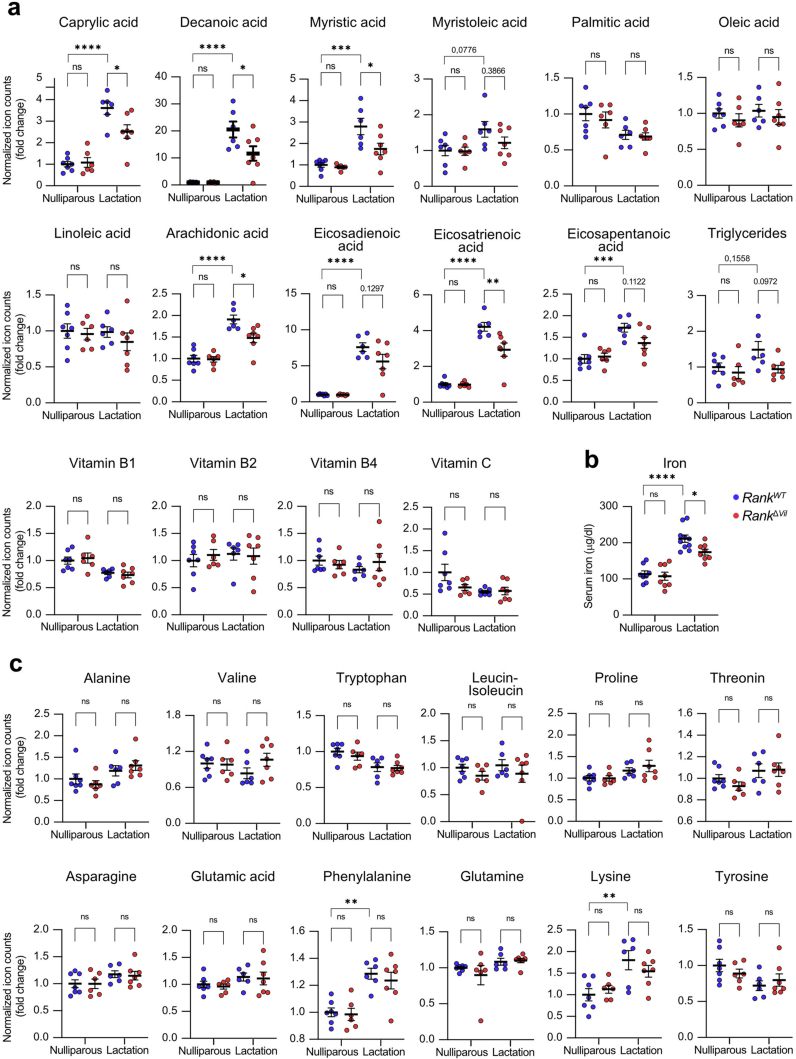
Metabolomic profiling of serum from nulliparous and lactating mice. **a,c**, Levels of the indicated free fatty acids, triglycerides, and Vitamins (**a**) and free amino acids (**c**) in the serum from nulliparous *Rank*^*WT*^ (n = 7) and *Rank*^*ΔVil*^ (n = 6) females and lactating (L8) *Rank*^*WT*^ (n = 6) and *Rank*^*ΔVil*^ (n = 7) dams, measured by mass spectrometry. **b**, Levels of the indicated iron in the serum from nulliparous *Rank*^*WT*^ (n = 8) and *Rank*^*ΔVil*^ (n = 8) females and lactating (L8) *Rank*^*WT*^ (n = 10) and *Rank*^*ΔVil*^ (n = 8) dams, measured by a *photometric colour test*. Dots represent relative levels of individual data points normalized to nulliparous control females. Data are shown as mean ± s.e.m. *P < 0.05; **P < 0.01; ns, not significant. One-way analysis of variance (ANOVA) with Tukey’s post hoc test (**a,b,c**). Source Data

**Extended Data Fig. 13:**
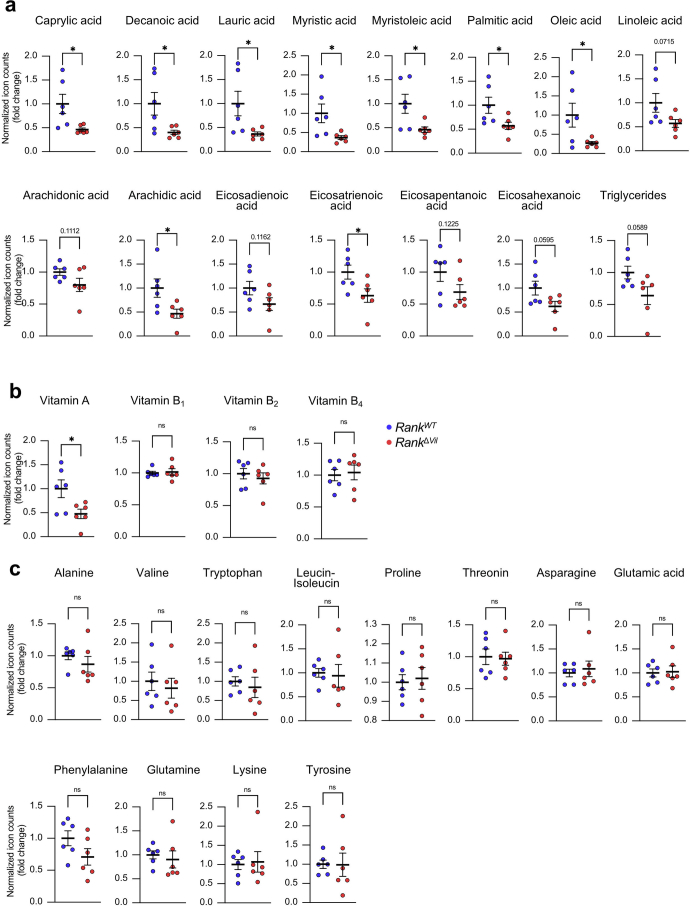
Metabolomic profiling of milk from nulliparous and lactating mice. **a-c**, Levels of free fatty acids and triglycerides (**a**), Vitamins (**b**) and free amino acids (**c**) in the milk from lactating (L8) *Rank*^*WT*^ and *Rank*^*ΔVil*^ dams (n = 6 for each group), measured by mass spectrometry. Collection of milk is described in Methods. Of note, some of the data are the same as shown in Fig. 5a. Dots represent relative levels of individual data points normalized to *Rank*^*WT*^ control females. Data are mean ± s.e.m. *P < 0.05; ns, not significant. Two-tailed Student’s t-test. Source Data

Adaptation of the intestinal epithelium in pregnancy has been proposed to allow for enhanced nutritional demands in pregnancy and lactation^34,35,41^. In the intestines of lactating female mice, we observed RANK-dependent expression of molecular machines involved in the uptake of lipids, amino acids, sugar or vitamins (Extended Data Fig. 9b). Consequently, in serum of *Rank*^*WT*^ dams, we detected an increase in defined lipids as well as iron compared with in nulliparous females; this increase in lactating dams was in part dependent on RANK expression in the gut epithelium (Extended Data Fig. 12a,b). Free amino acids in the serum were largely unchanged (Extended Data Fig. 12c). We next collected the milk of lactating *Rank*^*Δvil*^ mothers. Compared with the controls, the milk of *Rank*^*Δvil*^ dams contained reduced levels of multiple fatty acids, triglycerides and lipid soluble vitamin A, whereas amino acids or the hydrophilic vitamins B1, B2 and B4 appeared normal (Fig. 4a and Extended Data Fig. 13a–c). Importantly, we also observed a strong reduction in mucosal IgA, but not IgG, in the milk of lactating *Rank*^*Δvil*^ female mice (Fig. 4b). Ablation of RANK in the intestinal epithelium of dams results in metabolic and immunological alterations in the milk of the lactating mother.

**Extended Data Fig. 14:**
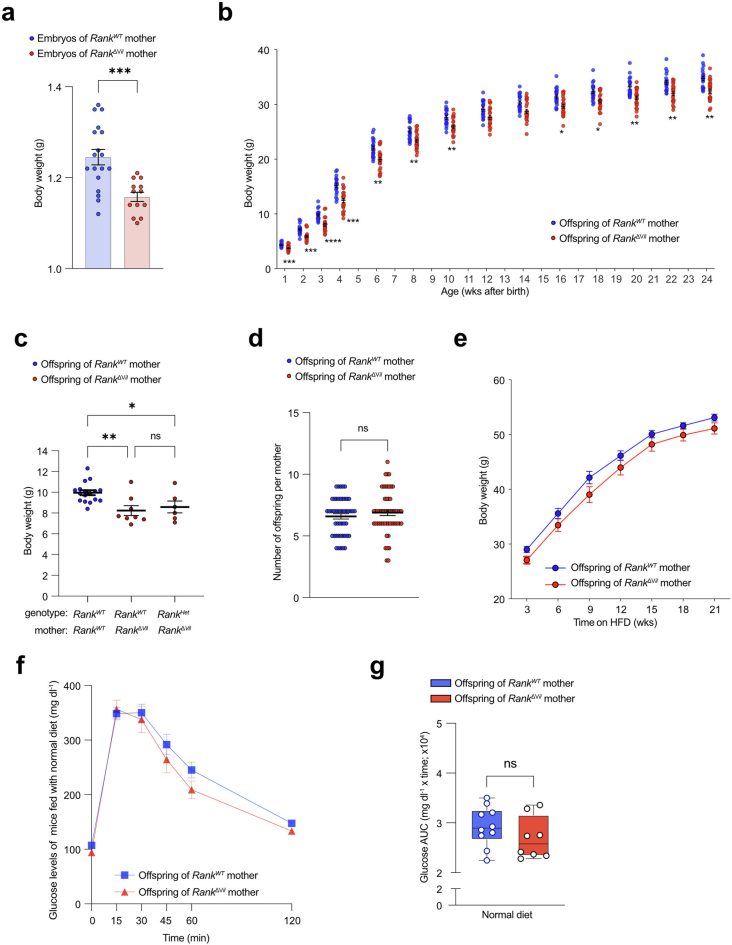
Transgenerational effects in the offspring of *Rank*^*Δvil*^ mothers. **a**, Body weights of E18.5 male embryos of *Rank*^*WT*^ (n = 18) and *Rank*^*ΔVil*^ (n = 13) females fed normal chow. **b**, Body weights of male offspring born to *Rank*^*WT*^ (n = 20) and *Rank*^*ΔVil*^ (n = 23) dams fed normal chow. Mice were monitored from wk 1 to wk 24^th^ after birth. Each data point represents one offspring mouse. **c**, Body weights of 3 wks old *Rank*^*WT*^ and *Rank*^*HET*^ offspring born to *Rank*^*WT*^ and *Rank*^*ΔVil*^ dams. All pregnancies were syngeneic crosses with C57BL/6J males, thus the offspring of *Rank*^*ΔVil*^ dams were either *Rank*^*WT*^ (*Rank*^*flox/WT*^*Vil*^*cre−*^) (n = 8) or *Rank*^*Het*^ (*Rank*^*flox/WT*^*Vil*^*cre+*^) (n = 6); both of these groups exhibit reduced body weights as compared to *Rank*^*WT*^(*Rank*^*flox/WT*^*Vil*^*cre−*^) offspring (n = 17) born to *Rank*^*WT*^ dams. Dots represent individual mice. **d**, Numbers of offspring from *Rank*^*WT*^ (n = 48) and *Rank*^*ΔVil*^ (n = 54) dams. **e**, Body weights of male offspring born to *Rank*^*WT*^ (n = 18) and *Rank*^*ΔVil*^ (n = 16) dams fed high fat diet (HFD). **f**,**g**, Oral glucose-tolerance test (**f**) and corresponding area under the curve (AUC) (**g**) in male (*n* = 10/8) offspring of *Rank*^*WT*^ and *Rank*^*ΔVil*^ dams, fed normal chow for 25 wks. Box-and-whisker plots in **g** show the median (middle lines), interquartile range (boxes), and minimum to maximum values (whiskers) throughout. Data are mean ± s.e.m. *P < 0.05; **P < 0.01; ***P < 0.001; ****P < 0.0001; ns, not significant. Two-tailed Student’s t-test (**a,d,g**); Two-way ANOVA with Sidak’s multiple comparison (**b**); One-way analysis of variance (ANOVA) with Tukey’s post hoc test (**c**). Source Data

In humans, children born to mothers with reduced nutrient intake in pregnancy, as observed in the Dutch famine of 1944, can experience long-term health complications, such as proclivity to insulin intolerance^42^. We therefore followed the fate of pups born to *Rank*^*Δvil*^ mothers. At embryonic day 18.5, the fetuses of *Rank*^*Δvil*^ dams already exhibited substantially decreased body weights compared with embryos from *Rank*^*WT*^ dams (Extended Data Fig. 14a). After birth, the pups born to *Rank*^*Δvil*^ dams continued to exhibit reduced weights, in particular during the nursing period, and remained smaller throughout life (Fig. 4c and Extended Data Fig. 14b,c). The numbers of offspring were comparable among *Rank*^*WT*^ and *Rank*^*Δvil*^ mothers (Extended Data Fig. 14d). When we exposed offspring born to *Rank*^*Δvil*^ and *Rank*^*WT*^ mothers to a high-fat diet (HFD), both groups gained weight, although the offspring of *Rank*^*Δvil*^ mothers exhibited lower absolute weights throughout the observation period (Extended Data Fig. 14e). Importantly, we observed impaired glucose tolerance and increased fasting glucose in the offspring of *Rank*^*Δvil*^ mothers on the HFD (Fig. 4d–f). Calculation of the HOMA-IR index resistance confirmed pronounced insulin resistance in the offspring of *Rank*^*Δvil*^ female mice (Fig. 4g,h). Glucose metabolism appeared unaffected in the offspring of *Rank*^*Δvil*^ dams that were fed a normal chow diet (Extended Data Fig. 14f,g). Thus, RANK-dependent villous expansion in the mothers is required for efficient nourishment of their offspring and results in transgenerational glucose intolerance under metabolic stress.

## RANK controls human intestinal stem cells

**Fig. 5:**
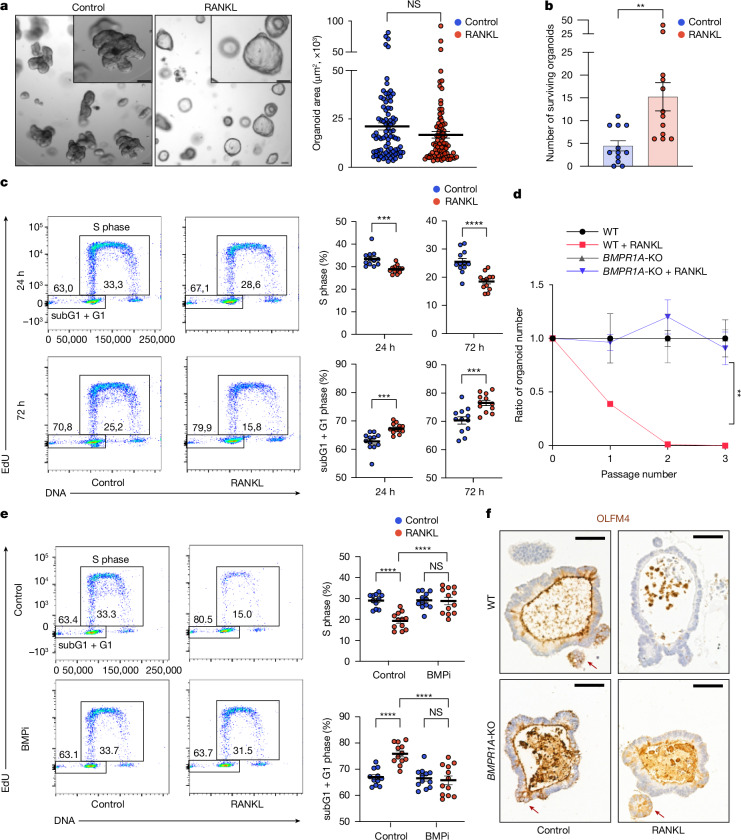
RANK–RANKL controls stem cells in human intestinal organoids. **a**, Human duodenal organoids were cultured without (control) and with recombinant human RANKL (rhRANKL; 500 ng ml^−1^) for 2 days. Left, representative images. Scale bars, 100 μm. Right, quantification of organoid sizes. *n* = 83 (control) and *n* = 83 (rhRANKL). Data were combined from three independent experiments. **b**, Organoids were cultured with or without rhRANKL (500 ng ml^−1^) for 2 days and then irradiated and cultured for additional 7 days. Data show the number of surviving organoids combined from three independent experiments. *n* = 12 (control) and *n* = 12 (rhRANKL). **c**, Cell cycle analyses of RANK-stimulated human duodenal organoids. Left, representative fluorescence-activated cell sorting (FACS) plots of the EdU cell cycle analysis assessed at 24 h and 72 h of rhRANKL stimulation. Right, quantification of S phase and subG1 + G1 entry. Data were combined from two independent experiments. *n* = 12 for each condition. **d**, The ratio of the numbers of wild-type (WT) and *BMPR1A*-knockout (*BMPR1A*-KO) human small intestinal organoids in the absence or presence of rhRANKL (500 ng ml^−1^). The ratio of organoid numbers in the WT + RANKL group was normalized to untreated WT organoids; and the ratio of organoid numbers in the *BMPR1A-*KO + RANKL organoids was normalized to the untreated *BMPR1A-*KO organoids. *n* = 3 for each condition. **e**, Cell cycle analyses of RANK-stimulated duodenal organoids in the absence (control) and presence of the BMPi LDN193189 (1.6 μM). Left, representative FACS plots depicting EdU cell cycle analysis after 72 h rhRANKL stimulation with or without BMPi. Right, quantification of S phase and sbG1 + G1 entry. *n* = 11 (control), *n* = 12 (control + RANKL), *n* = 12 (BMPi) and *n* = 12 (BMPi + RANKL). Data were combined from two independent experiments. **f**, Representative images of OLFM4^+^ stem cells in WT and *BMPR1A*-KO human small intestinal organoids with or without rhRANKL (500 ng ml^−1^). Scale bars, 50 μm. Dots represent individual datapoints. Data are mean ± s.e.m. Statistical analysis was performed using two-tailed Student’s *t*-tests (**a**–**c**) and one-way ANOVA with Tukey’s post hoc test (**d** and **e**). Further details on statistics and reproducibility are provided in the Methods. Source Data

**Extended Data Fig. 15:**
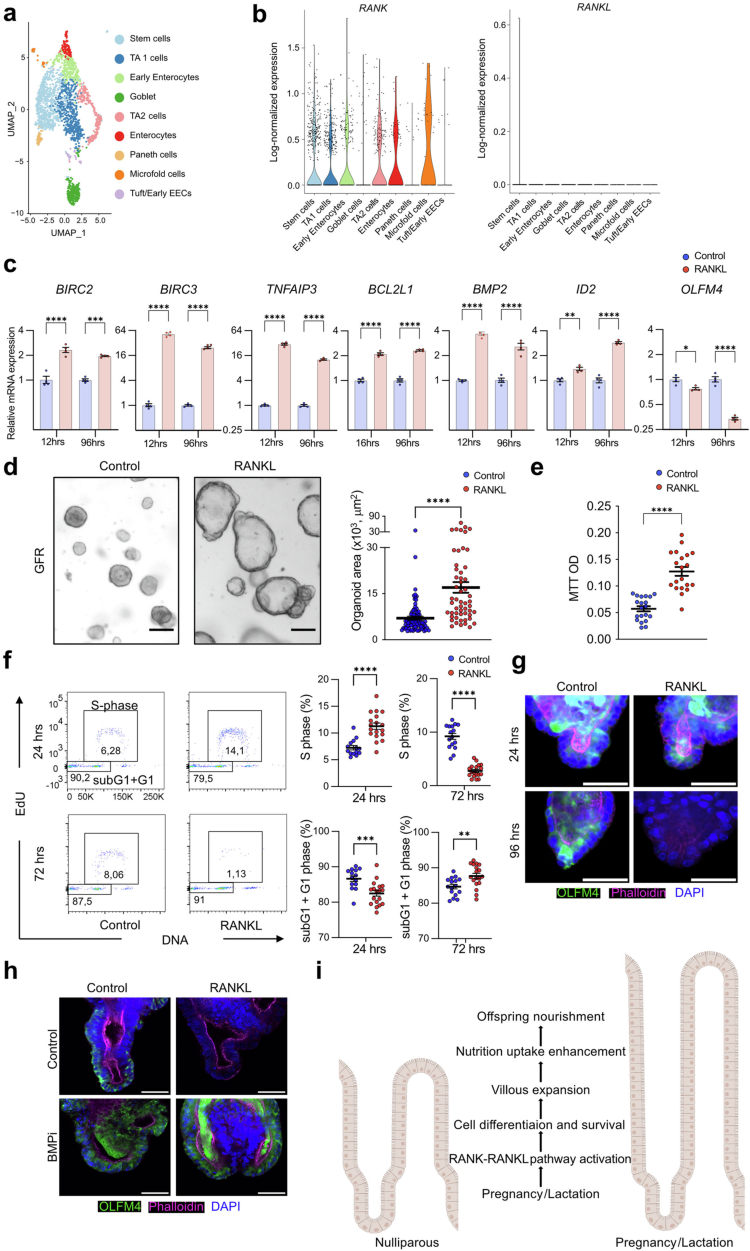
Characterization of stem cells in RANK–RANKL-stimulated human intestinal organoids. **a**, Uniform manifold approximation and projection (UMAP) of 2,265 human intestinal epithelial cells. Data were taken from Fujii et al. ^43^. Cells are colour coded according to epithelial cell-type annotation based on unsupervised clustering. **b**, Violin plots show single cell log-normalized expression of *RANK* and *RANKL* in each intestinal cell-type. Each dot represents an individual cell. **c**, Quantitative RT–PCR analyses to compare expression levels of anti-apoptotic genes, stem cell signature genes, and BMP signalling genes in human duodenal organoids. Data represent the relative expression of the indicated genes in rhRANKL (500 ng/ml) stimulated duodenal organoids compared to control (no RANKL) organoids (set at 1). rhRANKL stimulation was for 12 and 96 h. n = 4 (control), n = 4 (RANKL). **d**, Representative images (left) and quantified areas (right) of human duodenal organoids cultured for 2 days without (control; n = 83) and with recombinant human RANKL (rhRANKL; 500 ng/ml; n = 54) in growth-factor-reduced medium (GFR medium) lacking EGF, IGF-1, and FGF-2. Scale bars, 100 μm. Each dot represents an organoid, assessed in three independent experiments. **e**, RANKL-induced proliferation of duodenal organoids. Organoids were left untreated (control) or treated with rhRANKL (500 ng/ml) and proliferation determined using the MTT assay. Each plot represents an MTT OD, pooled from two independent experiments. n = 20 (control), n = 20 (RANKL). **f**, Representative cell cycle FACS plots (left) and quantification of S-phase and subG1 + G1 entry (right) assessing EdU labelled human duodenal organoids cultured in GFR medium and stimulated with rhRANKL for 24 and 72 h. Dots represent individual organoids, assessed in three independent experiments. *n* = 14 (control, 24hrs), *n* = 16 (control, 72hrs), *n* = 18 (rmRANKL, 24hrs), *n* = 18 (rmRANKL, 72hrs). **g**, Representative images of anti-OLFM4 immunostaining (green) of human duodenal organoids cultured in GFR medium in the absence (control) and presence of rhRANKL (500 ng/ml) for 24 and 96 h. Organoids were counterstained with DAPI (blue) to detect nuclei and Phalloidin (magenta) to detect filamentous actin. Scale bars, 50 μm. **h**, Representative images of OLFM4^+^ stem cells in human intestinal organoids cultured in the presence of rhRANKL (500 ng/ml) without (control, DMSO solvent) or with the BMP inhibitor (BMPi) LDN193189 (1.6μM) for 96 hrs. Organoids were stained with DAPI (blue) to detect nuclei and Phalloidin to detect filamentous actin. Scale bars, 50 μm. **i**, Proposed function of RANK–RANKL in the small intestine during pregnancy and lactation. During pregnancy and lactation, RANK–RANKL signalling promotes intestinal stem cell proliferation and differentiation as well as intestinal cell survival, ultimately resulting in massive villous expansion. Villous expansion facilitates nutritional uptake, which is important for nourishment of offspring as well as their transgenerational metabolic health. The figure was created with BioRender.com. Data are mean ± s.e.m. **P < 0.01; ***P < 0.001; ****P < 0.0001; ns, not significant. Two-tailed Student’s t-test (**c-f)**. More details on statistics and reproducibility can be found in the Methods. Source Data

Data mining^43^ in humans showed that, similar to mice, *RANK*, but not *RANKL*, is expressed on stem cells, TA cells and absorptive enterocytes (Extended Data Fig. 15a,b). We therefore examined whether RANK-mediated intestinal epithelial expansion also occurs in humans. We generated human duodenal organoids from a healthy female donor and RANKL stimulation indeed triggered morphological changes (Fig. 5a). RANKL stimulation of the human duodenal organoids again induced anti-apoptosis genes and the BMP pathway (Extended Data Fig. 15c). Consistent with the induction of an anti-apoptotic program, RANKL treatment promoted cell survival after irradiation of these human intestinal organoids (Fig. 5b).

RANK stimulation also affected cell division (Fig. 5c) and abrogated passaging of human duodenal organoids (Fig. 5d). Notably, when cultured under suboptimal growth conditions, RANKL initially triggered enhanced proliferation, followed by growth arrest and stem cell exhaustion within 3 days of culture (Extended Data Fig. 15d–g). OLFM4 immunostaining revealed a reduced number of intestinal stem cells in RANKL-treated human organoids (Extended Data Fig. 15g,h). We were able to rescue RANK-induced reduction of OLFM4^+^ stem cells and the proliferation defect using the BMP receptor-blocker LDN-193189 (BMPi) (Fig. 5e and Extended Data Fig. 15h). To validate the role of BMP signalling in RANK-induced stem cell exhaustion, we exposed *BMPR1A-*knockout^44^ human intestinal organoids to RANKL; genetic inhibition of the BMP signalling pathway completely rescued RANK-driven stem cell exhaustion (Fig. 5d,f). Thus, our observations in mice hold true in humans: RANK–RANKL drives growth of intestinal organoids and, at the same time, controls the self-renewal ability of intestinal stem cells through BMP signalling.

## Discussion

How mothers adapt to the demands of pregnancy remains a central question of evolution and human health. During pregnancy and lactation, hormone dynamics affect several organs to control and change their morphologies and functions, critically required for the health of the mother and the development of the offspring^2,3^. Our data reveal that, during pregnancy and lactation, RANKL is induced in parenchymal T cells and mesenchymal cells in the intestine. Whether membrane-bound and/or soluble RANKL from other cell types also contributes to the intestinal phenotype needs to be established. RANKL activates its receptor RANK on intestinal stem cells and differentiated enterocytes. RANK activation induces survival of the intestinal epithelial cells, in part through TRAF6 coupling to the NF-κB pathway, and through BMP signalling critically controls stem cell differentiation and the self-renewal capacity of intestinal stem cells, both of which appear to contribute to the expansion of small intestinal epithelial cells in pregnancy and lactation (Extended Data Fig. 15i). A potential cross-talk between BMP and apoptotic pathways on intestinal stem cells cannot be excluded. Furthermore, RANK is expressed on multiple types of intestinal epithelial cells and its role in these cells needs to be investigated.

In mouse and human organoids and, importantly, in pregnancy in mice, RANK stimulation triggers induction of the BMP2 pathway that suppresses self-renewal of intestinal stem cells and promotes differentiation^24,25^. In vivo constitutive RANK activation also results in the expansion of intestinal villi followed by stem cell exhaustion, villus shrinkage and death of about 50% of these mice. Notably, chronic activation of the RANK–RANKL system in *caRANK*^*vil-Tg*^ mice abolished WNT-driven intestinal tumorigenesis when crossed to an *Apc*^*min/+*^ background. Intestinal tumour organoids from *Apc*^*min/+*^ mice were still sensitive to RANKL-triggered stem cell exhaustion; whether this could be exploited for future intestinal cancer treatment needs to be explored. Importantly, whereas permanent induction of RANK signalling drives stem cell exhaustion in vivo, RANK-mediated changes in stem cells and villus expansion in pregnancy/lactation are reversible, regulated by the prolactin axis, a known and very strong inducer of RANKL expression^12^. Whether such reversibility is controlled by the duration of the RANK stimulus in vivo or might be regulated by additional signals needs to be determined. As we observed effects of RANK stimulation in both male and female mouse gut organoids, and male human organoids have not been tested yet, it might be interesting to examine whether this system controls gut remodelling only in female pregnancy or whether it also affects intestinal homeostasis in male individuals.

Multiple maternal tissues change during and after pregnancy to maintain the health of the mother and to facilitate the development of the fetus and nurturing of babies^1–3^. Such changes also occur in non-mammalian species making it a universal hallmark of evolutionary fitness^4^. Mothers need to respond to the increased nutritional demand in pregnancy and, in the case of mammals, lactation^45^. Although such nutritional demands are fundamental for the short- and long-term health of mothers and their offspring, little was known about the adaption of the mammalian intestine. Our results show that, in pregnancy and lactation, RANK–RANKL controls intestinal stem cell differentiation and promote enterocyte survival, thereby expanding differentiated epithelial cells. RNA-seq showed strong upregulation of multiple machineries involved in nutrient uptake. Indeed, multiple lipids and vitamins are altered in the serum^19,46^ and the milk and, importantly, IgA in the milk of *Rank*^*Δvil*^ dams; IgA secreted in the milk has a key role in protecting suckling infants from microbial infections^47^. Our data show that villous expansion is triggered by female pregnancy hormones and the RANK–RANKL axis based on experiments with organoids and germ-free mice. Alterations in the microbiome, M cells and/or altered IgA levels might also contribute to the observed effects in pregnancy and lactation, and thereby affect the nutritional state of mothers and offspring, which remains an open question. Unfortunately, we did not test direct nutrient absorption in pregnancy/lactation owing to ethical and technical constraints, and this will be important to address in future experiments. It is important to note that milk is not a simple product of intestinal nutrient absorption and that there are differences between rodents and humans in nutrient absorption during pregnancy and lactation, for example, for calcium absorption important for bone health^48,49^. As observed in malnutrition and famines such as the Dutch famine^42^, proper nutrition of mothers during pregnancy and lactation affects the long-term metabolism of the babies^50^. Our data now show that RANK–RANKL-dependent intestinal epithelial expansion in the mothers is important not only for offspring growth but also for their metabolic health in adult life.

The elongation and volume expansion of the villi result in the formation of more sheet-like geometries, where, similar to non-pregnant mice, the flat villi surfaces are positioned to face the flow of the intestinal content. Thus, it appears that villi expansion not only enhances the epithelial surface in the key intestinal region involved in nutrient uptake, but this morphological alignment presumably further facilitates nutritional absorption through biomechanical slowing of food movement. Our findings on RANK–RANKL-driven expansion of the intestinal epithelium through reduced cell death and stem cell maintenance identify a pathway for maternal adaptation to increased nutrient demand during pregnancy and lactation, with vital relevance to evolution and human health.

## Methods

### Mice

*Rank* conditional mice (*Rank*^*flox*^) were generated in our laboratory and have been previously described^11^. The following additional mouse strains were used: *Rankl* conditional mice (*Rankl*^*flox*^)^51^, *Traf6* conditional mice (*Traf6*^*flox*^)^31^, constitutively active *RANK* mutant over-expressing mice (*caRANK*^*LSL*^*)*^29^, *Rnf43* conditional mice (*Rnf43*^*flox*^) and *Znrf3* conditional mice (*Znrf3*^*flox*^)^28^. *Vil*^*cre*^ mice^33^. *Apc*^*min/+*^ mice^32^, *Twist2*^*cre*^ mice^52^, *Cd4*^*c*^^*re*^ mice^40^, *Rorgt*^*cre*^ mice^53^, tdTomato reporter mice^54^ and *Lgr5-eGFP-IRES-creERT2* mice^55^ were purchased from the Jackson laboratories. All mouse lines were maintained on a C57BL/6J genetic background and housed under specific pathogen-free conditions. Mouse cages were individually ventilated and subjected to ambient temperature of 22 ± 1 °C under a 14 h–10 h light–dark cycle. Mouse genotypes were assessed by PCR. For all experiments, only littermate and sex-matched mice were used, unless otherwise specified. Control littermates of *caRANK*^*vil-Tg*^ mice were defined as *Vil*^*cre*^ mice, heterozygous *caRANK*^*LSL*^ mice or wild-type mice (negative for *Vil*^*cre*^ and negative for *caRANK*^*LSL*^).

We did not observe any apparent differences among control littermates with different genotypes in any experiments. To exclude the potential effects of the *Rank* deletion in the intestine in timed pregnancy/lactation studies, *Rank*^*WT*^ and *Rank*^*Δvil*^ female littermates were crossed to wild-type syngeneic C57BL/6J male breeders, resulting in RANK-sufficient fetuses with a comparable genetic background. For the offspring analysis, we used the *Rank*^*WT*^ and *Rank*^*Δvil*^ female littermates who delivered more than five mice to avoid the effects of different offspring numbers. All mice were bred, maintained, examined and euthanized in accordance with institutional animal care guidelines and ethical animal license protocols approved by the legal authorities. All experimental animal projects performed at Institute of Molecular Biotechnology of the Austrian Academy of Sciences (IMBA), Vienna BioCenter were approved by the Federal Ministry of Education, Science and Research. Animal experiments using Germ-free mice at the University of British Columbia were approved by the University of British Columbia Animal Care Committee. Animal experiments using germ-free mice at Kiel University were approved prior to the study by the committee for animal welfare of the state of Schleswig-Holstein (V242-7224.121-33). Timed matings were performed in germ-free and specific-pathogen-free mice to achieve syngenic (both parents C57BL/6) and semiallogenic breedings (male BALB/C, female C57BL/6).

### Mouse intestinal organoids

Mouse intestinal organoids were established as described previously^21^. In brief, freshly isolated intestinal crypts were mixed with 10 μl Matrigel (Corning, 356231) and placed on a warmed 24-well plate dish to let them polymerize. The crypts were then cultured with a ROCK inhibitor (Y-27632; Sigma-Aldrich, Y0503, 10 μM) and ENR (EGF/NOGGIN/R-spondin) medium composed of advanced Dulbecco’s modified Eagle’s medium/F12 (DMEM) supplemented with penicillin–streptomycin, 10 mM HEPES, GlutaMAX, N2 (Life Technologies), B27 (Life Technologies) and 1 mM *N*-acetylcysteine (Sigma-Aldrich), 50 ng ml^−1^ mouse recombinant epidermal growth factor (EGF; Peprotech), R-spondin1 (conditioned medium from 293T-HA-RspoI-Fc cells, 10% final volume), and 100 ng ml^−1^ NOGGIN (Peprotech). Passage was performed weekly at a 1:6 split ratio. To explore ERK/MAPK-dependent phenotypes, we cultured organoids using a low concentration of EGF (50 ng ml^−1^ to 50 pg ml^−1^) in ENR medium (E^low^NR medium).

To assess organoid survival (Fig. 1f), 25 irradiated organoids were dissociated into single cells by TrypLE (Thermo Fisher Scientific) and DNase I (Worthington Biochemical) treatments for 5 min at 37 °C and subsequent vigorous pipetting through a p200 pipette. The dissociated cells were mixed with 15 μl Matrigel and seeded into each well of a 48-well plate. The organoids were then further cultured in WENR medium. WENR medium was composed of WNT3A (conditioned medium (CM) from WNT3A L-cells, 50% final volume), 10 μM Y-27632 (ROCK inhibitor, StemCell Technologies) and 10 μM nicotinamide (Sigma-Aldrich), on the basic ENR medium. Bright-field images of organoids were taken using a Carl Zeiss Axiovert.A1 microscope. For the measurement of organoid size, organoid areas in horizontal cross sections were measured using Fiji software (ImageJ, v.2.3.0).

### Human intestinal organoids

#### Patient recruitment and sample collection

Intestinal biopsy specimens were collected from the duodenum of children undergoing diagnostic endoscopy. This study was conducted with informed patient and/or caretaker consent as appropriate, and with full ethical approval by East of England - Cambridge South Research Ethics Committee (REC-12/EE/0482).

#### Human organoid cultures

Human intestinal organoids were generated from mucosal biopsy specimens by isolating intestinal crypts and culturing those in Matrigel (Corning) using medium described previously^56^. The medium was replaced every 48–72 h. Once organoids were established, they were further cultured in an expansion medium composed of advanced DMEM/F12 supplemented with penicillin–streptomycin, 10 mM HEPES, GlutaMAX, N2 (Life Technologies), B27 (Life Technologies), 1 mM *N*-acetylcysteine (Sigma-Aldrich), R-spondin1 (conditioned medium from 293T-HA-RspoI-Fc cells, 10% final volume), 100 ng ml^−1^ NOGGIN (Peprotech), 10 nM human gastrin I (Sigma-Aldrich), 500 nM A83-01 (Tocris), WNT3A (conditioned medium from WNT-producing L-cell line, 50% final volume), 50 ng ml^−1^ mouse recombinant epidermal growth factor (EGF; Peprotech), 100 ng ml^−1^ human insulin-like growth factor-1 (IGF-1; BioLegend), and 50 ng ml^−1^ human recombinant fibroblast growth factor-basic (FGF-2; Peprotech)^43^. To test the role of RANKL under suboptimal growth conditions, we used a growth-factor-reduced condition lacking EGF, IGF-1 and FGF-2 from the expansion medium. To assess organoid survival, organoids were irradiated and subsequently dissociated into single cells using TrypLE (Thermo Fisher Scientific) and DNase I (Worthington Biochemical) treatments for 5 min at 37 °C and subsequent vigorous pipetting using a p200 pipette. The dissociated cells were mixed with 15 μl Matrigel (Corning, 356231) and seeded into a 48-well plate. The organoids were then further cultured in expansion medium supplemented with the ROCK inhibitor Y-27632 (10 μM, StemCell Technologies). Human *BMPR1A* mutant organoids have been described previously^44^.

### *Apc*^*min/+*^ tumoroids

Small intestinal adenomas were collected from *Apc*^*min/+*^ heterozygous mice. Tissues were incubated with Gentle Cell Dissociation Reagent (StemCell Technologies) for 15 min at room temperature and vortexed vigorously to remove non-transformed crypts surrounding the tumour. The remaining tissue was minced into 2–5 mm fragments, and further digested in TrypLE (Thermo Fisher Scientific) and DNase I (Worthington Biochemical) for 10 min at 37 °C. The supernatant was collected and centrifuged at 300*g* for 5 min, suspended in Matrigel and seeded into a 24-well plate. The seeded cells were cultured with advanced DMEM/F12 supplemented with penicillin–streptomycin, 10 mM HEPES, GlutaMAX, N2 (Life Technologies), B27 (Life Technologies) and 50 ng ml^−1^ mouse recombinant epidermal growth factor (EGF; Peprotech). The medium was replaced every 2 days. Tumoroids were passaged every 5 days.

### Ex vivo maintenance of mesenchymal cell of the lamina propria

The protocol was adapted from a previous study^57^. In brief, half of the upper small intestinal tissue from nulliparous female mice was washed with cold PBS, Peyer’s patches were removed manually, and then the remaining specimens were incubated in 10 ml of gentle dissociation solution (HBSS with 10 mM EDTA and 1 mM DTT (Sigma-Aldrich)) on ice for 20 min. The tissues were shaken vigorously, and the supernatant was discarded. The remaining tissue fragments were cut into 2–5 mm fragments and seeded in DMEM/F12 supplemented with 10% fetal bovine serum (FBS) and 50 ng ml^−1^ mouse recombinant epidermal growth factor (EGF; Peprotech). Once mesenchymal cells started outgrowth from the tissue fragment, attached cells were dissociated with trypsin, seeded in six-well dishes and subsequently grown to expand mesenchymal cells. Then, 3 days before stimulation with recombinant mouse prolactin (rmProlactin) (Peprotech), the culture medium was changed to DMEM with 10% charcoal-stripped FBS and 50 ng ml^−1^ of EGF.

### MTT assay

Organoid growth was assessed using the 3-(4,5-dimethylthiazol-2-yl)-2,5-diphenyltetrazolium (MTT) assay. Organoids were incubated with MTT (0.5 mg ml^−1^, final concentration; Sigma-Aldrich) for 4 h at 37 °C and the cells containing formazan were subsequently solubilized with 10% SDS in 0.01 M HCl. The absorbance of the formazan product was measured at 550 nm using BioTek Synergy 2. Absorbance at 720 nm was subtracted from sample values measured at 550 nm. Furthermore, the absorbance values of wells containing Matrigel and medium, but not organoids, were subtracted as background controls.

### Flow cytometry

#### Intestinal organoids

For flow cytometry, organoids were dissociated with TrypLE (Thermo Fisher Scientific) and DNase I (Worthington Biochemical) for 5 min at 37 °C and subsequent vigorous pipetting. The cell suspension was washed with DMEM/F12 medium containing 10% FBS. Dead cells were fluorescently labelled using a fixable viability dye (eBioscience, 1:1,200). Antibody labelling of cells was performed in FACS staining buffer (PBS supplemented with 2% FCS and 2 mM EDTA) on ice for 30 min after blocking Fc receptors. Fc receptors were blocked with anti-CD16/32 antibodies (BD Pharmingen, 1:100). The following antibody was used: anti-CD44 (IM7, eBioscience, 1:200). *Lgr5*-eGFP^+^CD44^+^ cells were assessed on an LSRII cytometer (BD Biosciences) using FACSDiva (BD Biosciences). The data were analysed using the FlowJo software (Treestar).

#### Mouse lamina propria cells

A total of 20 cm of upper small intestinal tissue was washed with cold PBS, Peyer’s patches were removed manually and the remaining specimens were then incubated in 10 ml of gentle dissociation solution (HBSS with 10 mM EDTA and 1 mM DTT (Sigma-Aldrich)) on ice for 20 min. The tissues were shaken vigorously, and the supernatant was discarded. The remaining tissue fragments were washed with 10 ml of HBSS buffer, cut into 2–5 mm fragments and further digested in dissociation solution (advanced DMEM with 0.15 mg ml^−1^ of collagenase P (Roche), 0.8 mg ml^−1^ of dispase (Gibco) and 400 IU ml^−1^ of DNase I (Worthington)) using the GentleMACS dissociator (Miltenyi) at 37 °C for 1 h. The cell suspension was filtered through a 100 μm cell strainer into a 50 ml tube, then centrifuged at 300*g* for 5 min and the supernatant was discarded. Dead cells were fluorescently labelled using a fixable viability dye (eBioscience, 1:1,200). Antibody labelling of cells was performed in DMEM supplemented with 2%) on ice for 30 min after blocking Fc receptors. Fc receptors were blocked with anti-CD16/32 antibodies (BD Pharmingen, 1:100). The following antibodies was used: anti-CD31 (MEC13.3, BioLegend, 1:300) and anti-podoplanin (8.1.1, BioLegend, 1:300). TdTomato expression in podoplanin^+^CD31^−^ mesenchymal cells was assessed on the LSRII cytometer (BD Biosciences) using FACSDiva (BD Biosciences). The data were analysed using the FlowJo software (Treestar).

### 3D-imaging and quantifications of intestinal tissue

#### Tissue preparation and imaging

Intestinal tissues were fixed in 4% paraformaldehyde at 4 °C for 20 h. The fixed samples were then incubated in 1% Triton X-100 solution at 4 °C overnight for permeabilization. The tissues were subsequently incubated in DAPI (1:500; Invitrogen, D3571), phalloidin (1:400; Invitrogen, A30107) or 1,1′-dioctadecyl-3,3,3′,3′-tetramethylindodicarbocyanine, 4-chlorobenzenesulfonate salt (DiD, 1: 500: Invitrogen, D7757) at 4 °C for 48 h, followed by three washes with fresh PBS over 30 min periods. The labelled samples were transferred into RapiClear 1.49 (Sunjin Lab) overnight. The samples were mounted in a 0.50 mm i-spacer (Sunjin Lab) for confocal imaging. Images were acquired using the Zeiss LSM 700 confocal microscope. The *z*-step size was set to 2.15 μm. Arivis Vision 4D was used for 3D Image visualization as shown in Figs. 2a,c and 3a,c and Extended Data Figs. 8j and 10d,e. Imaris was used for the image visualization shown in Supplementary Videos 1–4.

#### Measurement of volume, surface area and length of villi

For measuring the volume, surface and length of the villi from three-dimensional images, a custom ImageJ macro was created. The MorpholibJ library (v.1.4.1) (https://imagej.net/plugins/morpholibj) and ImageScience library (v.3.1.0) (https://imagescience.org/meijering/software/imagescience/) were used. In the first step, the image was downsampled, the crypt region was annotated manually on several 2D-slices, then interpolated to cover the volume in 3D. The crypts were removed to focus on the villi only. To create seed objects and separate the individual villi, a combination of binary operations and Laplacian of Gaussian filtering was used iteratively. The resulting objects were then regrown to their original size by 3D watershed and villi were analysed separately. Length measurement was performed using a distance map starting at the base of the villi, then reading out the maximum intensity. Volume and surface measurements were also performed on the segmented objects using MorpholibJ library.

### EdU incorporation assay

#### Cell cycle analysis in organoids

For cell cycle analysis, organoids were incubated with 10 μM 5-ethynyl-2′-deoxyuridine (EdU) for 1 h and subsequently dissociated with TrypLE and DNase I. After dead cells were fluorescently labelled with a viability dye (eBioscience, 1:1,200), dissociated cells were fixed, permeabilized using a fixation/permeabilization kit (eBioscience) and finally stained using the Click-iT EdU kit (Life Technologies). Cell cycle stages were analysed using flow cytometry. For whole-mount imaging of EdU labelling, organoids were incubated with 10 μM EdU for 2 h and subsequently fixed and counterstained with DAPI to visualize nuclei. Images were acquired using a Zeiss LSM 700 confocal microscope.

#### In vivo labelling of epithelial cells in the mouse intestine

EdU (Sigma-Aldrich) was administered at 100 mg per kg body weight intraperitoneally. The time of day for EdU delivery was consistent for all the animals used. Then, 24 h after administration, mouse intestinal tissues were collected and fixed in 4% paraformaldehyde at 4 °C for 20 h. Whole-mount tissues were incubated in 1% Triton X-100 solution at 4 °C overnight for permeabilization. The EdU-incorporated cells were labelled using the Click-iT Edu kit (Life Technologies). The tissues were subsequently incubated in DAPI (1:500; Invitrogen, D3571) at 4 °C for 48 h, followed by three washes with fresh PBS over 30 min periods. The labelled samples were transferred into RapiClear 1.49 (Sunjin Lab) overnight. The samples were mounted in a 0.50 mm i-spacer (Sunjin Lab) for confocal imaging. Images were acquired using the Zeiss LSM 700 confocal microscope. The *z-*step size was set to 2.15 μm. Intestinal epithelial cell migration distance was defined as the distance from the crypt base to the EdU-positive cells that had migrated the farthest and was measured using Imaris software.

### Histology and immunohistochemistry

For histological analysis, the dissected mouse intestines or human intestinal organoids were fixed in 4% paraformaldehyde overnight at 4 °C, dehydrated and embedded in paraffin. 2 μm paraffin-sections were deparaffinized by xylene substitute (Thermo Fisher Scientific, Shandon) and rehydrated. Rehydrated sections were stained with H&E for morphological assessment. For immunohistochemistry, after rehydration of the sections, epitopes were retrieved using sodium citrate pH 6 with 0.05% Tween-20 for 30 min or using the BOND Enzyme Pretreatment Kit (Leica AR9551) for 5 min. The sections were blocked for 1 h in 5% BSA (VWR Life Science) and 10% goat serum (Sigma-Aldrich, 9023) and incubated with primary antibodies against phospho-histone H3 (1:100; CellPath, PBC-ACI3130C), mouse OLFM4 (1:800; Cell Signaling Technology, 39141), human OLFM4 (1:100; Cell Signaling Technology, 14369), cleaved caspase-3 (1:100; Cell Signaling Technology, 9661) or CRE (1:100, Cell Signaling Technology, 15036), all diluted in blocking solution. For detecting M cells, sections were blocked for 1 h in 2% BSA (ready to use; VWR Life Science) and 5% rabbit serum (Sigma-Aldrich, R9133) and incubated with a primary antibody against glycoprotein 2 (1:150, MBL Life Science, D278-3). The sections were subsequently incubated with a secondary antibody (HRP-polymer rabbit, DCS (PD000POL-K)) and DAB (Abcam, ab64238). Finally, the sections were counter-stained with non-acidified haematoxylin (Thermo Fisher Scientific, 6765002). For detecting CRE, TMB substrate (SZABO SCANDIC) was used as replacement for DAB, in combination with Nuclear fast red. Slides were then scanned using the Mirax Scanner (Zeiss) and representative images were acquired using the Panoramic Viewer Software v.2.4.0 (3DHistech). The sections were examined with blinding to the genotype of the mice.

### Immunofluorescence staining of frozen intestinal sections and organoids

Intestinal tissues were isolated from mice following trans-cardiac perfusion with PBS containing heparin and snap-frozen in Optimal Cutting Temperature (OCT) compound (Sakura). 14 μm cryosections were prepared, air-dried at room temperature for 1 h and subsequently fixed in ice-cold acetone at −20 °C for 10 min. The slides were blocked in 0.3% H_2_O_2_ for 60 min, Avidin/Biotin blocking buffer (Vector Laboratories) for 15 min and 10% goat serum (Alexa Fluor Tyramide SuperBoost Kit) for 60 min at room temperature, and subsequently stained with biotinylated anti-RANK antibodies (BAF692; R&D systems; 1:50) or biotinylated anti-RANKL antibodies (13-5952-82; Invitrogen; 1:150) at 4 °C overnight. The Tyramide Signal Amplification (TSA) System (Alexa Fluor Tyramide SuperBoost Kit) was used according to the manufacturer’s protocol. For further multiplexing, additional stainings were performed after the TSA fluorescence protocol. In brief, slides were stained at 4 °C overnight with anti-PDGFRα antibodies (AF1062, R&D Systems, 1:150) in 2% BSA/PBST (0.1% Tween-20), followed by donkey anti-goat Alexa Fluor 555 (A21432, Invitrogen, 1:500) as the secondary antibody. For the detection of intestinal epithelial cells, anti-EPCAM Alexa Fluor 488 (118210, BioLegend, 1:100) was used. Phalloidin and DAPI were used for membrane staining and nuclear counterstaining, respectively. Confocal images were obtained using the Zeiss LSM 700 and Zeiss LSM 710 microscopes.

For the whole-mount staining of mouse and human organoids, organoids were fixed with 4% PFA at room temperature for 15 min, followed by incubation with blocking and permeabilization solution consisting of 0.2% Triton X-100, 0.1% Tween-20, 2% BSA and 2% normal goat serum in PBS at room temperature for 1 h. Mouse organoids were stained at 4 °C overnight with anti-mouse OLFM4 (1:400; Cell Signaling Technology, 39141) in blocking and permeabilization solution. Goat anti-rabbit Alexa Fluor 633 (1:500; Invitrogen, A21072) was used as a secondary antibody. Human organoids were stained with anti-human OLFM4 antibodies (1:100; Cell Signaling, 14369) at 4 °C overnight and the TSA Fluorescence System (Alexa Fluor Tyramide SuperBoost Kit) was used according to the manufacturer’s protocol. Phalloidin and DAPI were used for membrane staining and nuclear counterstaining, respectively. Confocal images were obtained using Zeiss LSM 700 and Zeiss.

### Whole-mount imaging of the mammary gland

Mammary glands were dissected from mice, spread on glass slides and fixed in Carnoy’s fixative (60% ethanol, 30% chloroform and 10% glacial acetic acid) overnight. The slides were washed in 70% ethanol for 15 min, 30% for 15 min, rinsed in distilled water for 5 min, stained in carmine alum stain (2.5 g alum potassium sulfate and 1.0 g carmine in 500 ml of double-distilled H_2_O) and then washed in 70% ethanol until fat was clear and glands still visible. Subsequently, the slides were dehydrated in 95% ethanol and 100% ethanol for 1 h, respectively, followed by 1 h in xylene. The dehydrated samples were mounted with EukitNeo mounting medium. Whole-mount images were obtained using Zeiss Axio Zoom.V16.

### Western blotting

Western blotting was performed using standard protocols. Total protein was extracted from isolated intestinal epithelial cells. To isolate intestinal epithelial cells, intestinal tissues were minced into around 5 mm fragments and further incubated with Gentle Cell Dissociation Reagent (StemCell Technologies) for 15 min at room temperature. The tissue fragments were vigorously resuspended and isolated intestinal epithelial cells collected by passing through a 70 µm cell strainer (SZABO SCANDIC). Isolated intestinal epithelial cells were then lysed in RIPA buffer containing a cocktail of protease and phosphatase inhibitors (Thermo Fisher Scientific, 78440). Blots were blocked for 1 h with 5% bovine serum albumin (BSA) in TBST (1× Tris-buffered saline (TBS) and 0.1% Tween-20) and then incubated overnight at 4 °C with primary antibodies, diluted in 5% BSA in TBST (1:1,000 dilution). Blots were washed three times in TBST for 15 min, then incubated with horseradish peroxidase (HRP)-conjugated secondary antibodies (1:5,000 dilution; GE Healthcare, NA9340V) for 45 min at room temperature, washed three times in TBST for 15 min and visualized using enhanced chemiluminescence (ECL Plus, Pierce, 1896327). The following primary antibodies were used: anti-β-actin (1:1,000; Sigma-Aldrich, A5316); anti-IκBα (1:1,000; Cell Signaling, 9247) and anti-phospho-IκBα (Ser32/36) (1:1,000; Cell Signaling, 9246). Secondary antibodies were anti-rabbit IgG HRP (1:5,000; GE Healthcare, NA9340V) and anti-mouse IgG HRP (1:5,000; Promega, W4021).

### qPCR

Total RNA was extracted from intestinal organoids or intestinal epithelial cells. To isolate intestinal epithelial cells, intestinal tissues were minced into around 5 mm tissue pieces and then incubated with Gentle Cell Dissociation Reagent (StemCell Technologies) for 15 min at room temperature. The tissue fragments were vigorously resuspended and the isolated intestinal epithelial cells collected by passing through a 70 µm cell strainer (SZABO SCANDIC). Total RNA isolation was performed using the RNA isolation kit (VBCF) which uses a lysis step based on guanidine thiocyanate (adapted from ref. ^58^) and magnetic beads (GE Healthcare, 65152105050450) applied on a KingFisher instrument (Thermo Fisher Scientific). After 5 min incubation at room temperature, DNA was digested with DNase I (New England BioLabs) for 15 min at room temperature, followed by a series of washing steps. RNA was eluted from the beads in 50 μl RNase-free water for 2 min at room temperature. Equivalent quantities of total RNA were reverse transcribed to synthesize cDNA using a LunaScript RT SuperMix Kit (New England BioLabs). qPCR was performed using Luna Universal qPCR master Mix (New England BioLabs). Primer sequences were as follows: *Gapdh* forward, CATCACTGCCACCCAGAAGACTG; *Gapdh* reverse, ATGCCAGTGAGCTTCCCGTTCAG; *Rank* forward, CCCAGGAGAGGCATTATGAG; *Rank* reverse, CACACACTGTCGGAGGTAGG; *Rankl* forward, GTGAAGACACACTACCTGACTCC; *Rankl* reverse, GCCACATCCAACCATGAGCCTT; *Birc2* forward, CCACTTCAGACACCCCAGGA; *Birc2* reverse, TTCCGAACTTTCTCCAGGGC; *Birc3* forward, GCGTTCAGAGCCTAGGAAGT; *Birc3* reverse, GTGAGATGACAGGGAGGGGA; *Tnfaip3* forward, AGCAAGTGCAGGAAAGCTGGCT; *Tnfaip3* reverse, GCTTTCGCAGAGGCAGTAACAG; *Bcl2* forward, CCTGTGGATGACTGAGTACCTG; *Bcl2* reverse, AGCCAGGAGAAATCAAACAGAGG; *Bcl2l1* forward, GCCACCTATCTGAATGACCACC; *Bcl2l1* reverse, AGGAACCAGCGGTTGAAGCGC; *Lgr5* forward, CGGGACCTTGAAGATTTCCT; *Lgr5* reverse, GATTCGGATCAGCCAGCTAC; *Bmp2* forward, TGCTTCTTAGACGGACTGCG; *Bmp2* reverse, TGGGGAAGCAGCAACACTAG; *Id2* forward, CCAGAGACCTGGACAGAACC; *Id2* reverse, CGACATAAGCTCAGAAGGGAAT; *Id3* forward, AGCTCACTCCGGAACTTGTG; *Id3* reverse, AGAGTCCCAGGGTCCCAAG; *GABDH* forward, AATGAAGGGGTCATTGATGG; *GABDH* reverse, AAGGTGAAGGTCGGAGTCAA; *BIRC2* forward, CAGACACATGCAGCTCGAATGAG; *BIRC2* reverse, CACCTCAAGCCACCATCACAAC; *BIRC3* forward, GCTTTTGCTGTGATGGTGGACTC; *BIRC3* reverse, CTTGACGGATGAACTCCTGTCC; *TNFAIP3* forward, CTCAACTGGTGTCGAGAAGTCC; *TNFAIP3* reverse, TTCCTTGAGCGTGCTGAACAGC; *BCL2L1* forward, GCCACTTACCTGAATGACCACC; *BCL2L1* reverse, AACCAGCGGTTGAAGCGTTCCT; *BMP2* forward, TGTATCGCAGGCACTCAGGTCA; *BMP2* reverse, CCACTCGTTTCTGGTAGTTCTTC; *ID2* forward, TTGTCAGCCTGCATCACCAGAG; *ID2* reverse, AGCCACACAGTGCTTTGCTGTC; *OLFM4* forward, GACCAAGCTGAAAGAGTGTGAGG; *OLFM4* reverse, CCTCTCCAGTTGAGCTGAACCA.

### QuantSeq 3′ mRNA-seq

#### Library preparation

The protocol for total RNA extraction was performed as described above in the ‘qPCR’ section. RNA quantification and quality control were performed using a DNF-471 Standard Sensitivity RNA Analysis kit (Agilent) with a fragment analyzer. Equivalent quantities of total RNA were used for library preparation using the Lexogen QuantSeq 3′ mRNA-Seq Library Prep Kit FWD from Illumina. The DNF-474 High Sensitivity NGS Fragment Analysis Kit (1–6,000 bp) (Agilent) was used to determine the quality of the library with a fragment analyzer. 3′ RNA-seq (QuantSeq) reads were prepared for analysis by removing adaptor contamination, poly(A) read through and low-quality tails using bbmap v.36.92. Libraries were pooled at an equimolar ratio and sequenced on an Illumina HiSeq 2500 instrument using the single-read 50-read mode.

#### Data analysis

RNA-seq reads were trimmed using BBDuk v38.06 (ref=polyA.fa.gz,truseq.fa.gz k=13 ktrim=r useshortkmers=t mink=5 qtrim=r trimq=10 minlength=20). Reads mapping to abundant sequences included in the iGenomes UCSC GRCm38 reference (mouse rDNA, mouse mitochondrial chromosome, phiX174 genome, adapter) were removed using bowtie2 v.2.3.4.1 alignment. The remaining reads were analysed using genome and gene annotation for the GRCm38/mm10 assembly obtained from *Mus musculus* Ensembl release 94. Reads were aligned to the genome using star v.2.6.0c and reads in genes were counted with featureCounts (subread v.1.6.2) using strand-specific read counting for QuantSeq experiments (-s 1). Differential gene expression analysis on raw counts was performed using DESeq2, over-representation analysis with clusterProfiler v.4.4.4 and gene set enrichment analysis with fgsea v.1.22.0. The relevant signalling processes and biological functions were evaluated using the commercial QIAGEN’s Ingenuity Pathway Analysis (IPA, Qiagen; www.qiagen.com/ingenuity) software. The *z* score was applied to predict a cellular process’ directional change, such as activating or inhibiting a cellular pathway. The Benjamini–Hochberg method was used to adjust canonical pathway *P* values.

### Single-cell sorting of mouse intestinal lamina propria cells for sequencing

The protocol was modified from a previous study^26^. In brief, 20 cm of upper small intestinal tissue was carefully washed with cold PBS, Peyer’s patches were removed manually, and then the remaining specimens were incubated in 10 ml of gentle dissociation solution (HBSS with 10 mM EDTA and 1 mM DTT (Sigma-Aldrich)) on ice for 20 min. The tissues were shaken vigorously, and the supernatant was collected in a new conical tube, washed with HBSS buffer and suspended in 10 ml of HBSS buffer, suspension ‘A’. The remaining tissue fragments were washed with 10 ml of HBSS buffer, cut into 2–5 mm fragments and were further digested in dissociation solution (advanced DMEM with 0,15 mg ml^−1^ of collagenase P (Roche), 0.8 mg ml^−1^ of dispase (Gibco), and 400 IU ml^−1^ of DNase I (Worthington)) using a GentleMACS dissociator (Miltenyi) at 37 °C for 1 h. The cell suspension was filtered through a 100 μm cell strainer into a 50 ml tube, then centrifuged at 300*g* for 5 min, and the supernatant was discarded. The cell pellets were then combined with the cell suspension ‘A’. Dead cells were fluorescently labelled using a fixable viability dye (eBioscience, 1:1,200). Antibody labelling of cells was performed in DMEM supplemented with 2%) on ice for 30 min after blocking Fc receptors. Fc receptors were blocked with anti-CD16/32 antibodies (BD Pharmingen, 1:100). The following antibodies were used: anti-CD45 (IM7, eBioscience, 1:400) and EPCAM (G8.8, BioLegend, 1:800). Using FACSAria III Cell Sorter (BD), CD45-positive and EPCAM-negative cells (Immune cells) and CD45^−^ and EPCAM^−^ cells (mesenchymal cells) were enriched by cell sorting.

### scRNA-seq

#### Library preparation from mouse intestinal organoids

Control mouse intestinal organoids and organoids cultured in the presence of recombinant mouse RANK ligand (rmRANKL, Oriental Yeast) for 12 h were dissociated with TrypLE (Thermo Fisher Scientific) and DNase I (Worthington Biochemical) for 5 min at 37 °C and subsequent vigorous pipetting through a p200 pipette. The cell suspension was washed with DMEM/F12 medium containing 10% FBS. Cell viability and efficiency of dissociation were determined using Nucleocounter NC-250 (Chemometec) before the single cells were loaded into one channel of a 10x Chromium microfluidics chip to package them into one library. scRNA-seq libraries were generated using 10x Genomics kits. The libraries were sequenced on an Illumina NovaSeq 6000.

#### Library preparation from mouse intestinal lamina propria cells

For each sample, 1 million cells were fixed for 22 h at 4 °C, quenched and stored at −80 °C according to 10x genomic Fixation of Cells & Nuclei for Chromium Fixed RNA profiling (CG000478, 10X Genomics, Pleasanton, CA) using the Chromium Next GEM Single Cell Fixed RNA Sample preparation kit (PN-1000414, 10X Genomics). In total, 250,000 cells per sample were used for probe hybridization using the Chromium Fixed RNA Kit, mouse WTA probes (PN-1000496, 10X Genomics), pooled at equal numbers and washed following the Pooled Wash Workflow following the Chromium Fixed RNA Profiling Reagent kit protocol (CG000527, 10X Genomics). GEMs were generated using Next GEM ChipQ (PN-1000422, 10X Genomics) on the Chromium X (10X Genomics) system with a target of 10,000 cells recovered and libraries prepared according to the manufacturer instructions (CG000527, 10x Genomics). Sequencing was performed using NovaSeq S4 lane PE150 (Illumina) with a target of 15,000 reads per cell. Alignment of the samples was performed using the 10x Genomics Cell Ranger 7.1.0 multi pipeline.

#### Data analysis (mouse intestinal organoids)

Reads were aligned to the reference mouse genome (mm10) downloaded from the 10x Genomics website (v.2020-A) using the Cell Ranger (v.5.0.1) count function with the default parameters. Genome annotation corresponded to Ensembl v98. The median number of unique molecular identifiers (UMIs) per cell was between 23,501 and 25,058, with a median of 3,808–4,300 genes detected per condition. The computational analysis of the 10x Genomics UMI count matrices was performed using the R package Seurat (v.4.0.5). Cells were subjected to a quality-control step, keeping those cells expressing more than 1,000 genes and with less than 20% of UMIs assigned to mitochondrial genes. Those thresholds were chosen after visual inspection of the distributions. Using this filtering, we retained between 844 and 2,299 cells, with a median of 3,877–4,512 genes per cell detected per condition. Genes expressed in less than three cells for a sample independently or in less than five cells when the samples were merged, were removed from the analyses. Each dataset was subjected separately to normalization, identification of highly variable genes and scaling using the SCTransform function. After obtaining principal components with RunPCA for each sample independently, we integrated them using reciprocal PCA (RPCA) to identify anchors with the FindIntegrationAnchors function (setting the reduction parameter to rpca), as we expect some cell type differences after rmRANKL treatment, therefore avoiding a possible overintegration.

To annotate cell populations, we performed an unsupervised clustering analysis using the Louvain algorithm with a resolution of 0.7 in a shared nearest neighbours graph constructed with the first 20 principal components, as implemented in the FindClusters and FindNeighbors Seurat functions. Nonlinear dimensional reduction for visualization was performed using the RunUMAP function with the same principal components. Cluster 6 was further subdivided in an unsupervised manner using the FindSubCluster function with a 0.6 resolution, enabling us to separate goblet and Paneth cells without splitting the rest of the clusters any further. Markers in each cluster were identified using the FindConservedMarkers and FindAllMarkers functions in the log-normalized counts by using the Wilcoxon rank-sum test. Genes with *P* value < 0.05 (adjusted by Bonferroni’s correction) and a log_2_-transformed fold change of >0.25 were retained. Clusters were annotated in accordance with those makers, as well as considering small intestinal cell-type markers from previous studies^22,26,59^. To further confirm our classifications, cell type annotations from the small intestine scRNA-seq dataset from a previous study^22^ were transferred using the TransferData function in Seurat after removing distal cells and simplifying the TA annotation in the reference. We used UCell to obtain scores for gene sets of interest in each cell. The plots were generated using the DimPlot and VlnPlot functions from Seurat as well as the ggplot2 and pheatmap R libraries.

#### Data analysis (human intestinal crypt cells)

For the computational analysis of scRNA-seq data from human intestinal crypt cells, the 10x Genomics scRNA-seq expression matrix of human intestinal crypt cells from a previous study^43^ was downloaded from the Gene Expression Omnibus (GSM3389578). Cells were already filtered in the dataset. Clustering and UMAP dimensionality reduction were performed with Seurat using similar parameters as in their study, that is, considering the first 25 principal components and a k.param of 20 for FindNeighbors and a resolution of 0.6 in FindClusters. A small cluster corresponding to non-epithelial cells was detected and removed from the analyses, redoing the downstream analyses and unsupervised clustering with a 0.8 resolution. The clusters were annotated considering markers and labels from the original paper.

#### Data analysis (mouse lamina propria cells)

Sample demultiplexing and read alignment were performed using the Cell Ranger (v.7.2.0) multi-function with the default parameters, considering the reference mouse genome (mm10) downloaded from the 10x Genomics website (v.2020-A) and the Chromium_Mouse_Transcriptome_Probe_Set_v1.0.1_mm10-2020-A.csv probe set. The median number of UMIs per cell was between 4,063 and 5,902, with a median of 2,050–2,589 genes detected per condition. The computational analysis of the 10x Genomics UMI count matrices was performed using Seurat (v.4.2.0). Cells were subjected to a quality control step, keeping those cells expressing more than 500 genes, 1,000 UMIs and with less than 5% of UMIs assigned to mitochondrial genes and cells considered singlets by scDblFinder (v.1.12.0) with the default parameters. Using this filtering, we retained between 6,822 and 9,396 cells, with a median of 2,008–2,451 genes per cell detected per condition. Genes expressed in less than three cells for a sample independently were removed from the analyses. Each dataset was subjected separately to normalization, identification of highly variable genes and scaling using the SCTransform function with vst.flavor v.2. We integrated the data using canonical correlation analysis.

To annotate cell populations, we performed an unsupervised clustering analysis using the Louvain algorithm with a resolution of 0.5 in a shared nearest-neighbours graph constructed with the first 17 principal components. Cluster 5 and 12 were subset, reintegrated with canonical correlation analysis after normalization with SCTransform and reclustered in an unsupervised manner with a 0.8 resolution and 15 principal components, allowing to further separate CD4 T cells and ILCs. Markers in each cluster were identified using the FindConservedMarkers and FindAllMarkers functions in the log-normalized counts by using the Wilcoxon rank-sum test. Genes with *P* value < 0.05 (adjusted by Bonferroni’s correction) and a log_2_-transformed fold change of >0.25 were retained. Clusters were annotated in accordance with those markers, as well as considering small intestinal cell type markers as follows; naive CD4 T cells: *Ccr7*, *Klf2*, *Sell*. Activated T cells: *Cd40Ig*, *Cd4*. Th1 cells: *Il12rb2*, *Ccr5*. T helper 17 cells: *Il17a*, *Il17f*, *Rora*. Regulatory T cells: *Foxp3*, *Ctla4*, *Il10*, *Tnfrsf4*. Memory T cells: *Zbtb16*, *Zfp683*. CD8 T cells: *Cd8a*, *Itgae*, *Gzma*. CD4^−^CD8^−^ T cells: *Trdc*, *Cd163l1*, *Ly6g5b*, *Cd3e*. ILC1: *Tbx21*, *Tyrobp*, *Ccl3*, *Xcl1*, *Il13*. ILC2: *Gata3*, *IL17rb*, *Hs3st1*. ILC3: *Rorc*, *Il22*, *Slc6a20a*. B cells: *Cd79a*, *Cd19*, *Pax5*. Plasma cells: *Igha*, *Igkc*, *Jchain*, *Xbp1*, *Mzb1*. Macrophage: *Cd14*, *Unc93b1*, *Lyz2*, *Il1b*. PDGFRA^low^CD81^+^ trophocytes: *Cd81*, *Ackr4*, *Cd34*, *Grem1*, *Col14a1*, *Dcn*. PDFGRA^low^GREM1^med^ stromal cells: *Dkk2*, *Wnt2b*. PDGFRA^low^GREM1^−^ stromal cells*: Sfrp1*, *Frzb*, *Fgfr2*. PDGFRA^high^ telocytes: *Pdgfra*, *Bmp7*, *Bmp5*, *Wif1*, *Chrd*, *Dkk3*. Myofibroblast: *Myh11*, *Hhip*, *Npnt*. Smooth muscle cells: *Atp1b2*, *Des*, *Fhl5*, *Rgs4*. Vascular endothelial cells: *Pecam1*, *Plvap*, *Flt1*. Lymphatic endothelial cells: *Lyve1*, *Mmrn1*, *Rspo3*. Glia cells: *Gpr37l1*, *Sox10*, *Kcna1*.

### Collection of milk and serum

Lactating female mice were separated from their offspring at lactation day 8 and fasted for 5 h from 10:00 to 15:00. Subsequently, they were anaesthetized with isoflurane (2% induction and 1% maintenance) and injected with 2 IU of oxytocin (Sigma-Aldrich, O3251) intraperitoneally. Expressed milk was collected with a P20 pipette. Serum was collected from inferior vena cava from mice anaesthetized with ketamine–xylazine and pooled into Micro sample tube Lithium heparin (Sarstedt). Serum samples were centrifuged at 2,000*g* for 10 min at room temperature twice to separate from cells. All samples were then stored at −80 °C for further analysis. The concentrations of milk IgA and IgG were measured with an ELISA kit (Bethyl Laboratories).

### MS analysis

Samples were prepared by adding 100 μl of a methanol/ethanol mixture (4:1, v/v) to 25 μl of the respective serum or milk samples in a 1.5 ml tube, followed by vortexing, incubation and centrifugation. The supernatants were transferred to HPLC vials and measured consecutively with reversed-phase (RP) and hydrophilic interaction chromatography (HILIC) on-line coupled to liquid chromatography–tandem mass spectrometry (LC–MS/MS). Then, 2.5 μl of each sample was pooled for quality control. Metabolite extracts were separated (HILIC) on a SeQuant ZIC-pHILIC HPLC column (Merck, 100 × 2.1 mm; 5 µm) or a RP-column (Waters, ACQUITY UPLC HSS T3 150 × 2.1; 1.8 μm) with a flow rate of 100 µl min^−1^, using the Ultimate 3000 HPLC system coupled to a Q-Exactive Focus (both Thermo Fisher Scientific). In HILIC, the gradient was ramped up in 21 min from 90% A (100% acetonitrile) to 60% B (25% ammonium bicarbonate in water). In RP, the 20 min gradient started with 99% A (0.1% formic acid in water) and ramped up to 60% B (0.1% formic acid in acetonitrile). Eluting compounds were directly ionized by electrospray ionization in polarity switching mode. Spectra were acquired in data-dependent acquisition mode using high-resolution tandem mass spectrometry. The ionization potential was set to +3.5/−3.0 kV, the sheath gas flow was set to 20, and an auxiliary gas flow of 5 was used. Obtained datasets were processed by Compound Discoverer 3.0 (Thermo Fisher Scientific). Annotation was conducted by searching the metabolite databases (mzCloud, our in-house database, ChemSpider, BioCyc, Human Metabolome Database, KEGG, MassBank and MetaboLights) with a mass accuracy of 3 ppm for precursor masses and, if applicable, 10 ppm for fragment ion masses.

For measurement of triglycerides with LC–MS, lipids were extracted using chloroform–methanol extraction from each sample. The chloroform phase was removed and diluted 1:1 with methanol and 1 μl of each sample was directly injected on a Kinetex C8 column (100 Å, 150 × 2.1 mm) using a 20 min gradient of 80% A (60% acetonitrile, 10 mM ammonium acetate, 0.1% formic acid, 40% water) to 95% B (90% isopropanol, 10 mM ammonium acetate, 0.1% formic acid and 5% water) using a flow rate of 100 µl min^−1^ and a 60 °C column temperature. Triglycerides were detected and quantified in the positive-ion mode as their ammonium adducts.

### Metabolic studies

For analysis of offspring delivered from *Rank*^*WT*^ or *Rank*^*Δvil*^ female mice, the mice were fed normal chow from weaning age until 4 weeks of age, after which they were fed normal chow or HFD (60% kcal% fat, Research Diets, D12492i) for up to 25 weeks. The pups were weekly weighed starting from postnatal day 7 until 25 weeks. For oral glucose-tolerance tests, mice (aged 25 weeks) were fasted overnight and were then administrated an oral glucose bolus by gavage (2 g per kg for normal chow-fed mice and 1 g per kg for HFD-fed mice). Glucose concentrations were measured using glucometers from blood taken by tail nick at 0, 15, 30, 45, 60 and 120 min after glucose ingestion, using a handheld blood glucose meter (One Touch UltraEasy; Lifescan). The area under the glucose-tolerance test curve was calculated for each mouse using GraphPad Prism v.9.3.1c (GraphPad Software). For analysis of insulin levels, tail-vein blood samples were added to Micro sample tube Lithium heparin (Sarstedt) to avoid blood clotting. Plasma insulin levels were measured using the Alpco Mouse Ultrasensitive Insulin ELISA (80-INSMSU-E10). For each litter of offspring, blood samples were taken the same time of the day.

### Statistics and reproducibility

All values are expressed as means ± s.e.m. GraphPad Prism 8 software was used to perform statistical analyses. All details of the statistical tests used are stated in the figure legends. Two-tailed Student’s *t*-tests, two-tailed Mann–Whitney *U*-tests and one-way ANOVA with two-tailed Tukey’s test were used as described in the figure legends. Two-way ANOVA was used to compare two groups over time. Survival curves were compared using the log-rank (Mantel–Cox) test. Unless otherwise specified in the main text or figure legends, all experiments reported in this study were repeated at least two independent times.

### Reporting summary

Further information on research design is available in the Nature Portfolio Reporting Summary linked to this article.

## Online content

Any methods, additional references, Nature Portfolio reporting summaries, source data, extended data, supplementary information, acknowledgements, peer review information; details of author contributions and competing interests; and statements of data and code availability are available at 10.1038/s41586-024-08284-1.

## Supplementary information

Supplementary Fig. 1 Uncropped images of western blots shown in this study.

Reporting Summary

Supplementary Video 1 A representative video showing 3D reconstructions of intestinal tissue from nulliparous *Rank*^*WT*^ female determined using confocal microscopy. Phalloidin indicates actin filaments and DAPI shows nuclei. Grid spacing is 200  μm.

Supplementary Video 2 A representative video showing 3D reconstructions of intestinal tissue from lactating *Rank*^*WT*^ (5 days after delivery, L5) female determined using confocal microscopy. Phalloidin indicates actin filaments and DAPI shows nuclei. Grid spacing is 200 μm.

Supplementary Video 3 A representative video showing 3D reconstructions of intestinal tissue from nulliparous *Rank*^*ΔVil*^ female determined using confocal microscopy. Phalloidin indicates actin filaments and DAPI shows nuclei. Grid spacing is 200 μm.

Supplementary Video 4 A representative video showing 3D reconstructions of intestinal tissue from lactating L5 *Rank*^*ΔVil*^ female determined using confocal microscopy. Phalloidin indicates actin filaments and DAPI shows nuclei. Grid spacing is 200 μm.

## Source data

Source Data Fig. 1

Source Data Fig. 2

Source Data Fig. 3

Source Data Fig. 4

Source Data Fig. 5

Source Data Extended Data Fig. 1

Source Data Extended Data Fig. 2

Source Data Extended Data Fig. 4

Source Data Extended Data Fig. 5

Source Data Extended Data Fig. 6

Source Data Extended Data Fig. 7

Source Data Extended Data Fig. 8

Source Data Extended Data Fig. 9

Source Data Extended Data Fig. 10

Source Data Extended Data Fig. 11

Source Data Extended Data Fig. 12

Source Data Extended Data Fig. 13

Source Data Extended Data Fig. 14

Source Data Extended Data Fig. 15

## Data Availability

Datasets generated in this study are available at the Gene Expression Omnibus (GSE225514). The 10x Genomics scRNA-seq expression matrix of human intestinal crypt cells was downloaded from the NCBI (https://www.ncbi.nlm.nih.gov/geo/query/acc.cgi?acc=GSM3389578). Source data are provided with this paper.

## Code availability

A GitHub repository (https://github.com/AsierUllate/Intestinal_Organoids) is available for this study.
