# Supplementary material for: RANK drives structured intestinal epithelial expansion during pregnancy
Source: Nature. 2024 Dec 4;637(8044):156–66. doi: 10.1038/s41586-024-08284-1 (PMC11666467; doi:10.1038/s41586-024-08284-1)
Supplement: Supplementary file 1 — Uncropped images of western blots shown in this study. [file 41586_2024_8284_MOESM1_ESM.pdf]

nature portfolio

https://doi.org/10.1038/s41586-024-08284-1

Supplementary information

RANK drives structured intestinal epithelial expansion during pregnancy

In the format provided by the authors and unedited

Gel #2

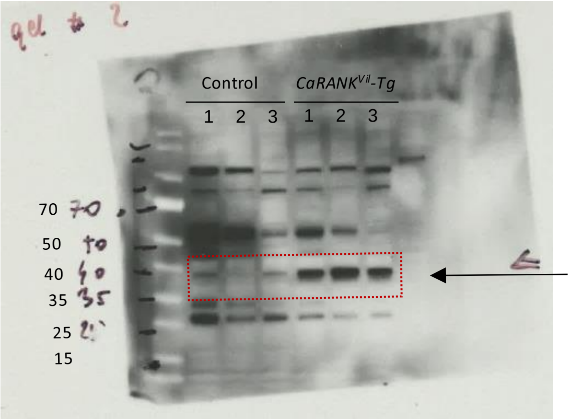

Phospho-IκBα (39kDa)

Gel #1

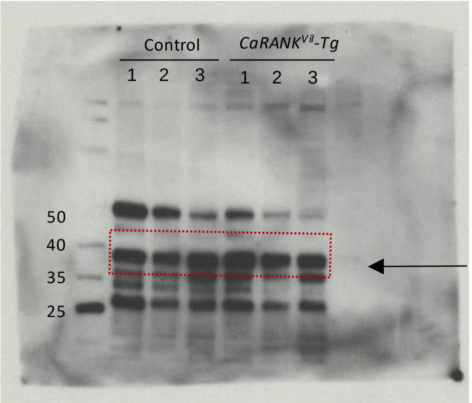

IκBα (39kDa)

Gel #2

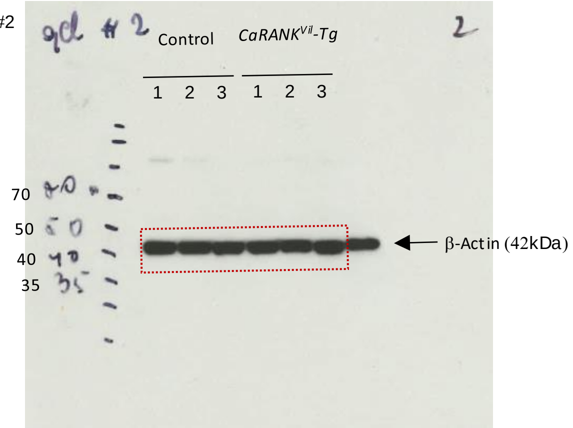

β-Actin (42kDa)

Gel #1

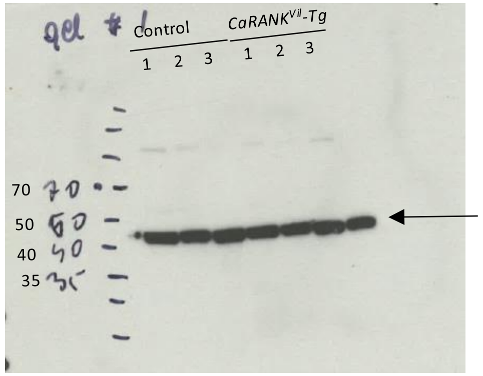

β-Actin (42kDa)

Used in Extended Data Figure 6E
