# Supplementary material for: RANK drives structured intestinal epithelial expansion during pregnancy
Source: Nature. 2024 Dec 4;637(8044):156–66. doi: 10.1038/s41586-024-08284-1 (PMC11666467; doi:10.1038/s41586-024-08284-1)
Supplement: Supplementary file 2 — Reporting Summary [file 41586_2024_8284_MOESM2_ESM.pdf]

nature portfolio

Corresponding author(s): Josef Penninger
Masahiro Onji

Last updated by author(s): Sep 24, 2024

Reporting Summary

Nature Portfolio wishes to improve the reproducibility of the work that we publish. This form provides structure for consistency and transparency in reporting. For further information on Nature Portfolio policies, see our Editorial Policies and the Editorial Policy Checklist.

Statistics

For all statistical analyses, confirm that the following items are present in the figure legend, table legend, main text, or Methods section.

n/a	Confirmed	
☐	☒	The exact sample size (*n*) for each experimental group/condition, given as a discrete number and unit of measurement
☐	☒	A statement on whether measurements were taken from distinct samples or whether the same sample was measured repeatedly
☐	☒	The statistical test(s) used AND whether they are one- or two-sided<br>*Only common tests should be described solely by name; describe more complex techniques in the Methods section.*
☐	☒	A description of all covariates tested
☐	☒	A description of any assumptions or corrections, such as tests of normality and adjustment for multiple comparisons
☐	☒	A full description of the statistical parameters including central tendency (e.g. means) or other basic estimates (e.g. regression coefficient) AND variation (e.g. standard deviation) or associated estimates of uncertainty (e.g. confidence intervals)
☐	☒	For null hypothesis testing, the test statistic (e.g. *F*, *t*, *r*) with confidence intervals, effect sizes, degrees of freedom and *P* value noted<br>*Give P values as exact values whenever suitable.*
☒	☐	For Bayesian analysis, information on the choice of priors and Markov chain Monte Carlo settings
☒	☐	For hierarchical and complex designs, identification of the appropriate level for tests and full reporting of outcomes
☒	☐	Estimates of effect sizes (e.g. Cohen's *d*, Pearson's *r*), indicating how they were calculated

Our web collection on statistics for biologists contains articles on many of the points above.

Software and code

Policy information about availability of computer code

Data collection	Flow cytometry data was acquired using multi-colour flow cytometer (LSR FortessaTM from BD Bioscience) equipped with the FACSDivaTM software (BD Bioscience). Bright-field images of organoids were taken using a Carl Zeiss Axiovert.A1 microscope. Whole-mount images of mammary glands were obtained using Zeiss Axio Zoom.V16. Confocal images were obtained using a Zeiss LSM 700/LSM 710 microscopes equipped with Zeiss LSM Software (Zeiss). For QuantSeq data 3' RNA-Seq (Quantseq), reads were prepared for analysis by removing adapter contamination, polyA read through, and low quality tails using bbmap v36.92. Libraries were pooled at an equimolar ratio and sequenced on an Illumina HiSeq 2500 instrument (Illumina, San Diego, CA, USA) using the single-read 50-read mode. scRNA-seq libraries were generated using 10x Genomics kits. The libraries were sequenced on an Illumina NovaSeq 6000 (Illumina, San Diego, CA, USA). Histological slides were scanned on a Mirax Scanner (Zeiss) and representative images were acquired using the Panoramic Viewer Software v.2.4.0 (3DHistech Ltd).
Data analysis	Flow cytometry data were analyzed using a FACSAria sorter using FlowJo v10.8.1 (Tree Star). Confocal images were processed and analyzed using Fiji software (ImageJ v.2.3.0) or Imaris software (10.0.0). Statistical analyses were performed using GraphPad Prism v.8, v.9.3.1c, and v10.0.3 (GraphPad Software Inc.). For measuring the volume, surface and length of the villi from 3-dimensional images, a custom ImageJ macro was created. The MorpholibJ library (v.1.4.1) (https://imagej.net/plugins/morpholibj) and ImageScience library 3.1.0 (https://imagescience.org/meijering/software/featurej/) were used. To create seed objects and separate the individual villi, a combination of binary operations and Laplacian of Gaussian (LoG) filtering was used iteratively. Volume and surface measurements were also performed on the segmented objects using MorpholibJ library (v.1.4.1). For the measurement of organoid size from bright-field images, organoid areas in horizontal cross sections were measured using Fiji software (ImageJ v.2.3.0).<br>For Quantseq RNAseq, RNA-seq reads were trimmed using BBDuk v38.06 (ref=polyA.fa.gz,truseq.fa.gz k=13 ktrim=r useshortkmers=t mink=5 qtrim=r trimq=10 minlength=20). Reads mapping to abundant sequences included in the iGenomes UCSC GRCm38 reference (mouse rDNA, mouse mitochondrial chromosome, phiX174 genome, adapter) were removed using bowtie2 v2.3.4.1 alignment. The remaining reads were analyzed using genome and gene annotation for the GRCm38/mm10 assembly obtained from Mus musculus Ensembl release 94. Reads were

aligned to the genome using star v2.6.0c and reads in genes were counted with featureCounts (subread v1.6.2) using strand-specific read counting for QuantSeq experiments (-s 1). Differential gene expression analysis on raw counts was performed using DESeq2 (v1.18.1), overrepresentation analysis with clusterProfiler v4.4.4 and gene set enrichment analysis with fgsea v1.22.0. The relevant signaling processes and biological functions were evaluated using the commercial QIAGEN's Ingenuity® Pathway Analysis (IPA®, QIAGEN Redwood City, www.qiagen.com/ingenuity) software. The z-score was applied to predict a cellular process' directional change, such as activating or inhibiting a cellular pathway. The Benjamini-Hochberg method was used to adjust canonical pathway p-values.
The analysis of single-cell RNA-seq data was performed using publicly available softwares and R packages as follows. Reads were aligned to the reference mouse genome (mm10) downloaded from the 10x Genomics website (version 2020-A) using the Cell Ranger (v5.0.1, https://support.10xgenomics.com/single-cell-gene-expression/software/downloads/latest) count function with default parameters. Genome annotation corresponded to Ensembl v98. The computational analysis of the 10x Genomics UMI count matrices was performed in R v4.0.5 using several functions of the R package Seurat (v4.0.5, https://github.com/satijalab/seurat/). Normalization, identification of highly variable genes and scaling was done with the SCTransform function. principal components were obtained with the RunPCA function for each sample independently, which were integrated utilizing reciprocal PCA (RPCA) to identify anchors with the FindIntegrationAnchors function (setting the reduction parameter to "rpca"). Cells were clustered in an unsupervised manner using the FindNeighbors (with 20 PCs) and the FindClusters functions (with a resolution parameter of 0.7). The data were projected using Uniform Manifold Approximation and Projection (UMAP) considering the first 20 PCs. To further subdivide Cluster 6 we used the FindSubCluster function (0.6 resolution). Markers genes were identified using the FindConservedMarkers and FindAllMarkers functions considering the Wilcoxon Rank Sum test. To help our classifications, we annotated the cells against a reference with the TransferData function. Cell scores for gene sets were calculated with the AddModuleScore_UCell function from the UCell R package (v1.1.1, https://github.com/carmonalab/UCell). To compare the scores for each celltype between conditions we used the rstatix R package (v0.7.0, https://github.com/kassambara/rstatix) to perform a Wilcoxon test with the wilcox_test function and adjusted the p-value by the Benjamini-Hochberg correction with the adjust_pvalue function. The significance was plotted in the violin plots using the add_pvalue function from the ggprism R package (v1.0.3, https://csdaw.github.io/ggprism/).
For the scRNA-seq analysis for mouse lamina propria, reads were demultiplexed and aligned to the same mouse reference genome and the Chromium_Mouse_Transcriptome_Probe_Set_v1.0.1_mm10-2020-A.csv probe set using the Cell Ranger (v7.2.0) multi function with default parameters. Doublets were retrieved with scDblFinder (v1.12.0) with default parameters. The computational analysis of the 10x Genomics UMI count matrices was performed using several functions of the R package Seurat (v4.2.0) in a similar way to the other scRNA-seq experiment. As a difference, samples were integrated with the IntegrateData function using Canonical Correlation analysis and cells were clustered using the FindNeighbors (with 17 PCs) and the FindClusters (with a resolution of 0.5) functions. Clusters 5 and 12 were further subclustered independently using a similar procedure as for the full dataset.

For manuscripts utilizing custom algorithms or software that are central to the research but not yet described in published literature, software must be made available to editors and reviewers. We strongly encourage code deposition in a community repository (e.g. GitHub). See the Nature Portfolio guidelines for submitting code & software for further information.

Data

Policy information about availability of data

All manuscripts must include a data availability statement. This statement should provide the following information, where applicable:

- Accession codes, unique identifiers, or web links for publicly available datasets
- A description of any restrictions on data availability
- For clinical datasets or third party data, please ensure that the statement adheres to our policy

RNAseq data is accessible through GSE225514.
Code and data to reproduce the scRNAseq analysis in this study is shared publicly on GitHub: https://github.com/AsierUllate/Intestinal_Organoids

Research involving human participants, their data, or biological material

Policy information about studies with human participants or human data. See also policy information about sex, gender (identity/presentation), and sexual orientation and race, ethnicity and racism.

Reporting on sex and gender	The participant was recruited randomly without any gender bias. The participant used in this article is a 14 year old healthy female.
Reporting on race, ethnicity, or other socially relevant groupings	Race, ethnicity, and other socially relevant groupings were not considered and are not reported on in this study design.
Population characteristics	No data on population characteristics was collected/used.
Recruitment	Intestinal biopsy specimens were collected from the duodenum of adolescents undergoing routine diagnostic endoscopy. This study was conducted with informed patient and/or caretaker consent as appropriate, and with full ethical approval (REC-12/EE/0482).
Ethics oversight	The study has been ethically approved by East of England - Cambridge South Research Ethics Committee

Note that full information on the approval of the study protocol must also be provided in the manuscript.

Field-specific reporting

Please select the one below that is the best fit for your research. If you are not sure, read the appropriate sections before making your selection.

☒ Life sciences ☐ Behavioural & social sciences ☐ Ecological, evolutionary & environmental sciences

For a reference copy of the document with all sections, see nature.com/documents/nr-reporting-summary-flat.pdf

Life sciences study design

All studies must disclose on these points even when the disclosure is negative.

Sample size	No statistical methods were used to predetermine the sample size for experiments. All sample sizes are indicated in the figure legends. Critical experiments have high n numbers and were repeated multiple times. For in vivo experiments, we always used as many mice per group as possible in an attempt to minimize errors. For in vitro experiments where replicate samples were used, we repeated the experiments at least 2 independent times to confirm the findings.
Data exclusions	No data were excluded from the analysis.
Replication	This is indicated in the figure legends. On the graphs, individual dots represent individual samples/mice used. For each experiment, all attempts at replication were successful and our findings showed comparable results. Most experiments were replicated 2-6 independent times.
Randomization	In animal experiments in vivo, mice were randomly allocated into each experimental group based on their genotype. Given that our mice were inbred and matched for age and sex, we always assumed similar variances between the different experimental groups. For in vitro experiments, samples were randomized wherever possible.
Blinding	For in vivo experiments, the investigators were not blinded to allocation during experiments and outcome assessment. Blinding was not possible as the mice had to be genotyped by PCR before the analysis. For in vitro experiments, the investigators were not blinded due to the low probability of bias. Quantification of images was performed blindly using automated software (Fiji), wherever possible.

Reporting for specific materials, systems and methods

We require information from authors about some types of materials, experimental systems and methods used in many studies. Here, indicate whether each material, system or method listed is relevant to your study. If you are not sure if a list item applies to your research, read the appropriate section before selecting a response.

Materials & experimental systems

n/a	Involved in the study	
☐	☒	Antibodies
☐	☒	Eukaryotic cell lines
☒	☐	Palaeontology and archaeology
☐	☒	Animals and other organisms
☒	☐	Clinical data
☒	☐	Dual use research of concern
☒	☐	Plants

Methods

n/a	Involved in the study	
☒	☐	ChIP-seq
☐	☒	Flow cytometry
☒	☐	MRI-based neuroimaging

Antibodies

Antibodies used

For FACS
CD44 (IM7) / 103032 / Biolegend /1:200
CD16/32 (2.4G2) / 553142 / BD Pharmingen / 1:100
CD31 (MEC13.3) / 102510 /Biolegend / 1:300
Podoplanin (8.1.1) / 127416 / BioLegend / 1:300
CD45 (IM7) / eBioscienceTM / 12-0451-83 / 1:400
EPCAM (G8.8) / 118214 / BioLegend / 1:800

For Immunohistochemistry/Immunofluorescence:
biotinylated RANK (polyclonal)/ BAF692 / R&D systems/ 1:50
biotinylated RANKL (IK22/5) / 13-5952-82 / Invitrogen / 1:150
PDGFRa (polyclonal) / AF1062 / R & D Systems / 1:150
donkey anti-Goat Alexa Fluor 555 (polyconal) / A21432 / Invitrogen / 1:500
mouse OLFM4 (D6Y5A) / 39141 / Cell Signaling Technology / 1:800
human OLFM4 (D1E4M) / 14369 / Cell signaling Technology / 1:100
goat anti-Rabbit Alexa Fluor 633 (polyclonal) / A21072 / Invitrogen / 1:500
EpCAM AlexaFluor 488 (G8.8) / 118210 / Biolegend / 1:100

phospho-histone H3 (BC37) / PBC-ACI3130C / CellPath / 1:100
Cleaved caspase-3 (polyclonal) / 9661 / Cell Signaling Technology / 1:100
CRE (D7L7L) / 15036 / Cell Signaling Technology / 1:100
Glycoprotein 2 (2F11-C3) / D278-3 / MBL Life Science / 1: 150
HRP-polymer rabbit / PD000POL-K / DCS / ready-to-use
DAB ab64238/Abcam

For Westernblotting primary; 1:1,000, secondary; 1:5,000
β-actin (AC-74) / A5316 / Sigma / 1:1,000
IκBα (112B2) / 9247 / Cell Signaling Technology/ 1:1,000
phospho-IκBα (Ser32/36) (5A5) / 9246 / Cell Signaling / 1:1,000
anti-rabbit IgG HRP (polyclonal) / NA9340V / GE Healthcare / 1:5,000
anti-mouse IgG HRP (polyclonal) / W4021 / Promega / 1:5,000

Validation

All the antibodies used are commercially available and were validated by the manufacturers. The validation statement were found in the manufacturer's website.
The RANK antibody was confirmed using KO cells for specificity.

Eukaryotic cell lines

Policy information about cell lines and Sex and Gender in Research

Cell line source(s)

Human intestinal organoid lines were generated from intestinal biopsy specimens collected from the duodenum of children undergoing diagnostic endoscopy. This study was conducted with informed patient and/or caretaker consent as appropriate and with full ethical approval (REC-12/EE/0482).

Authentication

The organoid lines were not authenticated.

Mycoplasma contamination

Organoid lines were monthly tested for mycoplasma contamination and tested negative without exception.

Commonly misidentified lines
(See ICLAC register)

No commonly misidentified cell lines were used.

Animals and other research organisms

Policy information about studies involving animals; ARRIVE guidelines recommended for reporting animal research, and Sex and Gender in Research

Laboratory animals

Rank conditional mice (Rankflox) were generated in our laboratory and have been previously described. The following additional mouse strains were used: Rankl conditional mice (Ranklflox), Traf6 conditional mice (Traf6flox), constitutively active RANK mutant over-expressing mice (caRANKLSL), Rnf43 conditional mice (Rnf43flox) and Znrf3 conditional mice (Znrf3flox). and Vil-Cre mice. Apcmin/+ mice, Twist2-Cre mice, Cd4-Cre mice, Rorgt-Cre mice, tdTomato reporter mice, and Lgr5-eGFP-IRES-CreERT2 mice were purchased from the Jackson laboratories. All mouse lines were maintained on a C57BL/6J genetic background and housed under specific pathogen-free conditions. Mouse cages were individually ventilated and subjected to ambient temperature of 22°C ± 1°C and humidity of 55% ± 5% at a light/dark cycle of 14 h:10 h. The age-matched littermates mice aged at 3-25 wks old were used, unless otherwise stated. Mouse genotypes were assessed by PCR. For all experiments, only littermate and sex-matched mice were used unless otherwise specified. Control littermates of caRANKvil-Tg mice were defined as either Vil1-Cre mice, heterozygous caRANKLSL mice, or wild-type mice (negative for VilCre and negative for caRANKLSL). We did not observe any apparent differences among control littermates with different genotypes in any experiments. To exclude the potential effects of the Rank deletion in the intestine in timed pregnancy/lactation studies, RankWT and RankVil mutant female littermates were crossed to wild-type syngeneic C57BL/6J male breeders, resulting in RANK-sufficient fetuses with a comparable genetic background. Germ-free mice maintained at the University of British Columbia and Kiel University were used for the experiments. Timed matings were performed in germ-free and SPF mice to achieve syngenic (both parents C57 BL/6) and semiallogenic breedings (male BALB/C, female C57 BL/6).

Wild animals

No wild animal was used in this study.

Reporting on sex

Male littermate mice were used in the experiments designed to investigate the effects of malnutrition, including metabolic studies, as shown in Figure 4 and extended Figure 14.
Data of human intestinal organoids derived from one healthy female donor were used in Figure 5 and Extended Data Fig. 15.

Field-collected samples

No field-collected samples were used in this study.

Ethics oversight

All mice were group housed under specific pathogen-free conditions. All mice were bred, maintained, examined, and euthanized in accordance with institutional animal care guidelines and ethical animal license protocols approved by the legal authorities. All experimental animal projects were approved by the Federal Ministry of Education, Science, and Research at the Institute of Molecular Biotechnology of the Austrian Academy of Sciences (IMBA), Vienna BioCenter (VBC), the University of British Columbia Animal Care Committee at the University of British Columbia, or the committee for animal welfare of the state of Schleswig-Holstein (acceptance no.: V242-7224.121-33) at Kiel University.

Note that full information on the approval of the study protocol must also be provided in the manuscript.

Plants

Seed stocks | *Report on the source of all seed stocks or other plant material used. If applicable, state the seed stock centre and catalogue number. If plant specimens were collected from the field, describe the collection location, date and sampling procedures.*

Novel plant genotypes | *Describe the methods by which all novel plant genotypes were produced. This includes those generated by transgenic approaches, gene editing, chemical/radiation-based mutagenesis and hybridization. For transgenic lines, describe the transformation method, the number of independent lines analyzed and the generation upon which experiments were performed. For gene-edited lines, describe the editor used, the endogenous sequence targeted for editing, the targeting guide RNA sequence (if applicable) and how the editor was applied.*

Authentication | *Describe any authentication procedures for each seed stock used or novel genotype generated. Describe any experiments used to assess the effect of a mutation and, where applicable, how potential secondary effects (e.g. second site T-DNA insertions, mosiacism, off-target gene editing) were examined.*

Flow Cytometry

Plots

Confirm that:

☒ The axis labels state the marker and fluorochrome used (e.g. CD4-FITC).

☒ The axis scales are clearly visible. Include numbers along axes only for bottom left plot of group (a 'group' is an analysis of identical markers).

☒ All plots are contour plots with outliers or pseudocolor plots.

☒ A numerical value for number of cells or percentage (with statistics) is provided.

Methodology

Sample preparation | Please refer to the methods section.

Instrument | Data was acquired using LSR FortessaTM from BD Bioscience, and sorting of cells were performed using FACSAria III Cell Sorter from BD Bioscience.

Software | Data was analyzed using FlowJo v10.8.1 software (Tree Star)

Cell population abundance | Percentage of cell subtypes are displayed on FACS panel. All the samples were measured with the same speed to get raw counts.

Gating strategy | To gate samples for FACS analysis, cell were initially gated by FSC-A vs SSC-A for the exclusion of debris. For single cells, samples were further gated by SSC-H vs SSC-W. Live cells were finally gated and identified by using a fixable dye, APC-Cy7. After that, specific gating was performed for the population of interest (described in figure legend and/or methods sections).

☒ Tick this box to confirm that a figure exemplifying the gating strategy is provided in the Supplementary Information.
